# CPL on/off switching by enantiomer encapsulation in TPE heterochiral molecular cages

**DOI:** 10.1039/d5sc05405b

**Published:** 2025-09-29

**Authors:** Wei Yu, Ming Hu, Xin Wen, Zhi-Rong Xu, Minghua Liu, Yan-Song Zheng

**Affiliations:** a Key Laboratory of Material Chemistry for Energy Conversion and Storage, Ministry of Education, School of Chemistry and Chemical Engineering, Huazhong University of Science and Technology Wuhan 430074 China zyansong@hotmail.com; b Beijing National Laboratory for Molecular Science (BNLMS), CAS Key Laboratory of Colloid Interface and Chemical Thermodynamics, Institute of Chemistry, Chinese Academy of Sciences Beijing 100190 China

## Abstract

Chiral molecular cages have exhibited potential in chiral recognition, chiroptical materials, *etc.* They are generally obtained by introduction of chiral groups into linkers. Chiral cages derived from chiral lids are very rare. Here, enantiomerically pure hindered tetraphenylethene (hTPE) was used as a lid to synthesize chiral TPE cages. While the same helical-handed hTPE units are exploited as lids to give homochiral cages, heterochiral cages with two lids having inverse helical directions are obtained when one hTPE unit and one simple TPE unit are used as lids. Due to the homochirality of the two lids, the homochiral cages display 3-fold stronger circularly polarized luminescence (CPL) than the heterochiral cages. However, the heterochiral cages can adaptively include aromatic guest molecules whereas the homochiral cages fail to do so, thanks to the greater flexibility of TPE compared to the hTPE unit. Very exceptionally, one enantiomer of the chiral guest induces opposite helical chirality between the TPE lid and hTPE lid, turning off the CPL whereas the other enantiomer induces the same helical chirality of the two lids, maintaining or enhancing the CPL signal and furnishing a novel CPL switch. This discrimination between two enantiomers can be carried out using fluorescence spectra, and can even be applied to enantiomer excess (ee%) determination of chiral diacids. Furthermore, through adaptive inclusion, chiral energy transfer between the heterochiral cages and achiral dyes such as Eosin Y occurs, leading to CPL multi-color emission from the achiral dyes.

## Abstract 2

Conformation-immobilized tetraphenylethylene is exploited, for the first time, as chiral lid in construction of chiral molecular cages. The prepared chiral cages display potential in CPL switch, chiral analysis, chiral transfer, and energy transfer.

## Graphical abstract

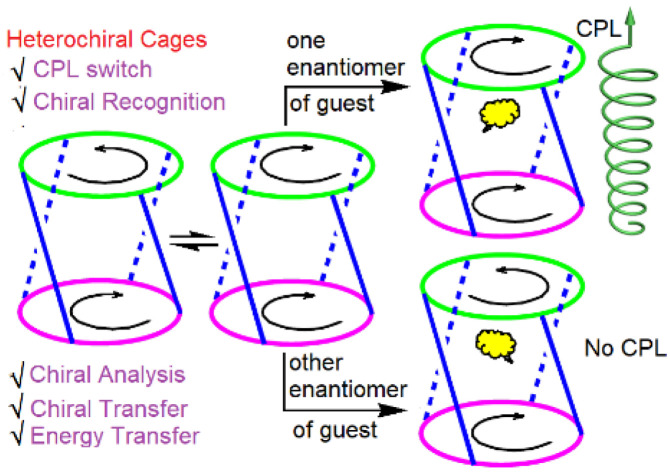

## Introduction

Recently, chiral molecular cages have attracted increasing interest^1–12^ due to their great potential in chiral recognition,^1,2^ chiroptical materials,^3,4^ spin filtering,^5^ chiral regulated gas separation,^6^*etc.* These chiral molecular cages are generally obtained by the introduction of chiral groups into linkers. By symmetry breaking^7^ or interweaving cage-catenanes with topological chirality,^8^ chiral cages could be obtained. In addition, inherent chiral cages can be formed if each lid and each linker composing cage are different.^9^ However, using enantiomerically pure lids to construct chiral cages is very rare^10^ although lids bearing flexible propeller-like conformations^11^ and rotating faces^12^ have been used to construct chiral cages.

Tetraphenylethylene (TPE) and its derivatives have been widely used as aggregation-induced emission (AIE) molecules due to their propeller-like conformation.^13^ The fundamental TPE unit is also widely used to construct molecular cages as lids together with linkers.^14,15^ Up to now, a large number of reported TPE cages have exhibited excellent photophysical properties due to the AIE effect.^14,15^ To obtain chiroptical materials, some chiral TPE cages have also been designed and synthesized, which are all prepared by using chiral linkers. For example, by the condensation reaction of TPE aldehydes with optically pure *trans*-1,2-cycloheanediamines (CHDA)^16–20^ or CHDA-containing linkers,^21^ TPE imine cages and their reductive TPE amine cages are obtained. By forming disulfide bonds, chiral TPE cages with amino acid residues linkers are prepared.^22^ Notably, by condensation of TPE tetraldehyde and achiral triamine, rigid 6 + 8 TPE cages are obtained, which limit the free rotation of TPE phenyl rings, enabling the separation of enantiomerically pure TPE cages by chiral HPLC.^23^ In addition, the left-handed (*M*) and right-handed (*P*) propeller-like conformations of the TPE unit are dynamically interconvertible, enabling its binding to chiral molecules to exhibit adaptive chirality. Recently, Cao *et al.* reported that by incorporating chiral guests such as nucleotides into TPE-based octacationic cages, propeller-like chiral signals of TPE units could be induced in the cages.^24–26^ More directly, the propeller-like conformation of the TPE unit could be immobilized by intramolecular cyclization or by introducing two methyl groups at the *ortho*-positions of TPE phenyl rings (hindered TPE, hTPE).^27,28^ If TPE units with conformation immobilization are used as cage lids, more stable and diverse chiral TPE cages will be obtained. However, up to now, the use of TPE units with immobilized conformations to construct chiral TPE cages has not been reported.

Herein, chiral cages composed of chiral hTPE lids and achiral linkers are synthesized. This novel class of TPE cages shows tilted linkers relative to the TPE unit in one direction, endowing the whole cage with helical chirality. When one hTPE unit and one simple TPE unit are used as lids, the resulting heterochiral cages with two lids having inverse helical directions are obtained. Moreover, the chiral cages display strong circularly polarized luminescence (CPL) emission, chiral recognition ability, and chiral energy transfer properties.

## Results and discussion

**Scheme 1:**
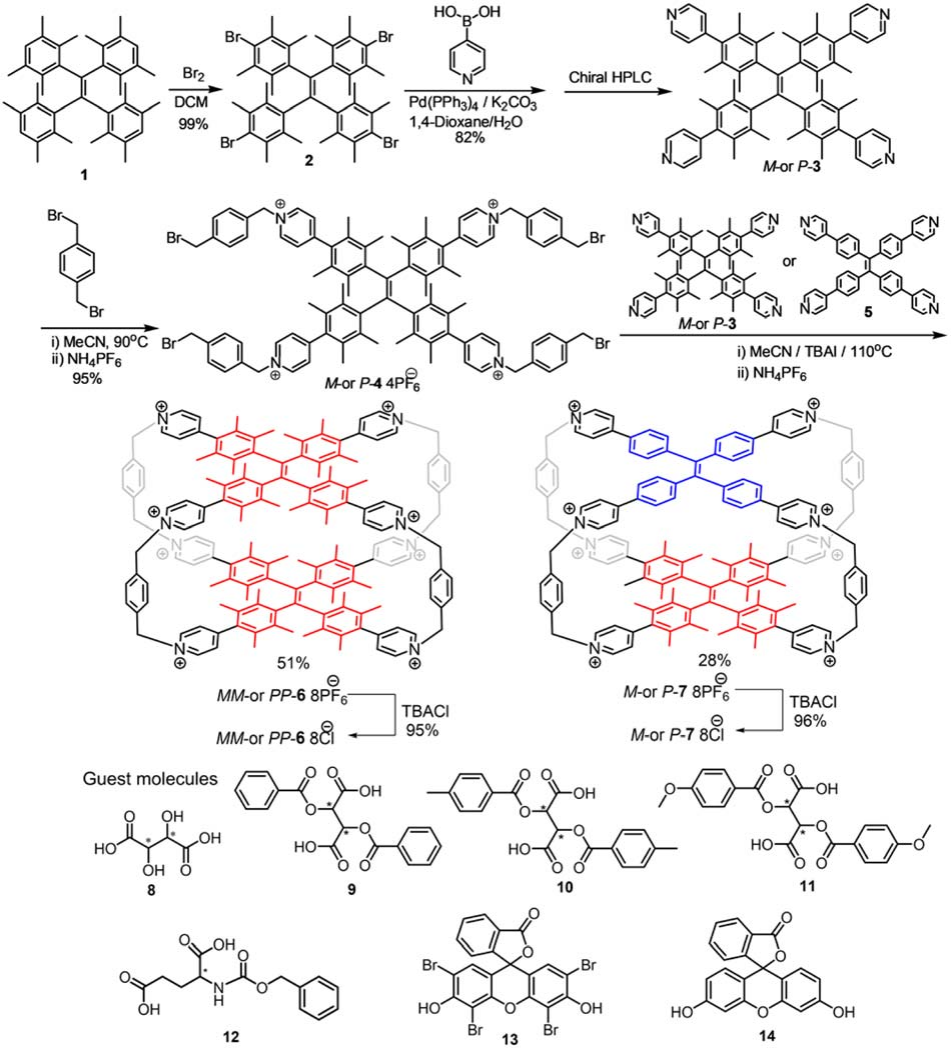
Synthesis routes of *PP*/*MM*-6·8PF_6_^−^, *PP*/*MM*-6·8Cl^−^, *P*/*M*-7·8PF_6_^−^ and *P*/*M*-7·8Cl^−^ and chemical structures of guest molecules 8–14.

Homochiral and heterochiral TPE cages were synthesized as shown in Scheme 1 following the literature procedure.^24^ Through bromination with liquid bromine, the starting compound hTPE 1 (ref. 28) was quantitatively converted into hTPE tetrabromide 2, which yielded hTPE tetrapyridine 3 by a Suzuki coupling reaction with 4-pyridinylboronic acid. Compound 3 can be resolved by chiral HPLC into two stable and pure enantiomers *M*-3 and *P*-3 (Fig. S79). By substitution of 3 with an excess of 1,4-bis(bromomethyl)benzene, the half-cage structure 4·4PF_6_^−^ was synthesized in 95% yield. At the last step, 4·4PF_6_^−^ reacted with hTPE tetrapyridine 3 or TPE tetrapyridine 5 in a 1 : 1 molar ratio to furnish homochiral TPE cages 6·8PF_6_^−^ in 51% yield or 7·8PF_6_^−^ in 28% yield after purification by column chromatography using NH_4_PF_6_ solution as the eluent. The cages 6·8PF_6_^−^ and 7·8PF_6_^−^ were transformed into 6·8Cl^−^ or 7·8Cl^−^*via* anion exchange with excess tetrabutylammonium chloride. Starting from either *M*-3 or *P*-3, enantiomerically pure cages *MM*/*PP*-6·8PF_6_^−^, *MM*/*PP*-6·8Cl^−^ and *M*/*P*-7·8Cl^−^ can be obtained using the same procedure. These new compounds were fully characterized by NMR, MS, and IR spectroscopy and optical rotation (Fig. S1–S78).

The ^1^H NMR spectrum of TPE cage 6·8Cl^−^ prepared from racemic 3 showed two sets of signals while *MM*-6·8Cl^−^ and *PP*-6·8Cl^−^ showed only one set of signals (Fig. S80). Therefore, one set of proton signals arises from two enantiomers of 6·8Cl^−^ and other set of proton signals is ascribed to the mesomer *MP*-6·8Cl^−^. The integration ratio of the two enantiomers to the mesomer was 1 : 1, which was in accordance with the theoretical expectation based on the reaction ratio of racemic 3. Similar results were also observed in the ^1^H NMR spectrum of 6·8PF_6_^−^. In contrast, due to the flexible conformation of the TPE unit, 7·8Cl^−^ did not display two sets of proton signals when racemic 3 was used as the reactant because it only gave a racemic mixture of *M*-7·8Cl^−^ and *P*-7·8Cl^−^ without forming an additional mesomer. The ^1^H–^1^H NOESY NMR spectra of *PP*-6·8Cl^−^ and *P*-7·8Cl^−^ confirmed the above result (Fig. S77 and S78).

**Fig. 1:**
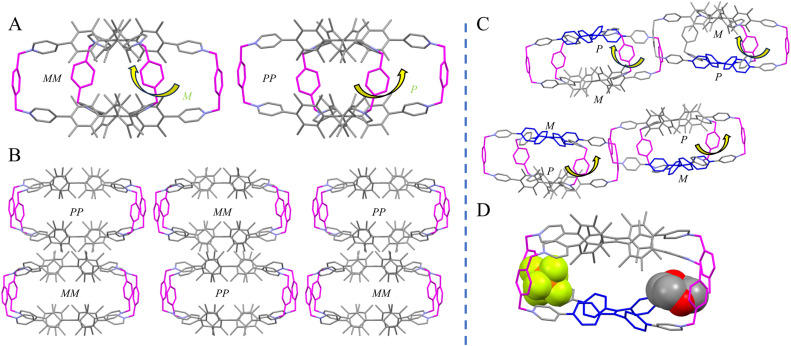
Crystal structures (A) and molecule stacking in one crystal cell (B) of 6·8PF_6_^−^; PF_6_^−^ ions, solvents and hydrogen atoms are omitted for clarity except the included PF_6_^−^ (yellow) in B. The helical direction of cage 7·8PF_6_^−^ in one crystal cell (C) and inclusion of dioxane molecules and counter ions PF_6_^−^ (yellow) (D) in 7·8PF_6_^−^; hydrogen atoms and other non-included PF_6_^−^ and solvents are omitted for clarity.

Fortunately, single crystals of 6·8PF_6_^−^ suitable for X-ray diffraction were obtained through the vial-in-vial method by slowly diffusing diethyl ether into an acetonitrile solution of 6·8PF_6_^−^ for 3 days. Crystal diffraction analysis revealed that 6·8PF_6_^−^ is a 2 + 4 cage composed of two hTPE tetrapyridinium units and four *p*-phenylenedimethylene linkers (Fig. 1A). However, only homochiral *MM*- and *PP*-6·8PF_6_^−^ enantiomeric cages in equal amounts were observed, and no mesomer *MP*-6·8PF_6_^−^ was observed, which is inconsistent with the ^1^H NMR spectrum. This is likely because the racemate preferentially crystallized and the mesomer still remained in solution due to their different solubilities. Unexpectedly, the angle between the linkers and the hTPE lids was not 90° but 76.6°, showing tilted four linkers in one direction. This twisted structure imparts helical chirality to the entire molecular cage, in contrast to the achiral TPE cages.^24–26^ While the four *p*-phenylenedimethylene linkers were arranged in a left-handed helical arrangement in the *MM*-6·8PF_6_^−^ cages, this direction was right-handed helical in the *PP*-6·8PF_6_^−^ cages, which was consistent with the helical direction of the hTPE units (Fig. 1A). The molecules with *MM*- and *PP*-configurations were stacked alternately (Fig. 1B). Although 6·8PF_6_^−^ possesses a large volume with approximate dimensions of 17.6 × 10.7 × 7.7 Å^3^ (Fig. S81A), the shortest distance between the methyl hydrogen atoms of the two hTPE units is only about 2.6 Å, showing almost no cavity between the two hTPE units. However, the four windows around hTPE, pyridinium, and linkers are large. Therefore, one PF_6_^−^ counter anion was included into the window at *cis*-position of the double bond (Fig. 1B).

The crystal structure of 7·8PF_6_^−^ showed that it is a 1 + 1 + 4 cage composed of one hTPE unit, one TPE unit and four *p*-phenylenedimethylene linkers. Notably, the helical direction of the hTPE unit is opposite to that of the TPE unit, that is, *M*-hTPE induces *P*-TPE conformation and *P*-hTPE leads to *M*-TPE conformation. Therefore, the molecular cage 7·8PF_6_^−^ is heterochiral. Furthermore, the four linkers were tilted in the same direction and even exhibited a larger tilt angle between the cage lids and linkers from 63° to 77° compared to the homochiral cage 7·8PF_6_^−^, indicating a larger twist for the heterochiral cage. As with 6·8PF_6_^−^, the helical direction of the linker tilt is determined by the helical direction of hTPE. Consequently, the whole heterochiral cage also exhibits helical chirality (Fig. 1C). The dimensions of 7·8PF_6_^−^ are 17.4 × 12.4 × 7.1 Å^3^ (Fig. S81B). The window width of 12.4 Å at the *cis*-position of the hTPE unit is obviously larger than that of 6·8PF_6_^−^ (10.7 Å) because not only 8PF_6_^−^ but also the solvent dioxane molecule is deeply included into this window at the *cis*-position (Fig. 1D). Although the shortest distance between a methyl hydrogen of the hTPE unit and a phenyl hydrogen of the TPE unit is only about 2.6 Å, this distance would probably be increased with environmental changes due to the flexibility of the TPE unit.

Due to the AIE effect and restricted rotation of of phenyl rings in both the hTPE units and the cages, these chiral TPE cages emit strong fluorescence both in the solid state and in solution. In addition, because of the D–A effect of methyl groups and ammonium groups, the emission of homochiral cages 6·8PF_6_^−^ and 6·8Cl^−^ exhibited a typical solvent effect, with a maximum emission wavelength difference of 76 nm to 104 nm (Fig. S87) and a change in absolute fluorescence quantum yield (*Φ*_F_) from 0.8% to 69% in different solvents (Table S1). Due to the lack of an obvious D–A effect in the TPE unit, the heterochiral cages 7·8PF_6_^−^ and 7·8Cl^−^ exhibited a very small difference in maximum emission wavelength (Fig. S87) although distinct changes in*Φ*_F_ are observed with different solvents (Table S1).

**Fig. 2:**
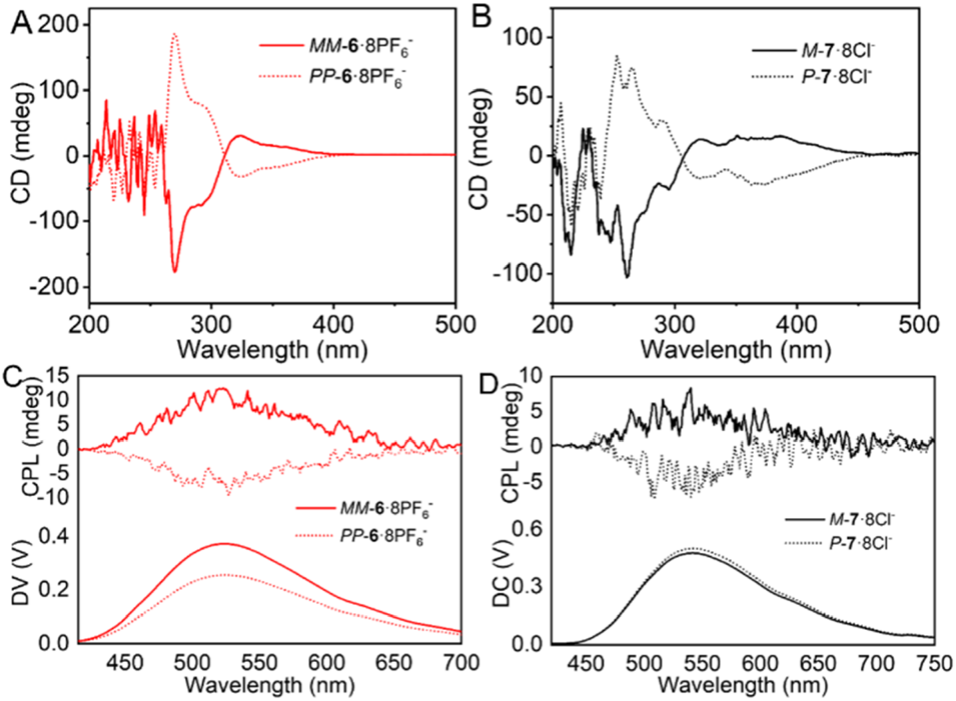
CD spectra of *MM*-6·8PF_6_^−^ and *PP*-6·8PF_6_^−^ (A) in MeCN and *P*-7·8Cl^−^ and *M*-7·8Cl^−^ (B) in H_2_O ([6·8PF_6_^−^] = [7·8Cl^−^] = 5.0 × 10^−4^ M). And CPL spectra of *PP*-6·8PF_6_^−^ and *MM*-6·8PF_6_^−^ (C) in MeCN/CHCl_3_ 10 : 90 (1.0 × 10^−3^ M) and *P*-7·8Cl^−^ and *M*-7·8Cl^−^ (D) in H_2_O (5.0 × 10^−4^ M).

With enantiomerically pure cages, homochiral *MM*-6·8PF_6_^−^ showed a positive Cotton effect while *PP*-6·8PF_6_^−^ showed a negative Cotton effect in the circular dichroism (CD) spectrum and these two CD spectra were perfect mirror images (Fig. 2A and S92). The absolute *g*_abs_ of *MM*/*PP*-6·8PF6^−^ was 1.2 × 10^−3^. Similarly, heterochiral cages *M*-7·8Cl^−^ showed a positive Cotton effect while *P*-7·8Cl^−^ showed a negative Cotton effect with perfect mirror symmetry between these two CD spectra (Fig. 2B and S93), and the corresponding absolute *g*_abs_ was 6.1 × 10^−4^, demonstrating that the CD direction is controlled by hTPE chirality instead of induced TPE chirality.

Moreover, homochiral cages *MM*-6·8PF_6_^−^ and *PP*-6·8PF_6_^−^emitted strong CPL at 525 nm in MeCN/CHCl_3_ 10 : 90 (volume ratio, the same below) with dissymmetric factors (*g*_lum_) of up to +2.28 × 10^−3^ and −1.91 × 10^−3^, respectively (Fig. 2C and S94). For heterochiral cages, *M*-7·8Cl^−^ and *P*-7·8Cl^−^ could emit CPL even in water at 542 nm with *g*_lum_ values of 6.12 × 10^−4^ and −7.99 × 10^−4^, respectively (Fig. 2D and S95). The CPL direction is in accordance with that of CD signals. Due to the inverse helical directions of hTPE and TPE units, heterochiral cages exhibit only one-third of the CPL intensity of homochiral cages.

**Fig. 3:**
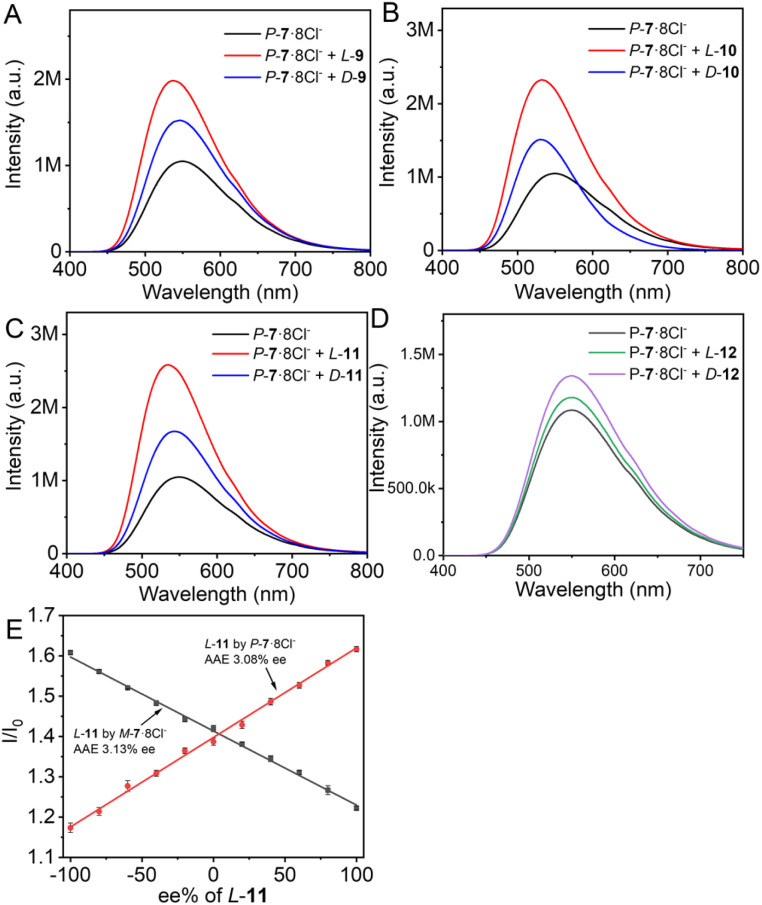
Fluorescence spectra of *P*-7·8Cl^−^ in the presence of *L*/*D*-9 (A), *L*/*D*-10 (B), *L*/*D*-11 (C), and *L*/*D*-12 (D) in H_2_O/CH_3_OH 99 : 1. (2[*P*-7·8Cl^−^] = [9] = [10] = [11] = [12] = 2.0 × 10^−5^ M). (E) Change in *I*/*I*_0_ of *M*-7·8Cl^−^ and *P*-7·8Cl^−^ with ee% of *L*-11. [*M*-7·8Cl^−^] = [*P*-7·8Cl^−^] = [*L*-11] = 1.0 × 10^−5^ M; *λ*_ex_ = 350 nm.

In addition, the chiral TPE cages were tested for chiral recognition and chiral transfer. When homochiral cages *MM*-6·8PF_6_^−^ and *PP*-6·8PF_6_^−^ as well as *MM*-6·8Cl^−^ and *PP*-6·8Cl^−^ interacted with guest molecules 8−14 (Scheme 1), no changes in absorption, emission and CD spectra were observed in various solvents. In contrast, when heterochiral cages 7·8Cl^−^ were used for the test, tartaric acid (TA) derivatives, dibenzoyl TA 9, di-*p*-toluoyl TA 10, and di-*p*-anisoyl TA 11, exhibited obvious fluorescence enhancement in water except for TA itself 8 (Fig. 3A–C). Moreover, enantiomerically pure heterochiral cages displayed different fluorescence enhancements for the two enantiomers of TA derivatives. While stronger fluorescence of *M*-7·8Cl^−^ was induced by *D*-9 than by *L*-9, more emission enhancement of *P*-7·8Cl^−^ was induced by *L*-9 than by *D*-9, indicating that the fluorescence difference was caused by chiral recognition. For 10 and 11, similar results of chiral recognition were also obtained. Using *P*-7·8Cl^−^ as a chiral probe, the ratio of fluorescence intensity differences between the two enantiomers ((*I*_*L*_ − *I*_0_)/(*I*_*D*_ − *I*_0_); *I*_*D*_ or *I*_*L*_, intensity of *P*-7·8Cl^−^ with guest enantiomer; *I*_0_, emission intensity without guest) was 1.98, 2.74 and 2.46 for 9, 10 and 11, respectively. For *N*-benzyloxycarbonylglutamic acid 12, the heterochiral cages could also recognize its enantiomers, and the intensity ratio was up to 2.71 with *M*-7·8Cl^−^ (Fig. 3D).

Furthermore, chiral recognition by the heterochiral cages could be used to determine the enantiomeric purity of chiral guests. For example, by maintaining a 1 : 1 molar ratio of *P*-7·8Cl^−^ or *M*-7·8Cl^−^ to a mixture of *L*-11 and *D*-11, the fluorescence intensity was linearly related to the ee% of *L*-11 ranging from −100% to 100% (Fig. 3E). The resulting straight line could serve as a calibration curve for assessing the enantiomer purity of 11 with unknown ee%. The average absolute error (AAE) between the measured ee values and the actual ee values was 3.08% ee using *P*-7·8Cl^−^ as the chiral receptor and 3.13% ee using *M*-7·8Cl^−^, which are comparable to those of previously reported chiral fluorescent probes for ee determination in dilute solution.^29,30^ Therefore, the heterochiral cages show great potential for high throughput ee analysis of chiral diacids.

**Fig. 4:**
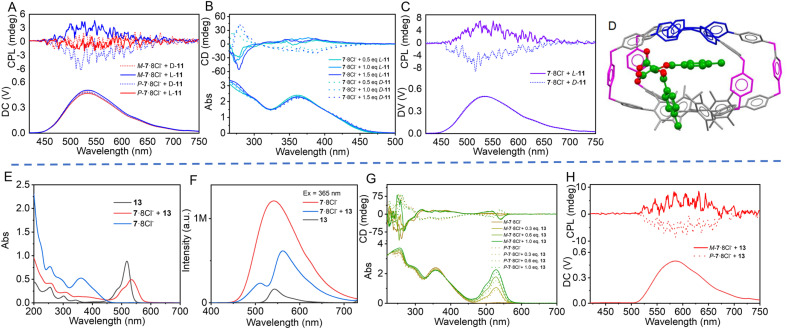
(A) CPL spectra of *M*-7/*P*-7·8Cl^−^ with 2.0 eq. of enantiomers of 11 in water. (B) CD and absorption spectra of racemic 7·8Cl^−^ with *L*/*D*-11 in H_2_O. (C) CPL spectra of 7·8Cl^−^ with 3.0 eq. of *L*/*D*-11 in H_2_O. (D) Schematic diagram of *M*-7·8Cl^−^⊃*D*-10 complex obtained using HyperChem software. *M*-[7·8Cl^−^] = 5.0 × 10^−4^ M. (E) and emission spectra (F) of 7·8Cl^−^, Eosin Y 13, and their mixture in H_2_O. (*λ*_ex_ = 365 nm, [7·8Cl^−^] = [13] = 1.0 × 10^−5^ M). (G) CD spectra and (H) CPL spectra (1 : 1) of *M*/*P*-7·8Cl^−^ with 13 in H_2_O. ([*P*-7·8Cl^−^] = [*M*-7·8Cl^−^] = 5.0 × 10^−4^ M).

More interestingly, the two enantiomers could be recognized by the heterochiral cages *via* CPL spectra. The CPL signal remained almost unchanged when *M*-7·8Cl^−^ interacted with *L*-11 in water, but the CPL signal disappeared upon mixing of *M*-7·8Cl^−^ with *D*-11. On the other hand, using *P*-7·8Cl^−^ as the chiral receptor, *D*-11 left the CPL signal unchanged whereas *L*-11 caused the CPL to disappear (Fig. 4A). Meanwhile, the CD differences induced by the two enantiomers was also observed. While the CD signal of *M*-7·8Cl^−^ decreased slightly upon adding *L*-11, the decrease was more pronounced when *D*-11 was added (Fig. S98). This result suggests that the interaction of *M*-7·8Cl^−^ with *D*-11 and *P*-7·8Cl^−^ with *L*-11 was much stronger than that of *M*-7·8Cl^−^ with *L*-11 and *P*-7·8Cl^−^ with *D*-11. The strong interaction of *M*-7·8Cl^−^ with *D*-11 and *P*-7·8Cl^−^ with *L*-11 induced inverse helical chirality of the TPE unit relative to the hTPE unit, resulting in the disappearance of CPL. In contrast, the interaction of *M*-7·8Cl^−^ with *L*-11 and *P*-7·8Cl^−^ with *D*-11 was so weak that almost no change occurred in CPL intensity.

When racemic 7·8Cl^−^ interacted with *D*-11 and *L*-11 in water, *L*-11 led to a positive Cotton effect and *D*-11 produced a negative one in the CD spectra (Fig. 4B). Notably, racemic 7·8Cl^−^ exhibited a positive CPL signal in the presence of *L*-11 while a negative CPL signal was observed with *D*-11 (Fig. 4C) at 530 nm in water. The absolute *g*_lum_ value was 6.0 × 10^−4^, being about one-third that of the homochiral cages. Similar results were obtained with 9 and 10 (Fig. S99–101). Probably due to the strong interaction of *M*-7·8Cl^−^ with *D*-11, the positive CD and CPL signals of *M*-7·8Cl^−^ in the racemic mixture of 7·8Cl^−^ were weakened and even disappeared. Therefore, the negative signals of *P*-7·8Cl^−^ stood out. Conversely, *L*-11 led to a decrease in the negative signals of *P*-7·8Cl^−^ in the racemate, so that the positive signals of *M*-7·8Cl^−^ became apparent. These results are consistent with the tests using enantiomerically pure 7·8Cl^−^ described above.

The ^1^H NMR analysis of 7·8Cl^−^ with *D*-11 revealed that two sets of protons on the phenyl ring of *D*-11 shifted upfield by 0.13 ppm and 0.07 ppm, respectively, due to the shielding effect of 7·8Cl^−^. The ^1^H NMR spectrum of 7·8Cl^−^ remained almost unchanged, except for a slight upfield shift (by 0.01 ppm) of the methyl peak (Fig. S102). This suggests that the phenyl ring of *D*-11 entered the internal cavity of 7·8Cl^−^ due to adaptive structural transformation (Fig. 4D). UV-Vis titration disclosed that the binding ratio of the guests 9, 10, or 11 to 7·8Cl^−^ was 1 : 1 (Fig. S107–S109). The binding constants of *M*-7·8Cl^−^ with the two enantiomers of 9, 10, and 11 (*K*_D_/*K*_L_) were 1.2 × 10^5^/6.8 × 10^4^, 1.9 × 10^5^/9.8 × 10^4^, and 5.5 × 10^5^/1.2 × 10^5^ M^−1^, respectively. These results confirm that the d-enantiomer showed stronger binding ability to *M*-7·8Cl^−^ than the l-enantiomer, and the binding ability increased from 9, 10, to 11 with the increasing electron-donating capacity of the phenyl ring connected to TA.

Given that the heterochiral cage can accommodate an aromatic ring, achiral Eosin Y 13 and fluorescein 14 were chosen for the chirality transfer test because they have a carboxylic isomer that can bind the cationic cage in addition to host–guest interaction. UV-Vis spectra disclosed that the absorption bands of Eosin Y at 484 nm and at 517 nm were bathochromically shifted to 504 nm and 532 nm, respectively, when it was mixed with 7·8Cl^−^ (Fig. 4E), suggesting that the spiro-ring of 13 was opened. By titration of Eosin Y with the 7·8Cl^−^ cage, a 1 : 1 host–guest complex between 7·8Cl^−^ and Eosin Y was formed, with an association constant of 1.6 × 10^5^ M^−1^ (Fig. S110 and S111). Emission spectra revealed that the fluorescence of the cage was significantly attenuated and exhibited a hypochromic shift from 540 nm to 510 nm, whereas the emission of Eosin Y increased and showed a bathochromic shift from 542 nm to 560 nm, when they were mixed and excited by 365 nm light in water (Fig. 4F), indicating energy transfer from the cage to Eosin Y. Importantly, CD spectral measurement showed that upon the addition of Eosin Y to the solution of *M*-7·8Cl^−^ in water, a positive Cotton effect was observed at 540 nm and a negative Cotton effect was observed at 510 nm, both originating from Eosin Y. In comparison, when Eosin Y was added to *P*-7·8Cl^−^, mirror-symmetric CD signals relative to those of *M*-7·8Cl^−^ were observed, confirming the successful chirality induction of Eosin Y by *P*/*M*-7·8Cl^−^ (Fig. 4G). The CD bisignate band demonstrated that the encapsulated Eosin Y was in a helical conformation. Moreover, in the presence of enantiomerically pure 7·8Cl^−^, Eosin Y exhibited obvious CPL signals at 580 nm in water, with a positive *g*_lum_ value of 4.86 × 10^−4^ for *M*-7·8Cl^−^ and a negative *g*_lum_ value of −5.36 × 10^−4^ (Fig. 4H).

Fluorescein 14 also formed a 1 : 1 host–guest complex with 7·8Cl^−^, having an association constant of 9.6 × 10^5^ M^−1^ (Fig. S112). Just like Eosin Y, the Cotton effect of 14 was mediated by *P*/*M*-7·8Cl^−^ and CPL at 550 nm in water was observed (Fig. S114 and S115). Because the homochiral cage 6·8Cl^−^ without free cavity space did not induce chirality in these achiral dyes, adaptive inclusion instead of ion pair interaction between the carboxylic anion and ammonium cations was crucial for chirality transfer.

^1^H NMR spectral changes between Eosin Y and *P*/*M*-7·8Cl^−^ further corroborated that the guest molecules entered the interior of the cage. Upon addition of Eosin Y, the overall peaks of methyl protons of 7·8Cl^−^ were slightly shifted to the upfield region by −0.005 to −0.01 ppm, and the protons on the pyridine ring far from the nitrogen atom also shifted to the upfield region by −0.01 ppm. For Eosin Y, the proton Hd signal was shifted to the upfield region by −0.01 ppm while the proton He signal was shifted to the downfield region by +0.015 ppm (Fig. S116).

## Conclusions

In conclusion, new chiral cages composed of chiral lids were synthesized. By using helical hTPE unit as lids, the resulting cages had tilted linkers in the same direction, endowing the whole cage with helical chirality. When both lids were identical hTPE units, homochiral cages were obtained, which could emit strong CPL. In contrast, when the two lids consisted of one hTPE unit and one TPE unit, the helical directions of both hTPE and TPE units were opposite, furnishing a heterochiral cage. The chirality of the heterochiral cage was governed by the helical direction of the hTPE unit, and it could also display CPL signals. While the homochiral cages were unable to accommodate guest molecules because of their too small cavity size, the heterochiral cages displayed adaptive encapsulation of aromatic guests due to the flexibility of the TPE unit. When the guest molecule was a chiral diacid, the heterochiral cage enabled the discrimination and quantitative analysis of the two enantiomers of the chiral diacid through fluorescence, CD, and CPL spectra. Moreover, the heterochiral cage could induce CD and CPL signals in achiral dyes, generating more colors of CPL emission. The chiral recognition was very unique because one enantiomer of a chiral guest induced opposite helical chirality between the TPE lid and hTPE lid, turning off the CPL signal whereas the other enantiomer induced the same helical chirality between the TPE lid and hTPE lid, retaining or enhancing the chiroptical signal. These results provide a new strategy for chiral induction and chiral recognition by constructing heterochiral cages.

## Author contributions

W. Yu (first author): writing – original draft, methodology, investigation, formal analysis, data curation; M. Hu, X. Wen, Z.-R. Xu and M. Liu: writing – review & editing; Y.-S. Zheng (corresponding author): writing – review & editing, supervision, project administration, methodology, funding acquisition, conceptualization. All authors have given approval to the final version of the manuscript.

## Conflicts of interest

The authors declare no competing financial interest.

## Supplementary Material

SC-OLF-D5SC05405B-s001

SC-OLF-D5SC05405B-s002

## Data Availability

CCDC 2453482 and 2453483 contain the supplementary crystallographic data for this paper.^31*a*,*b*^ The data that support the findings of this study are available within the article and the supplementary information (SI). Supplementary information: detail synthesis, characterization spectra, more measurements that support the findings of this study. See DOI: https://doi.org/10.1039/d5sc05405b.

## References

[cit1] Yue M.-S., Luo N., Wang X.-D., Ao Y.-F., Wang D.-X., Wang Q.-Q. (2025). J. Am. Chem. Soc..

[cit2] Walther A., Tusha G., Schmidt B., Holstein J. J., Schafer L. V., Clever G. H. (2024). J. Am. Chem. Soc..

[cit3] Shang W., Wang Y., Zhu X., Liang T., Du C., Xiang J., Liu M. (2023). J. Am. Chem. Soc..

[cit4] Ge C., Shang W., Chen Z., Liu J., Tang H., Wu Y., He S., Liu M., Li H. (2024). Angew. Chem., Int. Ed..

[cit5] Yu S., Yang S., Yang M., Yang J., Song Z., Hu D., Ji H., Jia Z., Liu M. (2025). Angew. Chem., Int. Ed..

[cit6] Wang Y., Zhang Y., Wang Y.-Y., Yan Q. (2025). J. Am. Chem. Soc..

[cit7] Chen C., Zhang S. (2025). Acc. Chem. Res..

[cit8] Chen L., Chen Z., Wang W., Chen C., Kuboi Y., Zhang C., Li C., Zhang S. (2024). J. Am. Chem. Soc..

[cit9] Fang S., Bao Z., Liu Z., Wu Z., Tan J.-P., Wei X., Li B., Wang T. (2024). Angew. Chem., Int. Ed..

[cit10] Benke B. P., Kirschbaum T., Graf J., Gross J. H., Mastalerz M. (2023). Nat. Chem..

[cit11] Wang Z., Zhang Q.-P., Guo F., Ma H., Liang Z.-H., Yi C.-H., Zhang C., Chen C.-F. (2024). Nat. Commun..

[cit12] Dong X., Qu H., Sue A. C.-H., Wang X.-C., Cao X.-Y. (2024). Acc. Chem. Res..

[cit13] Mei J., Leung N. L. C., Kwok R. T. K., Lam J. W. Y., Tang B. Z. (2015). Chem. Rev..

[cit14] Feng H.-T., Yuan Y.-X., Xiong J.-B., Zheng Y.-S., Tang B. Z. (2018). Chem. Soc. Rev..

[cit15] Li D.-M., Zuo R., Wang J., Le Z. (2025). Chem.–Eur. J..

[cit16] Bian L., Tang M., Liu J., Liang Y., Wu L., Liu Z. (2022). J. Mater. Chem. C.

[cit17] Sun Y.-L., Wang Z., Ma H., Zhang Q.-P., Yang B.-B., Meng X.-G., Zhang Y.-H., Zhang C. (2023). Chem. Comm..

[cit18] Wang Z., Wang W., Tang B., Sun L., Zhang F., Luo A. (2024). New J. Chem..

[cit19] Zhang H.-J., Lai Y.-L., Yang H., Zhou X.-C., Yuan Z.-J., Deng L., Hu X.-L., Li X., Zhou X.-P., Li D. (2024). Aggregate.

[cit20] Zheng X., Zhu W., Zhang C., Zhang Y., Zhong C., Li H., Xie G., Wang X., Yang C. (2019). J. Am. Chem. Soc..

[cit21] Feng X., Liao P., Jiang J., Shi J., Ke Z., Zhang J. (2019). ChemPhotoChem.

[cit22] Drożdż W., Bouillon C., Kotras C., Richeter S., Barboiu M., Clément S., Stefankiewicz A. R., Ulrich S. (2017). Chem.–Eur. J..

[cit23] Qu H., Wang Y., Li Z., Wang X., Fang H., Tian Z., Cao X. (2017). J. Am. Chem. Soc..

[cit24] Duan H., Li Y., Li Q., Wang P., Liu X., Cheng L., Yu Y., Cao L. (2020). Angew. Chem., Int. Ed..

[cit25] Cheng L., Liu K., Duan Y., Duan H., Li Y., Gao M., Cao L. (2020). CCS Chem..

[cit26] Li Q., Yan C., Zhang P., Wang P., Wang K., Yang W., Cheng L., Dang D., Cao L. (2024). J. Am. Chem. Soc..

[cit27] Xiong J.-B., Feng H.-T., Sun J.-P., Xie W.-Z., Yang D., Liu M., Zheng Y.-S. (2016). J. Am. Chem. Soc..

[cit28] Hu M., Ye F.-Y., Du C., Wang W., Yu W., Liu M., Zheng Y.-S. (2022). Angew. Chem., Int. Ed..

[cit29] Wu X., Han X., Xu Q., Liu Y., Yuan C., Yang S., Liu Y., Jiang J., Cui Y. (2019). J. Am. Chem. Soc..

[cit30] Wang F., Wang W., Wang Y., Zheng W., Zheng T., Zhang L., Okamoto Y., Shen J. (2023). Carbohydr. Polym..

[cit31] (a) CCDC 2453482: Experimental Crystal Structure Determination, 2025, 10.5517/ccdc.csd.cc2nc1lp

