# Supplementary material for: CPL on/off switching by enantiomer encapsulation in TPE heterochiral molecular cages
Source: Chem Sci. 2025 Sep 29;16(44):20924–30. doi: 10.1039/d5sc05405b (PMC12510256; doi:10.1039/d5sc05405b)
Supplement: SC-016-D5SC05405B-s001 [file SC-016-D5SC05405B-s001.pdf]

Supplementary Information (SI) for Chemical Science.
This journal is © The Royal Society of Chemistry 2025

Supporting Information

CPL On/Off Switched by Enantiomer Encapsulation in TPE Heterochiral Molecular Cages

Wei Yu,† Ming Hu,†* Xin Wen,‡ Zhi-Rong Xu,† Minghua Liu,‡ and Yan-Song Zheng†*

† Wei Yu, Dr. Ming Hu, Zhi-Rong Xu, Prof. Dr. Yan-Song Zheng, Key Laboratory of Material Chemistry for Energy Conversion and Storage, Ministry of Education, School of Chemistry and Chemical Engineering, Huazhong University of Science and Technology, Wuhan 430074, China. E-mail: zyansong@hotmail.com

‡ Xin Wen, Prof. Dr. Minghua Liu, Beijing National Laboratory for Molecular Science (BNLMS), CAS Key Laboratory of Colloid Interface and Chemical Thermodynamics, Institute of Chemistry, Chinese Academy of Sciences, Beijing 100190, China.

Materials and Methods

Materials. All reagents and solvents were chemical pure (CP) grade or analytical reagent (AR) grade and were bought from China National Pharmaceutical Group Corporation, Aladdin (Shanghai) Bio-Chem Technology Co Ltd, and Meryer (Shanghai) Chemical Technology Co Ltd et al.. These reagents and solvents were used as received unless otherwise indicated.

Measurements. ^{1}H NMR and ^{13}C NMR spectra were measured on a Bruker AV 400 spectrometer at 298 K in deuterated reagents. Infrared spectra were recorded on Bruker EQUINAX55 spectrometer. Mass spectrum was measured on an Ion Spec 4.7 Tesla FTMS instrument. Absorption spectra were recorded on a Hewlett Packard 8453 UV–Vis spectrophotometer. Fluorescent spectra were collected on a Shimadzu RF-5301 fluorophotometer at 298 K. The single crystal data was collected on Rigaku Saturn diffractometer with CCD area detector. All calculations were performed using the SHELXL97 and crystal structure crystallographic software packages. The fluorescence quantum yield was measured by Edinburgh F55500w. Circular dichroism (CD) spectra were recorded on a JASCO J-810 spectrometer. Circular polarized luminescence (CPL) spectra were measured on a JASCO CPL200 spectrometer.

The single crystal growth of 6·8PF$_6^-$. **6**·8PF$_6^-$ (3.3 mg, 1.0 mmol) were dissolved in MeCN (1.0 mL), and the solution was passed through a 0.45-μm filter. The bottle was capped. After slowly diffusing diethyl ether into acetonitrile over a period of 3 days, pale-yellow single crystal of **6**·8PF$_6^-$ were obtained.

The single crystal growth of racemic 7·8PF$_6^-$. **7**·8PF$_6^-$ (3.1 mg, 1.0 mmol) was dissolved in a mixture of MeCN/1,4-Dioxane (1/1, v/v, 1.0 mL), and the solution was passed through a 0.45-μm filter. The bottle was capped. After slow evaporation at room temperature for 3 days, pale-yellow single crystal of **7**·8PF$_6^-$ was obtained.

Synthesis of 2. Liquid bromine (3 mL) was slowly added into a solution of **1**[28] (1.0 g, 1.79 mmol) in dichloromethane (150 mL) under ice bath condition. The reaction was stirred 30 minutes at room temperature. Add NaOH solution (2 mol/L, 30 mL) slowly to quench the reaction. The reaction mixture was stirred at room temperature for 30 minutes. Organic phase was separated and water phase was washed three times with dichloromethane. The combined organic phase was dried over anhydrous Na_2SO_4 and concentrated and the residue was crystallized with methyl alcohol to obtain **2** as a white solid (1.52 g, 99%). Mp > 300 °C. ^{1}H NMR (400 MHz, $CDCl_3$) δ 2.30 (s, 12 H), 2.10 (s, 12 H), 1.75 (s, 12 H), 1.71 (s, 12 H). ^{13}C NMR (101 MHz, $CDCl_3$) δ 145.0, 141.7, 136.2, 135.2, 134.0, 133.4, 128.3, 22.5, 21.6, 20.0. IR (KBr) *v* 3466, 3049, 2954, 2914, 1636, 1533, 1455, 1383, 1212, 1126, 1000, 921, 741, 631, 549, 420 cm^{-1}. HR-MS (ESI): m/z calcd for $C_{42}H_{48}Br_4Na^+$ 895.0341 $[M+Na]^+$, found 895.5858 $[M+Na]^+$.

Synthesis of *M*-or *P*-3. A Schlenk tube was charged with **2** (1.5 g, 1.72 mmol), Pyridine-4-boronic acid (2.1 g, 17.2 mmol), K_2CO_3 (4.7 g, 34.4 mmol) and Pd $(PPh_3)_4$ (0.2 g, 0.17 mmol). The mixture was placed under nitrogen atmosphere, degassed 1,4-dioxane (20 mL) and H_2O (5 mL) were added. The reaction was stirred at 100 °C for 48 h, then cooled down to room temperature and the solvents were removed under reduced pressure. The residue was dissolved in dichloromethane (200 mL) and washed successively by water (150 mL).

After drying over Na_2SO_4, the solvent was removed and the residue was purified by column chromatography on silica gel (dichloromethane/methanol = 100/5) to afford **3** as a pale-yellow solid (1.2 g, 82%). The obtained compound **3** was resolved into *M*-**3** and *P*-**3** enantiomers through a chiral HPLC column using CHIRALCEL OD-H (ODH0CD-TC012) column and MeOH / ACN / DEA (50:50:0.1, v/v/v) mobile phase.

Racemic 3. Mp > 300 °C. ^{1}H NMR (400 MHz, $CDCl_3$) δ 8.64 (t, *J* = 4.0 Hz, 8H), 7.11 (s, 4H), 6.92 (s, 4H), 1.87 (s, 12H), 1.82 (s, 12H), 1.80 (s, 12H), 1.62 (s, 12H). ^{13}C NMR (101 MHz, $CDCl_3$) δ 151.7, 149.8, 149.7, 145.6, 142.5, 138.1, 135.5, 134.1, 130.9, 130.1, 125.1, 124.8, 21.6, 18.5, 18.5, 18.4. IR (KBr) *v* 3467, 3041, 3013, 2919, 2733, 1634, 1596, 1538, 1460, 1404, 1265, 1214, 1006, 820, 738, 641, 540, 420 cm^{-1}. HR-MS (ESI): m/z calcd for $C_{62}H_{64}N_4H^+$ 865.5204 $[M+H]^+$, found 865.5275 $[M+H]^+$.

M-**3**. $[\alpha]^{25}_D$ = +956° (THF, c = 3 mg/mL), Mp > 300 °C. ^{1}H NMR (400 MHz, $CDCl_3$) δ 8.64 (t, *J* = 4.0 Hz, 8H), 7.11 (s, 4H), 6.92 (s, 4H), 1.87 (s, 12H), 1.82 (s, 12H), 1.80 (s, 12H), 1.62 (s, 12H). ^{13}C NMR (101 MHz, $CDCl_3$) δ 151.7, 149.8, 149.7, 145.6, 142.5, 138.1, 135.5, 134.1, 130.9, 130.1, 125.1, 124.8, 21.6, 18.5, 18.5, 18.4. IR (KBr) *v* 3434, 3062, 3022, 2916, 2728, 1634, 1596, 1540, 1460, 1406, 1214, 1069, 816, 792, 641, 549, 419 cm^{-1}. HR-MS (ESI): m/z calcd for $C_{62}H_{64}N_4H^+$ 865.5204 $[M+H]^+$, found 865.5231 $[M+H]^+$.

P-**3**. $[\alpha]^{25}_D$ = −978° (THF, c = 3 mg/mL), Mp > 300 °C. ^{1}H NMR (400 MHz, $CDCl_3$) δ 8.64 (t, *J* = 4.0 Hz, 8H), 7.11 (s, 4H), 6.92 (s, 4H), 1.87 (s, 12H), 1.82 (s, 12H), 1.80 (s, 12H), 1.62 (s, 12H). ^{13}C NMR (101 MHz, $CDCl_3$) δ 151.7, 149.8, 149.7, 145.6, 142.5, 138.1, 135.5, 134.1, 130.9, 130.1, 125.1, 124.8, 21.6, 18.5, 18.5, 18.4. IR (KBr) *v* 3464,

3062, 3022, 2916, 2728, 1634, 1596, 1540, 1460, 1405, 1272, 1214, 1031, 816, 792, 641, 549, 420 cm^{-1}. HR-MS (ESI): m/z calcd for $C_{62}H_{64}N_4H^+$ 865.5204 $[M+H]^+$, found 865.5228 $[M+H]^+$.

Synthesis of *M*/*P*-4·4PF$_6^-$. **4**·4PF$_6^-$: 1,4-Bis(bromomethyl)benzene (3.0 g, 11.36 mmol) was added to dry MeCN (150 mL) in two-necked flask and the suspension was heated to 90°C until all compound was dissolved. **3** (500 mg, 0.57 mmol) was added to the solution of 1,4-Bis(bromomethyl)benzene in batches and heated to 90°C for another 24 h. Then the mixture was cooled to room temperature, and the precipitate was collected and washed with an excess amount of acetone (3 × 40 mL) by centrifuge to give crude product with Br^- counterions as a yellow solid. Crude product with excess amount of NH_4PF_6 in H_2O (3 × 20 mL) was dissolved and stirred for 12 h. The precipitate was purified by column chromatography on silica gel chromatography (dichloromethane/methanol = 5/1, saturated NH_4PF_6) to afford **4**·4PF$_6^-$ as a yellow solid (1.2 g, 95%). Mp > 300 °C. ^{1}H NMR (400 MHz, CD_3CN) δ 8.74 (d, *J* = 6.4 Hz, 4 H), 8.71 (d, *J* = 6.4 Hz, 4 H), 7.77 (d, *J* = 6.4 Hz, 4 H), 7.65 (d, *J* = 6.4 Hz, 4 H), 7.55 (d, *J* = 8.0 Hz, 8 H), 7.49 (d, *J* = 8.0 Hz, 8 H), 5.73 (s, 8 H), 4.63 (s, 8 H), 1.88 (s, 12 H), 1.85 (s, 12 H), 1.78 (s, 12 H), 1.60 (s, 12 H). ^{13}C NMR (101 MHz, CD_3CN) δ 162.1, 146.2, 144.9, 144.8, 144.1, 140.8, 136.7, 136.1, 135.2, 133.5, 131.4, 130.7, 130.4, 130.3, 64.11, 33.2, 21.7, 18.6, 18.5, 18.3. IR (KBr) *v* 3435, 3125, 3039, 2918, 1637, 1558, 1512, 1459, 1405, 1216, 1180, 1123, 1054, 1003, 954, 842, 557, 487 cm^{-1}. HR-MS (ESI): m/z calcd for $C_{96}H_{96}Br_4F_{12}N_4P_2Na^{3+}$ 646.1175 $[M-2PF_6^-+Na^+]^{3+}$, found 646.1603 $[M-2PF_6^-+Na^+]^{3+}$.

M-**4**·$4PF_6^-$. By the similar condition of **4**·$4PF_6^-$. 1,4-Bis(bromomethyl)benzene (1.0 g, 3.79 mmol), *M*-**3** (0.1 g, 0.12 mmol) and MeCN (50 mL) were used to afford *M*-**4**·$4PF_6^-$ as a yellow solid (0.24 g, 95%). $[\alpha]^{25}_D$ = −512° (MeCN, c = 3 mg/mL), Mp > 300 °C. ^{1}H NMR (400 MHz, CD_3CN) δ 8.74 (d, *J* = 6.4 Hz, 4 H), 8.71 (d, *J* = 6.4 Hz, 4 H), 7.77 (d, *J* = 6.4 Hz, 4 H), 7.65 (d, *J* = 6.4 Hz, 4 H), 7.55 (d, *J* = 8.0 Hz, 8 H), 7.49 (d, *J* = 8.0 Hz, 8 H), 5.73 (s, 8 H), 4.63 (s, 8 H), 1.88 (s, 12 H), 1.85 (s, 12 H), 1.78 (s, 12 H), 1.60 (s, 12 H). ^{13}C NMR (101 MHz, CD_3CN) δ 162.1, 146.2, 144.9, 144.8, 144.1, 140.8, 136.7, 136.1, 135.2, 133.5, 131.4, 130.7, 130.4, 64.1, 33.2, 21.7, 18.6, 18.5, 18.3. IR (KBr) *v* 3435, 3127, 2917, 1637, 1558, 1512, 1459, 1405, 1217, 1149, 1055, 1004, 954, 840, 557 cm^{-1}. HR-MS (ESI): m/z calcd for $C_{96}H_{96}Br_4F_{12}N_4P_2Na^{3+}$ 646.1175 $[M-2PF_6^-+Na^+]^{3+}$, found 646.1329 $[M-2PF_6^-+Na^+]^{3+}$.

P-**4**·$4PF_6^-$. By the similar condition of **4**·$4PF_6^-$. 1,4-Bis(bromomethyl)benzene (1.0 g, 3.79 mmol), *P*-**3** (0.1 g, 0.12 mmol) and MeCN (50 mL) were used to afford *P*-**4**·$4PF_6^-$ as a yellow solid (0.24 g, 95%). $[\alpha]^{25}_D$ = +490° (MeCN, c = 3 mg/mL), Mp > 300 °C. ^{1}H NMR (400 MHz, CD_3CN) δ 8.74 (d, *J* = 6.4 Hz, 4 H), 8.71 (d, *J* = 6.4 Hz, 4 H), 7.77 (d, *J* = 6.4 Hz, 4 H), 7.65 (d, *J* = 6.4 Hz, 4 H), 7.55 (d, *J* = 8.0 Hz, 8 H), 7.49 (d, *J* = 8.0 Hz, 8 H), 5.73 (s, 8 H), 4.63 (s, 8 H), 1.88 (s, 12 H), 1.85 (s, 12 H), 1.78 (s, 12 H), 1.60 (s, 12 H). ^{13}C NMR (101 MHz, CD_3CN) δ 162.1, 146.2, 144.9, 144.8, 144.2, 140.8, 136.7, 136.1, 135.2, 133.5, 131.4, 130.7, 130.4, 64.1, 33.2, 21.7, 18.6, 18.5, 18.3. IR (KBr) *v* 3435, 3128, 2920, 1638, 1559, 1512, 1459, 1405, 1217, 1179, 1149, 1054, 1003, 954, 841, 557 cm^{-1}. HR-MS (ESI): m/z calcd for $C_{96}H_{96}Br_4F_{12}N_4P_2Na^{3+}$ 646.1175 $[M-2PF_6^-+Na^+]^{3+}$, found 646.1406 $[M-2PF_6^-+Na^+]^{3+}$.

Synthesis of *MM*/*PP*-6·8PF$_6^-$. **6**·8PF$_6^-$. **4**·4PF$_6^-$ (150 mg, 68.7 μmol) and tetrabutylammonium iodide (TBAI, 5.0 mg, 13.7 μmol) were added to dry MeCN (150 mL) in sealed tube and the mixture was heated at 90°C. **3** (59.3 mg, 68.7 μmol) was added to the mixture of **4**·4PF$_6^-$ in batches and heated to 110°C for another 3 days. Then the mixture was cooled to room temperature, and the mixture was concentrated to give crude product with Br$^-$ counterions as a yellow solid. Crude product with excess amount of NH_4PF_6 in H_2O (10 mL) was dissolved and stirred for 12 h. The precipitate was collected and washed with an excess amount of H_2O (3 × 20 mL) to give crude product with PF$_6^-$ counterions as a yellow solid. The crude product was purified by silica gel chromatography (dichloromethane/acetonitrile = 2/1, saturated NH_4PF_6) to afford **6**·8PF$_6^-$ as a yellow solid (115.3 mg, 51%). Mp > 300 °C. ^{1}H NMR (400 MHz, DMSO) δ 9.51–9.41 (m, 8 H), 9.00 (d, *J* = 6.4 Hz, 2 H), 8.88 (d, *J* = 6.4 Hz, 2 H), 8.74 (d, *J* = 6.4 Hz, 2 H), 8.53 (d, *J* = 6.4 Hz, 2 H), 8.07 (t, *J* = 8.0 Hz, 4 H), 7.93 (t, *J* = 6.4 Hz, 4 H), 7.78 (q, *J* = 8.0 Hz, 8 H), 7.70 (s, 4 H), 7.66 – 7.61 (m, 8 H), 7.20 (s, 2 H), 7.13 (s, 2 H), 6.04 – 5.85 (m, 16 H), 1.94 – 1.85 (m, 36 H), 1.64 (s, 12 H), 1.48 (s, 6 H), 1.40 (s, 6 H), 1.37 (s, 6 H), 1.31 (s, 6 H), 1.24 (s, 6 H), 1.09 (s, 6 H), 0.95 (s, 6 H), 0.88 (s, 6 H). ^{13}C NMR (101 MHz, CD_3CN) δ 162.2, 162.1, 161.9, 161.7, 146.2, 145.5, 144.9, 144.6, 144.4, 144.1, 143.9, 143.8, 143.6, 137.2, 136.9, 136.6, 136.5, 136.3, 136.2, 135.7, 135.6, 135.0, 134.7, 134.4, 131.3, 131.1, 131.0, 130.8, 130.7, 130.6, 130.5, 130.4, 130.3, 130.1, 129.9, 67.9, 64.8, 64.5, 64.3, 25.8, 22.8, 22.5, 21.8, 18.6, 18.5, 18.3, 18.2, 18.1, 18.0, 17.6. IR (KBr) *v* 3435, 3126, 3047, 2919, 1638, 1513, 1460, 1384, 1149, 955, 845, 559, 439 cm^{-1}. HR-MS (ESI): m/z calcd for $C_{156}H_{160}F_{30}N_8P_5^{3+}$ 957.3675 [M−3PF$_6^-$]$^{3+}$, found 957.3486 [M−3PF$_6^-$]$^{3+}$; calcd. for $C_{156}H_{160}F_{12}N_8P_2^{6+}$ 406.0342 [M−6PF$_6^-$]$^{6+}$, found 406.0301 [M−6PF$_6^-$]$^{6+}$.

MM-**6**·8PF_6^-. By the similar condition of **6**·8PF_6^-. *M*-**4**·4PF_6^- (100 mg, 45.8 μmol), *M*-**3** (39.6 mg, 45.8 μmol), TBAI (3.4 mg, 9.2 μmol) and dry MeCN (100 mL) were used to afford *MM*-**6**·8PF_6^- as a yellow solid (75.7 mg, 50%). $[\alpha]^{25}_D$ = +196° (MeCN, c = 3 mg/mL); Mp > 300 °C. ^{1}H NMR (400 MHz, DMSO) δ 9.50 (d, *J* = 6.4 Hz, 4 H), 9.45 (d, *J* = 6.4 Hz, 4 H), 9.00 (d, *J* = 6.4 Hz, 4 H), 8.87 (d, *J* = 6.4 Hz, 4 H), 8.07 (d, *J* = 6.4 Hz, 4 H), 7.93 (d, *J* = 6.4 Hz, 4 H), 7.78 (q, *J* = 8.0 Hz, 16 H), 7.62 (d, *J* = 6.4 Hz, 4 H), 7.20 (d, *J* = 6.4 Hz, 4 H), 6.00 – 5.85 (m, 16 H), 1.87 (s, 12 H), 1.84 (s, 12 H), 1.64 (s, 12 H), 1.40 (s, 12 H), 1.37 (s, 12 H), 1.24 (s, 12 H), 1.09 (s, 12 H), 0.88 (s, 12 H). ^{13}C NMR (101 MHz, CD_3CN) δ 162.1, 161.9, 146.1, 144.8, 144.6, 144.1, 143.7, 143.6, 137.1, 136.9, 136.6, 136.3, 136.3, 135.6, 135.0, 134.4, 131.3, 131.1, 130.7, 130.6, 130.4, 130.3, 130.1, 64.8, 64.6, 22.8, 21.8, 18.5, 18.2, 18.1, 18.0, 17.6. IR (KBr) *v* 3639, 3436, 3126, 3046, 2921, 1638, 1513, 1460, 1384, 1112, 1055, 845, 558 cm^{-1}. HR-MS (ESI): m/z calcd for $C_{156}H_{160}F_{30}N_8P_5^{3+}$ 957.3675 $[M-3PF_6^-]^{3+}$, found 957.3323 $[M-3PF_6^-]^{3+}$; calcd. for $C_{156}H_{160}F_{12}N_8P_2^{6+}$ 406.0342 $[M-6PF_6^-]^{6+}$, found 406.0559 $[M-6PF_6^-]^{6+}$.

PP-**6**·8PF_6^-. By the similar condition of **6**·8PF_6^-. *P*-**4**·4PF_6^- (100 mg, 45.8 μmol), *P*-**3** (39.6 mg, 45.8 μmol), TBAI (3.4 mg, 9.2 μmol) and dry MeCN (100 mL) were used to afford *PP*-**6**·8PF_6^- as a yellow solid (77.2 mg, 51%). $[\alpha]^{25}_D$ = –208°; Mp > 300 °C. ^{1}H NMR (400 MHz, DMSO) δ 9.50 (d, *J* = 6.4 Hz, 4 H), 9.45 (d, *J* = 6.4 Hz, 4 H), 9.00 (d, *J* = 6.4 Hz, 4 H), 8.87 (d, *J* = 6.4 Hz, 4 H), 8.07 (d, *J* = 6.4 Hz, 4 H), 7.93 (d, *J* = 6.4 Hz, 4 H), 7.78 (q, *J* = 8.0 Hz, 16 H), 7.62 (d, *J* = 6.4 Hz, 4 H), 7.20 (d, *J* = 6.4 Hz, 4 H), 6.00 – 5.85 (m, 16 H), 1.87 (s, 12 H), 1.84 (s, 12 H), 1.64 (s, 12 H), 1.40 (s, 12 H), 1.37 (s, 12 H), 1.24 (s, 12 H), 1.09 (s, 12 H), 0.88 (s, 12 H). ^{13}C NMR (101 MHz, CD_3CN) δ 162.1, 161.9, 146.1, 144.8, 144.6, 144.5, 144.1, 143.7, 143.6, 137.1, 136.9, 136.6, 136.4, 136.3, 135.6, 135.0,

134.4, 131.3, 131.1, 130.7, 130.6, 130.5, 130.4, 130.3, 130.1, 64.8, 64.6, 22.8, 21.8, 18.5, 18.2, 18.1, 18.0, 17.6. IR (KBr) *v* 3466, 3125, 2922, 1638, 1513, 1460, 1149, 1004, 845, 559 cm^{-1}. HR-MS (ESI): m/z calcd for $C_{156}H_{160}F_{30}N_8P_5^{3+}$ 957.3675 $[M-3PF_6^-]^{3+}$, found 957.3645 $[M-3PF_6^-]^{3+}$; calcd. for $C_{156}H_{160}F_{12}N_8P_2^{6+}$ 406.0342 $[M-6PF_6^-]^{6+}$, found 406.0339 $[M-6PF_6^-]^{6+}$.

Synthesis of *MM*/*PP*-6·8Cl⁻. **6**·$8Cl^-$: **6**·$8PF_6^-$ (120 mg, 36.3 μmol) was dissolved in MeCN (10 mL), tetrabutylammoium choride (TBACl, 203 mg, 0.73 mmol) was added and the mixture was stirred for 12 h. The precipitate was collected and washed with MeCN (3 × 20 mL) to give **6**·$8Cl^-$ as a yellow solid (80.7 mg, yield: 95%). Mp > 300 °C. ^{1}H NMR (400 MHz, D_2O) δ 9.21–9.18 (m, 8 H), 8.83 (d, *J* = 6.4 Hz, 2H), 8.77 (d, *J* = 6.4 Hz, 2 H), 8.53 (d, *J* = 6.4 Hz, 2 H), 8.42 (d, *J* = 6.4 Hz, 2 H), 7.91 (t, *J* = 8.0 Hz, 4 H), 7.85 (t, *J* = 8.0 Hz, 4 H), 7.70 (s, 8 H), 7.61 (s, 4H), 7.58 (s, 4 H), 7.49 (d, *J* = 6.4 Hz, 4 H), 7.19 (s, 4 H), 5.96 – 5.80 (m, 16 H), 2.02 (s, 6 H), 1.93 – 1.91 (m, 18 H), 1.84 (s, 12 H), 1.67 (s, 6 H), 1.64 (s, 6 H), 1.54 (s, 6 H), 1.50 (s, 6 H), 1.46 (s, 6 H), 1.43 (s, 6 H), 1.39 (s, 6 H), 1.17 (s, 6 H), 1.07 (s, 6 H), 1.00 (s, 6 H). ^{13}C NMR (101 MHz, D_2O) δ 162.2, 162.1, 161.8, 161.5, 161.0, 145.4, 145.2, 145.0, 144.7, 143.6, 143.3, 136.6, 135.7, 135.6, 130.5, 130.3, 130.1, 129.9, 129.6, 129.2, 128.9, 65.6, 64.6, 63.5, 63.4, 22.2, 21.7, 21.2, 18.1, 18.0, 17.7, 17.5, 17.4, 17.2, 16.9, 16.5. IR (KBr) *v* 3436, 3115, 3041, 2918, 1637, 1512, 1458, 1384, 1281, 1214, 1148, 794, 544 cm^{-1}. HR-MS (ESI): m/z calcd for $C_{156}H_{160}Cl_6N_8^{2+}$ 1179.0445 $[M-2Cl^-]^{2+}$, found 1179.0488 $[M-2Cl^-]^{2+}$; calcd. for $C_{156}H_{160}Cl_5N_8^{3+}$ 774.3732 $[M-3Cl^-]^{3+}$, found 774.3996 $[M-3Cl^-]^{3+}$; calcd. for $C_{156}H_{160}Cl_4N_8^{4+}$ 572.0376 $[M-4Cl^-]^{4+}$, found 572.0288 $[M-4Cl^-]^{4+}$.

MM-**6**·8Cl⁻. By the similar condition of **6**·8Cl⁻. *MM*-**6**·$8PF_6^-$ (50.0 mg, 15.1 μmol), TBACl (83.4 mg, 0.30 mmol) and MeCN (5 mL) were used to afford *MM*-**6**·8Cl⁻ as a yellow solid (34.5 mg, yield: 95%). $[\alpha]^{25}_D$ = +418° (H_2O, c = 3 mg/mL); Mp > 300 °C. ^{1}H NMR (400 MHz, D_2O) δ 9.20 (t, *J* = 8.0 Hz, 8 H), 8.82 (d, *J* = 6.4 Hz, 4 H), 8.78 (d, *J* = 6.4 Hz, 4 H), 7.90 (d, *J* = 6.4 Hz, 4 H), 7.84 (d, *J* = 6.4 Hz, 4 H), 7.70 (s, 16 H), 7.49 (d, *J* = 6.4 Hz, 4 H), 7.20 (d, *J* = 6.4 Hz, 4H), 5.91 – 5.80 (m, 16 H), 1.94 (s, 12 H), 1.91 (s, 12 H), 1.83 (s, 12 H), 1.64 (s, 12 H), 1.46 (s, 12 H), 1.43 (s, 12 H), 1.17 (s, 12 H), 1.00 (s, 12 H). ^{13}C NMR (101 MHz, D_2O) δ 161.5, 161.4, 145.3, 144.5, 144.2, 143.6, 143.3, 136.7, 136.6, 136.3, 135.7, 135.6, 135.2, 134.4, 133.8, 131.3, 130.4, 130.1, 129.9, 129.6, 129.2, 128.5, 64.3, 22.2, 21.1, 18.0, 17.7, 17.5, 17.4, 16.9, 16.5. IR (KBr) *v* 3467, 3038, 2918, 1637, 1511, 1458, 1405, 1281, 1148, 954, 879, 794, 546 cm^{-1}. HR-MS (ESI): m/z calcd for $C_{156}H_{160}Cl_6N_8^{2+}$ 1179.0445 $[M-2Cl^-]^{2+}$, found 1179.0852 $[M-2Cl^-]^{2+}$; calcd. for $C_{156}H_{160}Cl_5N_8^{3+}$ 774.3732 $[M-3Cl^-]^{3+}$, found 774.3695 $[M-3Cl^-]^{3+}$; calcd. for $C_{156}H_{160}Cl_4N_8^{4+}$ 572.0376 $[M-4Cl^-]^{4+}$, found 572.0030 $[M-4Cl^-]^{4+}$.

PP-**6**·8Cl⁻. By the similar condition of **6**·8Cl⁻. *PP*-**6**·$8PF_6^-$ (50.0 mg, 15.1 μmol), TBACl (83.4 mg, 0.30 mmol) and MeCN (5 mL) were used to afford *PP*-**6**·8Cl⁻ as a yellow solid (34.8 mg, yield: 96%). $[\alpha]^{25}_D$ = −435° (H_2O, c = 3 mg/mL); Mp > 300 °C. ^{1}H NMR (400 MHz, D_2O) ^{1}H NMR (400 MHz, D_2O) δ 9.20 (t, *J* = 8.0 Hz, 8 H), 8.82 (d, *J* = 6.4 Hz, 4 H), 8.78 (d, *J* = 6.4 Hz, 4 H), 7.90 (d, *J* = 6.4 Hz, 4 H), 7.84 (d, *J* = 6.4 Hz, 4 H), 7.70 (s, 16 H), 7.49 (d, *J* = 6.4 Hz, 4 H), 7.20 (d, *J* = 6.4 Hz, 4H), 5.91 – 5.80 (m, 16 H), 1.94 (s, 12 H), 1.91 (s, 12 H), 1.83 (s, 12 H), 1.64 (s, 12 H), 1.46 (s, 12 H), 1.43 (s, 12 H), 1.17 (s, 12 H), 1.00 (s, 12 H). ^{13}C NMR (101 MHz, D_2O) δ 161.5, 161.4, 145.3, 144.5, 144.2, 143.6,

143.3, 136.7, 136.6, 136.3, 135.7, 135.6, 135.3, 134.5, 133.8, 131.3, 130.4, 130.1, 129.9, 129.6, 129.2, 128.5, 64.3, 22.2, 21.1, 18.0, 17.7, 17.5, 17.4, 16.9, 16.5. IR (KBr) *v* 3467, 3038, 2918, 1637, 1511, 1458, 1405, 1281, 1148, 954, 879, 794, 546 cm^{-1}. HR-MS (ESI): m/z calcd for $C_{156}H_{160}Cl_5N_8^{3+}$ 774.3732 $[M{-}3Cl^-]^{3+}$, found 774.3838 $[M{-}3Cl^-]^{3+}$; calcd. for $C_{156}H_{160}Cl_4N_8^{4+}$ 572.0376 $[M{-}4Cl^-]^{4+}$, found 572.0667 $[M{-}4Cl^-]^{4+}$.

Synthesis of *M*/*P*-7·8PF_6^-. **7**·8PF_6^-. **4**·4PF_6^- (200 mg, 91.6 μmol) and tetrabutylammonium iodide (6.8 mg, 18.3 μmol) were added to dry MeCN (200 mL) in sealed tube and the mixture was heated at 90°C. **5** (58.6 mg, 91.6 μmol) was added to the mixture of **4**·4PF_6^- in batches and heated to 110°C for another 3 days. Then the mixture was cooled to room temperature, and the mixture was concentrated to give crude product with Br^- counterions as a yellow solid. Crude product with excess amount of NH_4PF_6 in H_2O (15 mL) was dissolved and stirred for 12 h. The precipitate was collected and washed with an excess amount of H_2O (3 × 20 mL) to give crude product with PF_6^- counterions as a yellow solid. The crude product was purified by silica gel chromatography (dichloromethane/acetonitrile = 2/1, saturated NH_4PF_6) to afford **7**·8PF_6^- as a yellow solid (79.0 mg, 28%). Mp > 300 °C. ^1H NMR (400 MHz, DMSO) δ 9.48 (d, *J* = 6.4 Hz, 2 H), 9.40 (d, *J* = 6.4 Hz, 2 H), 9.15 – 9.08 (m, 12 H), 8.34 (d, *J* = 6.4 Hz, 8 H), 7.93 (d, *J* = 6.4 Hz, 4 H), 7.88 (d, *J* = 6.4 Hz, 4 H), 7.78 (d, *J* = 6.4 Hz, 2 H), 7.82 - 7.72 (m, 22 H), 7.61 (d, *J* = 8.0 Hz, 2 H), 7.53 (d, *J* = 6.4 Hz, 2 H), 6.96 (s, 4 H), 5.94 - 5.76 (m, 16 H), 1.78 (s, 6 H), 1.76 (s, 6 H), 1.73 (s, 6 H), 1.68 (s, 6 H), 1.60 (s, 6 H), 1.46 (s, 6 H), 1.00 (s, 6 H), 0.79 (s, 6 H). ^{13}C NMR (101 MHz, DMSO) δ 153.9, 153.0, 147.4, 145.5, 144.5, 142.9, 140.5, 137.9, 137.3, 135.9, 135.2, 134.6, 134.2, 133.4, 132.4, 132.2, 131.6, 130.7, 130.7, 130.5, 130.1, 129.4, 127.2, 124.5, 54.5, 22.0, 18.8, 18.5, 18.4, 17.5, 17.2, 16.8. IR (KBr) *v*

3435, 3127, 3043, 2922, 1638, 1603, 1461, 1384, 1298, 1219, 1157, 1028, 846, 558 cm^{-1}. HR-MS (ESI): m/z calcd for $C_{140}H_{128}F_{42}KN_8NaP_7^{3+}$ 999.5769 $[M–PF_6^{-}+K^{+}+Na^{+}]^{3+}$, found 999.5406 $[M–PF_6^{-}+K^{+}+Na^{+}]^{3+}$.

M-**7**·8PF_6^{-}. By the similar condition of **7**·8PF_6^{-}. *M*-**4**·4PF_6^{-} (100 mg, 45.8 μmol), **5** (29.3 mg, 45.8 μmol), TBAI (3.4 mg, 9.2 μmol) and dry MeCN (100 mL) were used to afford *M*-**7**·8PF_6^{-} as a yellow solid (41.1 mg, 29%). $[\alpha]^{25}_{D}$ = +256° (MeCN, c = 3 mg/mL); Mp > 300 °C. ^{1}H NMR (400 MHz, DMSO) δ 9.48 (d, *J* = 6.4 Hz, 2 H), 9.40 (d, *J* = 6.4 Hz, 2 H), 9.15 – 9.08 (m, 12 H), 8.34 (d, *J* = 6.4 Hz, 8 H), 7.93 (d, *J* = 6.4 Hz, 4 H), 7.88 (d, *J* = 6.4 Hz, 4 H), 7.78 (d, *J* = 6.4 Hz, 2 H), 7.82 - 7.72 (m, 22 H), 7.61 (d, *J* = 8.0 Hz, 2 H), 7.53 (d, *J* = 6.4 Hz, 2 H), 6.96 (s, 4 H), 5.94 - 5.76 (m, 16 H), 1.78 (s, 6 H), 1.76 (s, 6 H), 1.73 (s, 6 H), 1.68 (s, 6 H), 1.60 (s, 6 H), 1.46 (s, 6 H), 1.00 (s, 6 H), 0.79 (s, 6 H). ^{13}C NMR (101 MHz, DMSO) δ 154.0, 153.5, 146.1, 145.5, 144.5, 140.5, 137.3, 135.9, 135.2, 134.6, 134.2, 133.4, 132.4, 132.2, 131.6, 130.7, 130.7, 130.5, 130.1, 129.4, 127.2, 124.5, 53.1, 22.0, 18.8, 18.5, 18.4, 17.5, 17.2, 16.8. IR (KBr) *v* 3435, 3127, 3040, 2923, 1638, 1603, 1461, 1406, 1385, 1297, 1221, 1159, 844, 558 cm^{-1}. HR-MS (ESI): m/z calcd for $C_{140}H_{128}F_{42}KN_8NaP_7^{3+}$ 999.5769 $[M–PF_6^{-}+K^{+}+Na^{+}]^{3+}$, found 999.5502 $[M–PF_6^{-}+K^{+}+Na^{+}]^{3+}$.

P-**7**·8PF_6^{-}. By the similar condition of **7**·8PF_6^{-}. *P*-**4**·4PF_6^{-} (100 mg, 45.8 μmol), **5** (29.3 mg, 45.8 μmol), TBAI (3.4 mg, 9.2 μmol) and dry MeCN (100 mL) were used to afford *P*-**7**·8PF_6^{-} as a yellow solid (39.7 mg, 28%). $[\alpha]^{25}_{D}$ = –242° (MeCN, c = 3 mg/mL); Mp > 300 °C. ^{1}H NMR (400 MHz, DMSO) δ 9.48 (d, *J* = 6.4 Hz, 2 H), 9.40 (d, *J* = 6.4 Hz, 2 H), 9.15 – 9.08 (m, 12 H), 8.34 (d, *J* = 6.4 Hz, 8 H), 7.93 (d, *J* = 6.4 Hz, 4 H), 7.88 (d, *J* = 6.4

Hz, 4 H), 7.78 (d, J = 6.4 Hz, 2 H), 7.82 - 7.72 (m, 22 H), 7.61 (d, J = 8.0 Hz, 2 H), 7.53 (d, J = 6.4 Hz, 2 H), 6.96 (s, 4 H), 5.94 - 5.76 (m, 16 H), 1.78 (s, 6 H), 1.76 (s, 6 H), 1.73 (s, 6 H), 1.68 (s, 6 H), 1.60 (s, 6 H), 1.46 (s, 6 H), 1.00 (s, 6 H), 0.79 (s, 6 H). ^{13}C NMR (101 MHz, DMSO) δ 154.0, 153.5, 146.1, 145.5, 144.6, 142.9, 140.5, 137.9, 137.3, 135.9, 135.2, 134.6, 134.2, 133.4, 132.4, 132.2, 131.6, 130.7, 130.7, 130.5, 130.1, 129.4, 127.2, 124.5, 54.5, 22.0, 18.8, 18.5, 18.4, 17.5, 17.2. IR (KBr) v 3436, 3128, 3042, 2923, 1638, 1603, 1461, 1407, 1297, 1220, 1159, 844, 558 cm^{-1}. HR-MS (ESI): m/z calcd for $C_{140}H_{128}F_{42}KN_8NaP_7^{3+}$ 999.5769 [M–PF_6^-+K^++Na^+]$^{3+}$, found 999.5664 [M–PF_6^-+K^++Na^+]$^{3+}$.

Synthesis of *M*/*P*-7·8Cl⁻. **7**·8Cl⁻: By the similar condition of **6**·8Cl⁻. **7**·8PF_6^- (100 mg, 32.4 μmol), TBACl (178 mg, 0.64 mmol) and MeCN (10 mL) were used to afford **7**·8Cl⁻ as a yellow solid (67.7 mg, 96%). Mp > 300 °C. ^{1}H NMR (400 MHz, D_2O) δ 9.15 (d, J = 6.4 Hz, 2 H), 9.08 (d, J = 6.4 Hz, 2 H), 8.86 - 8.82 (m, 10 H), 8.70 (d, J = 6.4 Hz, 2 H), 8.12 (d, J = 6.4 Hz, 4 H), 8.05 (d, J = 6.4 Hz, 4 H), 7.78 (d, J = 6.4 Hz, 2 H), 7.71 - 7.63 (m, 22 H), 7.52 (d, J = 8.0 Hz, 4 H), 7.47 (d, J = 6.4 Hz, 2 H), 7.40 (d, J = 6.4 Hz, 2 H), 7.20 (d, J = 8.0 Hz, 4 H), 7.07 (d, J = 8.0 Hz, 4 H), 5.87 - 5.72 (m, 16 H), 1.84 (s, 6 H), 1.83 (s, 6 H), 1.68 (s, 6 H), 1.65 (s, 6 H), 1.58 (s, 6 H), 1.09 (s, 6 H), 0.83 (s, 6 H). ^{13}C NMR (101 MHz, D_2O) δ 161.4, 155.5, 145.9, 145.3, 143.6, 143.3, 140.9, 136.6, 136.4, 135.5, 135.2, 134.8, 132.7, 132.6, 130.2, 129.94 129.6, 127.7, 127.6, 125.1, 64.5, 63.9, 21.7, 21.1, 17.6, 16.7, 16.6. IR (KBr) v 3434, 3115, 3035, 1635, 1601, 1496, 1459, 1405, 1296, 1212, 1157, 1010, 875, 839, 791, 641, 610, 548 cm^{-1}. HR-MS (ESI): m/z calcd for $C_{140}H_{128}Cl_7KN_8^{2+}$ 1103.8856 [M–Cl^-+K^+]$^{2+}$, found 1103.8838 [M–Cl^-+K^+]$^{2+}$; calcd. for $C_{140}H_{128}Cl_7KN_8Na^{3+}$ 743.5868 [M–Cl^-+K^++Na^+]$^{3+}$, found 743.5755 [M–Cl^-+K^++Na^+]$^{3+}$.

M-**7**·8Cl⁻. By the similar condition of **7**·8Cl⁻. *M*-**7**·$8PF_6^–$ (50 mg, 16.2 μmol), TBACl (88 mg, 0.32 mmol) and MeCN (5 mL) were used to afford *M*-**7**·8Cl⁻ as a yellow solid (34.3 mg, 96%). $[\alpha]^{25}_D$ = +337° (H_2O, c = 3 mg/mL); Mp > 300 °C. ^{1}H NMR (400 MHz, D_2O) δ 9.15 (d, *J* = 6.4 Hz, 2 H), 9.08 (d, *J* = 6.4 Hz, 2 H), 8.86 - 8.82 (m, 10 H), 8.70 (d, *J* = 6.4 Hz, 2 H), 8.12 (d, *J* = 6.4 Hz, 4 H), 8.05 (d, *J* = 6.4 Hz, 4 H), 7.78 (d, *J* = 6.4 Hz, 2 H), 7.71 - 7.63 (m, 22 H), 7.52 (d, *J* = 8.0 Hz, 4 H), 7.47 (d, *J* = 6.4 Hz, 2 H), 7.40 (d, *J* = 6.4 Hz, 2 H), 7.20 (d, *J* = 8.0 Hz, 4 H), 7.07 (d, *J* = 8.0 Hz, 4 H), 5.87 - 5.72 (m, 16 H), 1.84 (s, 6 H), 1.83 (s, 6 H), 1.68 (s, 6 H), 1.65 (s, 6 H), 1.58 (s, 6 H), 1.09 (s, 6 H), 0.83 (s, 6 H). ^{13}C NMR (101 MHz, D_2O) δ 164.5, 155.5, 145.9, 145.3, 143.6, 143.2, 140.4, 136.8, 136.6, 136.4, 135.5, 134.8, 133.4, 132.6, 131.2, 130.2, 129.9, 129.6, 129.2, 127.7, 127.6, 125.1, 124.9, 64.5, 63.9, 21.7, 21.1, 17.6, 16.7, 12.8. IR (KBr) *v* 3436, 3115, 3035, 2918, 1636, 1602, 1497, 1459, 1384, 1296, 1211, 1158, 1011, 791, 611, 548 cm^{-1}. HR-MS (ESI): m/z calcd for $C_{140}H_{128}Cl_7KN_8^{2+}$ 1103.8856 $[M–Cl^–+K^+]^{2+}$, found 1103.8657 $[M–Cl^–+K^+]^{2+}$; calcd. for $C_{140}H_{128}Cl_7KN_8Na^{3+}$ 743.5868 $[M–Cl^–+K^++Na^+]^{3+}$, found 743.5607 $[M–Cl^–+K^++Na^+]^{3+}$.

P-**7**·8Cl⁻. By the similar condition of *M*-**7**·8Cl⁻. *P*-**7**·$8PF_6^–$ (50 mg, 16.2 μmol), TBACl (88 mg, 0.32 mmol) and MeCN (5 mL) were used to afford *P*-**7**·8Cl⁻ as a yellow solid (34.8 mg, 96%). $[\alpha]^{25}_D$ = –323° (H_2O, c = 3 mg/mL); Mp > 300 °C. ^{1}H NMR (400 MHz, D_2O) δ 9.15 (d, *J* = 6.4 Hz, 2 H), 9.08 (d, *J* = 6.4 Hz, 2 H), 8.86 - 8.82 (m, 10 H), 8.70 (d, *J* = 6.4 Hz, 2 H), 8.12 (d, *J* = 6.4 Hz, 4 H), 8.05 (d, *J* = 6.4 Hz, 4 H), 7.78 (d, *J* = 6.4 Hz, 2 H), 7.71 - 7.63 (m, 22 H), 7.52 (d, *J* = 8.0 Hz, 4 H), 7.47 (d, *J* = 6.4 Hz, 2 H), 7.40 (d, *J* = 6.4 Hz, 2 H), 7.20 (d, *J* = 8.0 Hz, 4 H), 7.07 (d, *J* = 8.0 Hz, 4 H), 5.87 - 5.72 (m, 16 H),

1.84 (s, 6 H), 1.83 (s, 6 H), 1.68 (s, 6 H), 1.65 (s, 6 H), 1.58 (s, 6 H), 1.09 (s, 6 H), 0.83 (s, 6 H). ^{13}C NMR (101 MHz, D_2O) δ 162.3, 160.9, 156.2, 155.5, 145.9, 145.6, 145.5, 145.3, 143.6, 143.2, 141.0, 136.4, 135.4, 135.2, 134.8, 133.8, 132.6, 131.2, 130.2, 129.9, 129.6, 129.1, 127.7, 127.6, 125.1, 124.9, 64.7, 63.9, 22.5, 21.1, 17.6, 16.7. IR (KBr) *ν* 3433, 3114, 3035, 1635, 1601, 1497, 1384, 1296, 1211, 1157, 1010, 791, 610, 548 cm^{-1}. HR-MS (ESI): m/z calcd for $C_{140}H_{128}Cl_7KN_8^{2+}$ 1103.8856 [M–Cl$^-$+K$^+$]$^{2+}$, found 1103.8118 [M–Cl$^-$+K$^+$]$^{2+}$; calcd. for $C_{140}H_{128}Cl_7KN_8Na^{3+}$ 743.5868 [M–Cl$^-$+K$^+$+Na$^+$]$^{3+}$, found 743.5457 [M–Cl$^-$+K$^+$+Na$^+$]$^{3+}$.

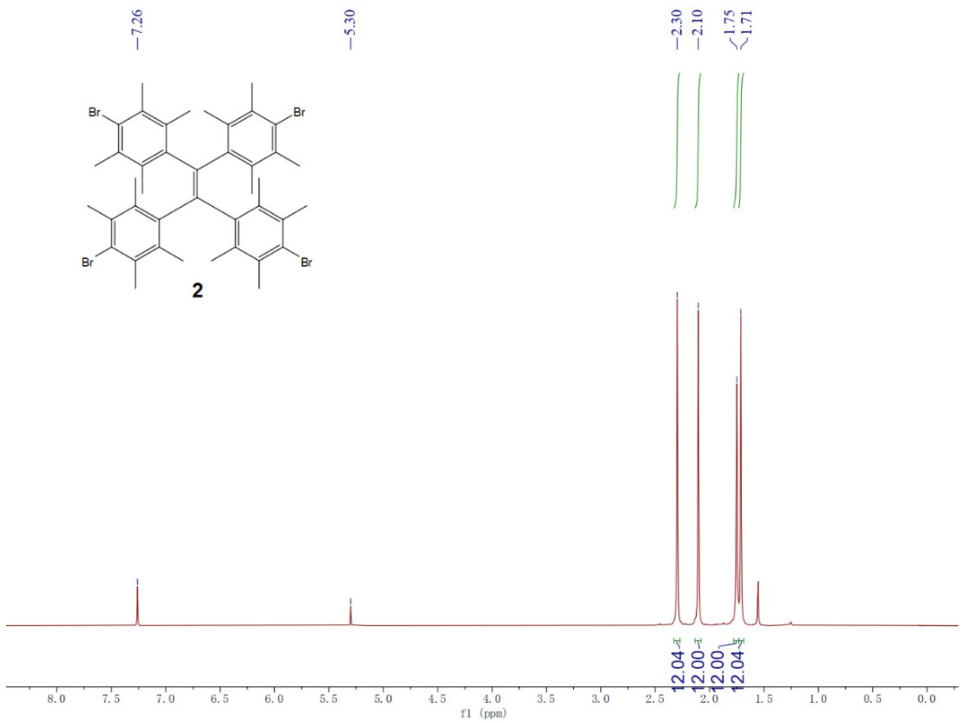

Figure S1. ^{1}H NMR spectrum of compound **2** (400 MHz, $CDCl_3$).

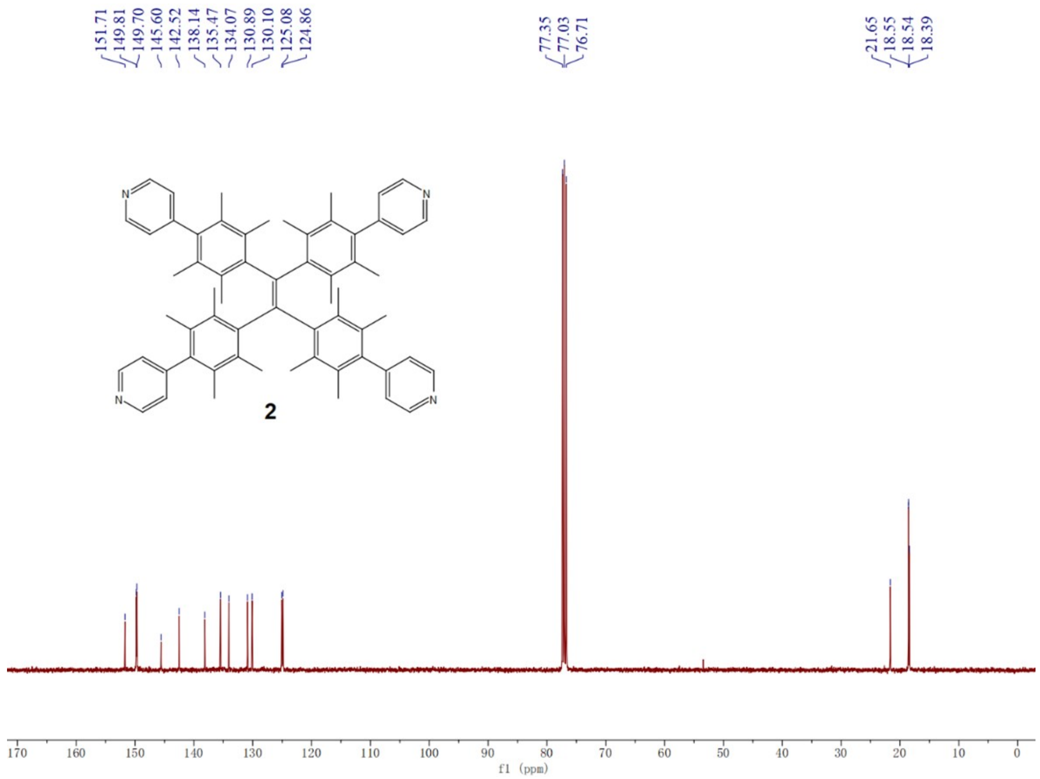

Figure S2. ^{13}C NMR spectrum of compound **2** (101 MHz, $CDCl_3$).

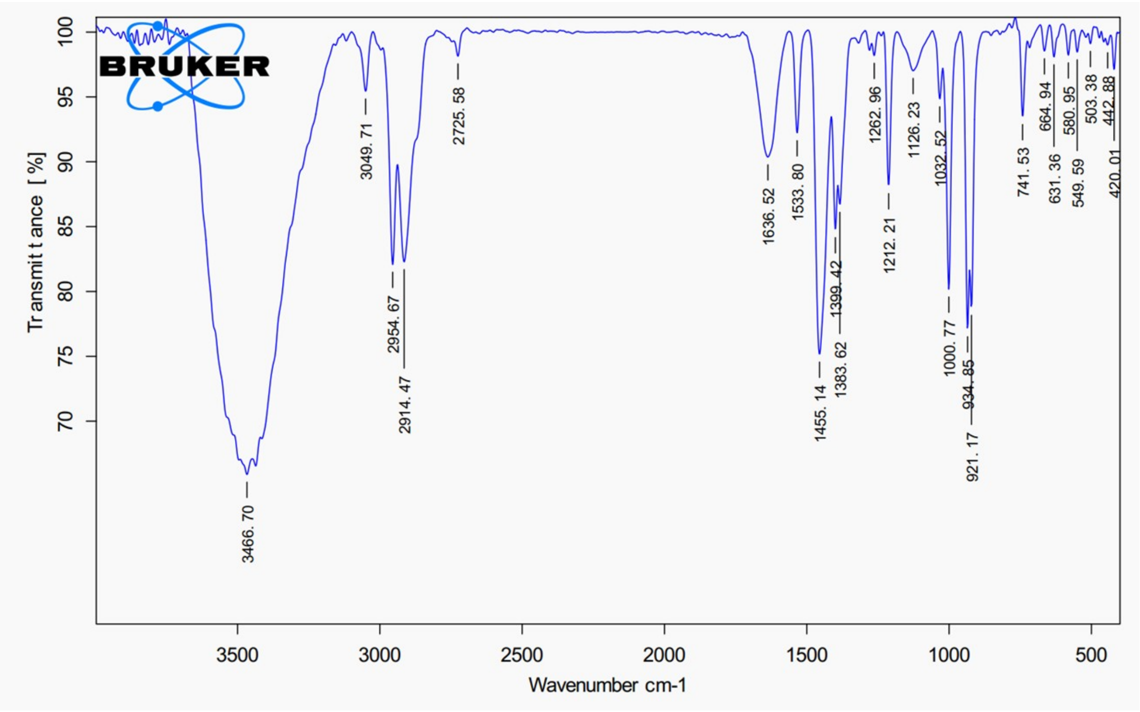

Figure S3. IR spectrum of compound **2**.

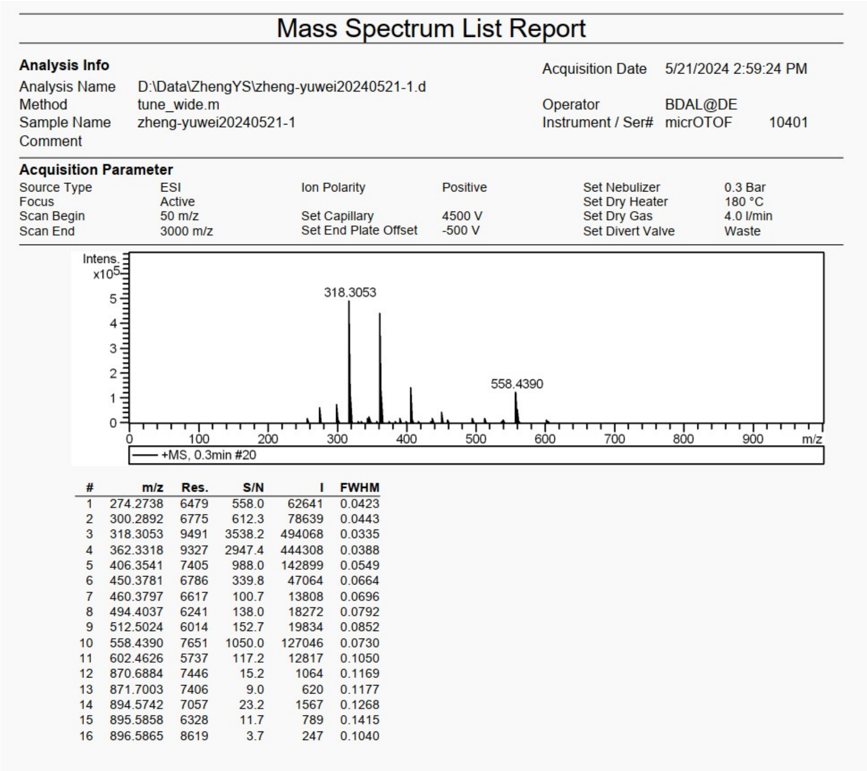

Mass Spectrum List Report

Analysis Info

Analysis Name	D:\Data\ZhengYS\zheng-yuwei20240521-1.d	Acquisition Date	5/21/2024 2:59:24 PM
Method	tune_wide.m	Operator	BDAL@DE
Sample Name	zheng-yuwei20240521-1	Instrument / Ser#	micrOTOF 10401
Comment			

Acquisition Parameter

Source Type	ESI	Ion Polarity	Positive	Set Nebulizer	0.3 Bar
Focus	Active			Set Dry Heater	180 °C
Scan Begin	50 m/z	Set Capillary	4500 V	Set Dry Gas	4.0 l/min
Scan End	3000 m/z	Set End Plate Offset	-500 V	Set Divert Valve	Waste

#	m/z	Res.	S/N	I	FWHM
1	274.2738	6479	558.0	62641	0.0423
2	300.2892	6775	612.3	78639	0.0443
3	318.3053	9491	3538.2	494068	0.0335
4	362.3318	9327	2947.4	444308	0.0388
5	406.3541	7405	988.0	142899	0.0549
6	450.3781	6786	339.8	47064	0.0664
7	460.3797	6617	100.7	13808	0.0696
8	494.4037	6241	138.0	18272	0.0792
9	512.5024	6014	152.7	19834	0.0852
10	558.4390	7651	1050.0	127046	0.0730
11	602.4626	5737	117.2	12817	0.1050
12	870.6884	7446	15.2	1064	0.1169
13	871.7003	7406	9.0	620	0.1177
14	894.5742	7057	23.2	1567	0.1268
15	895.5858	6328	11.7	789	0.1415
16	896.5865	8619	3.7	247	0.1040

Figure S4. HRMS spectrum of compound **2**.

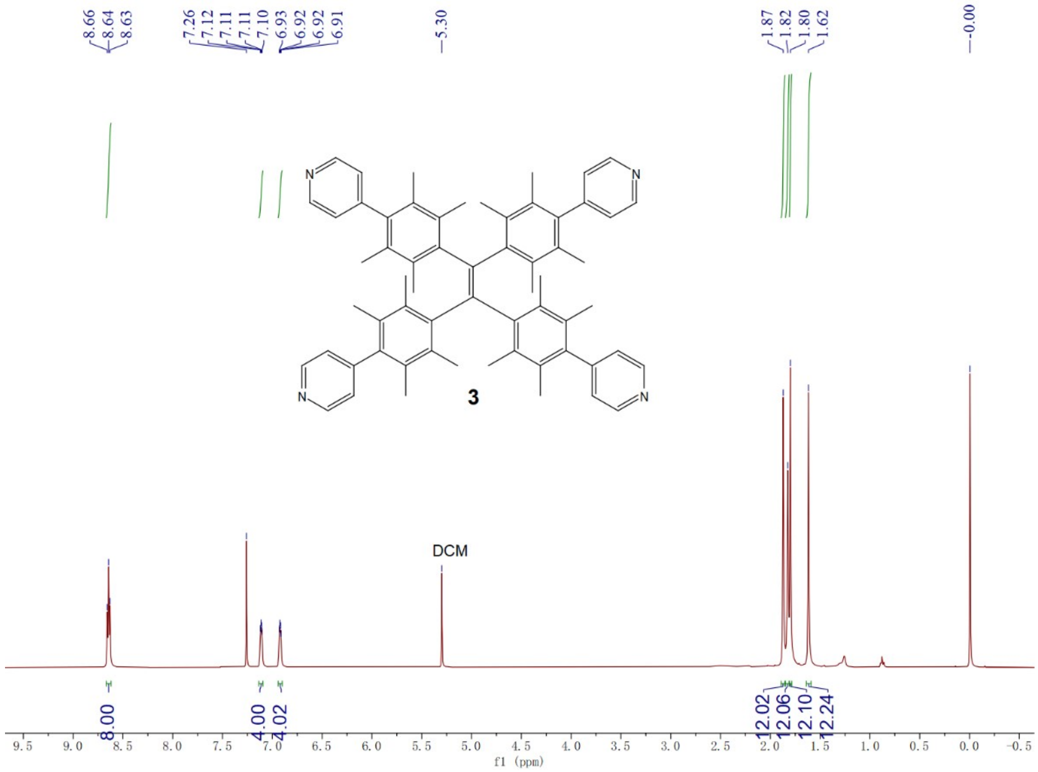

Figure S5. ^{1}H NMR spectrum of compound **3** (400 MHz, $CDCl_3$).

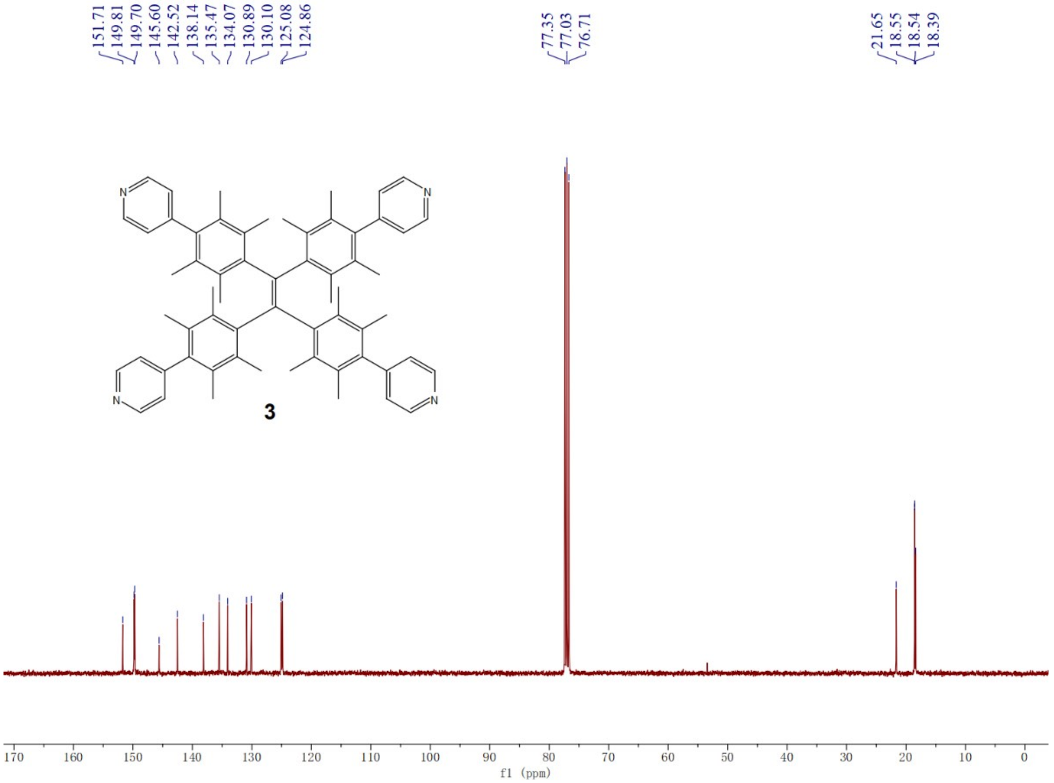

Figure S6. ^{13}C NMR spectrum of compound **3** (101 MHz, $CDCl_3$).

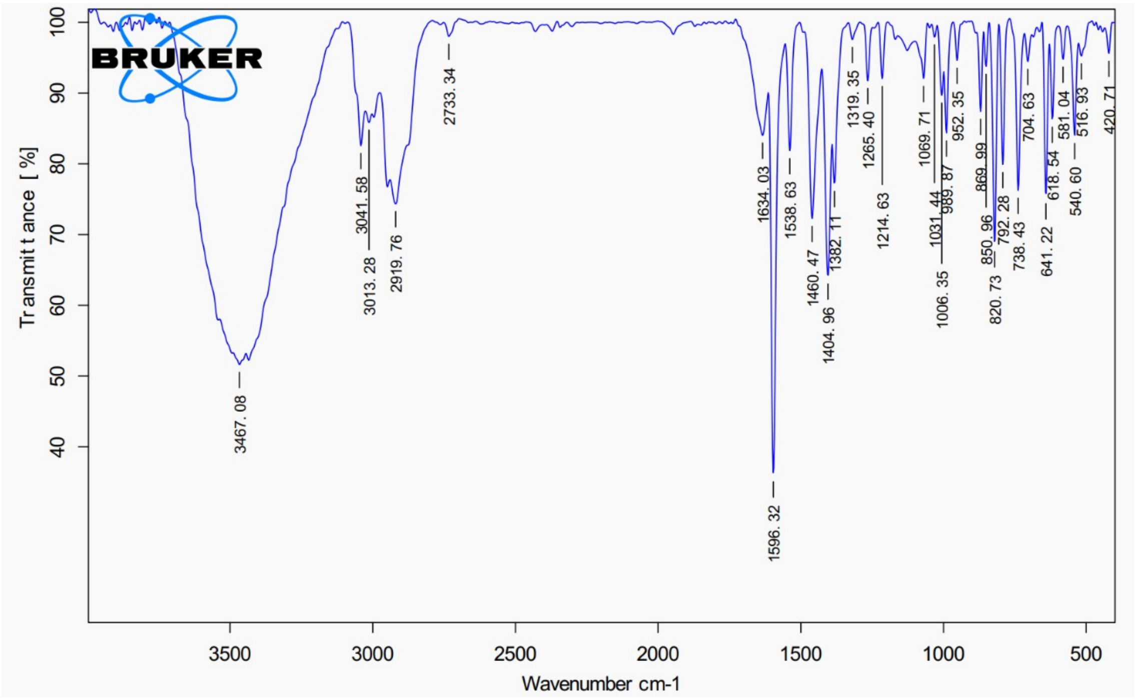

Figure S7. IR spectrum of compound **3**.

Mass Spectrum List Report

Analysis Info

Analysis Name	D:\Data\ZhengYS\zheng-yuwei20240408-2.d	Acquisition Date	4/8/2024 2:41:14 PM
Method	tune_wide.m	Operator	BDAL@DE
Sample Name	zheng-yuwei20240408-2	Instrument / Ser#	micrOTOF 10401
Comment			

Acquisition Parameter

Source Type	ESI	Ion Polarity	Positive	Set Nebulizer	0.3 Bar
Focus	Active			Set Dry Heater	180 °C
Scan Begin	50 m/z	Set Capillary	4500 V	Set Dry Gas	4.0 l/min
Scan End	3000 m/z	Set End Plate Offset	-500 V	Set Divert Valve	Waste

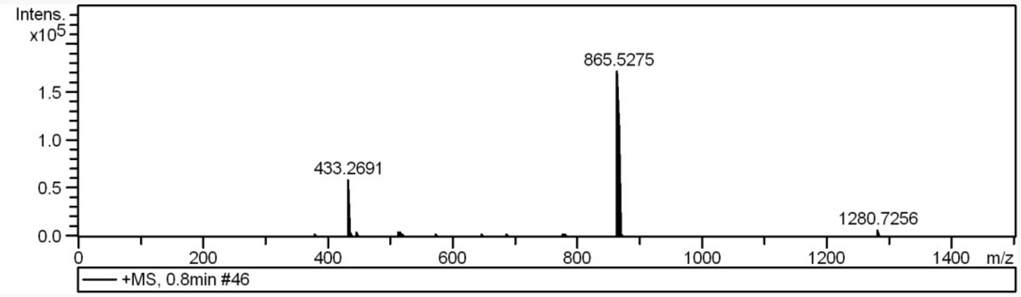

#	m/z	Res.	S/N	I	FWHM
1	433.2691	8577	878.7	60365	0.0505
2	433.7701	8334	646.9	44479	0.0520
3	434.2719	9401	270.0	18584	0.0462
4	865.5275	10582	1747.1	172776	0.0818
5	866.5278	9843	1236.8	122356	0.0880
6	867.5268	8970	475.1	47036	0.0967
7	1280.7256	8555	97.9	6946	0.1497

Figure S8. HRMS spectrum of compound **3**.

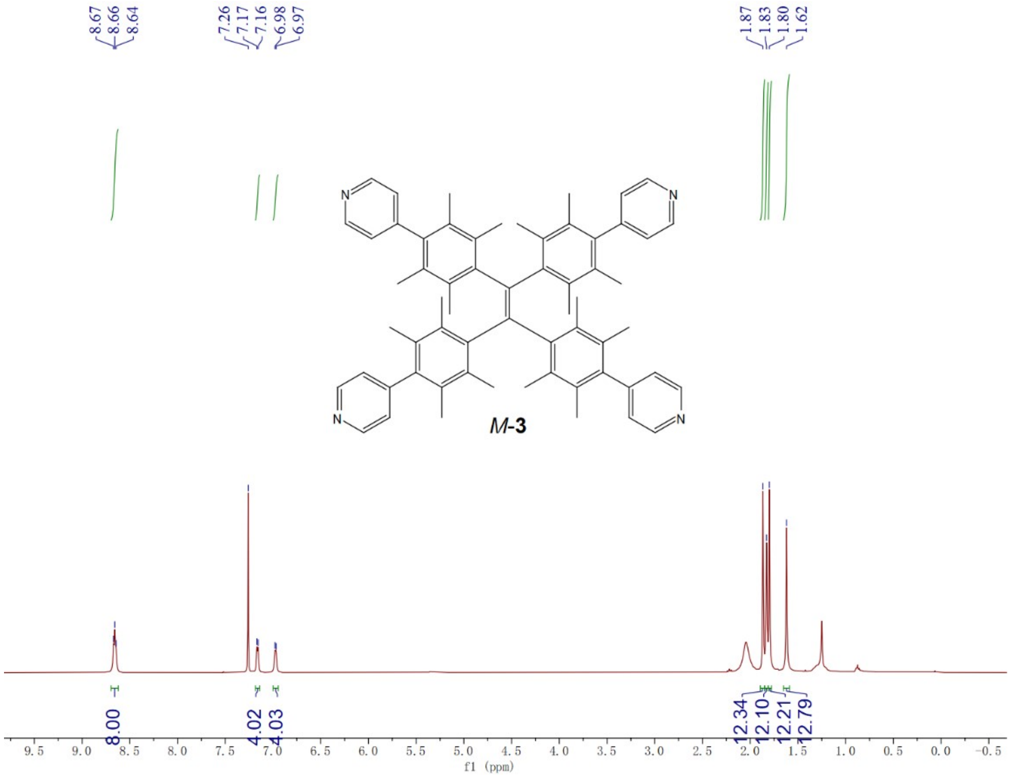

Figure S9. ^{1}H NMR spectrum of compound *M*-**3** (400 MHz, $CDCl_3$).

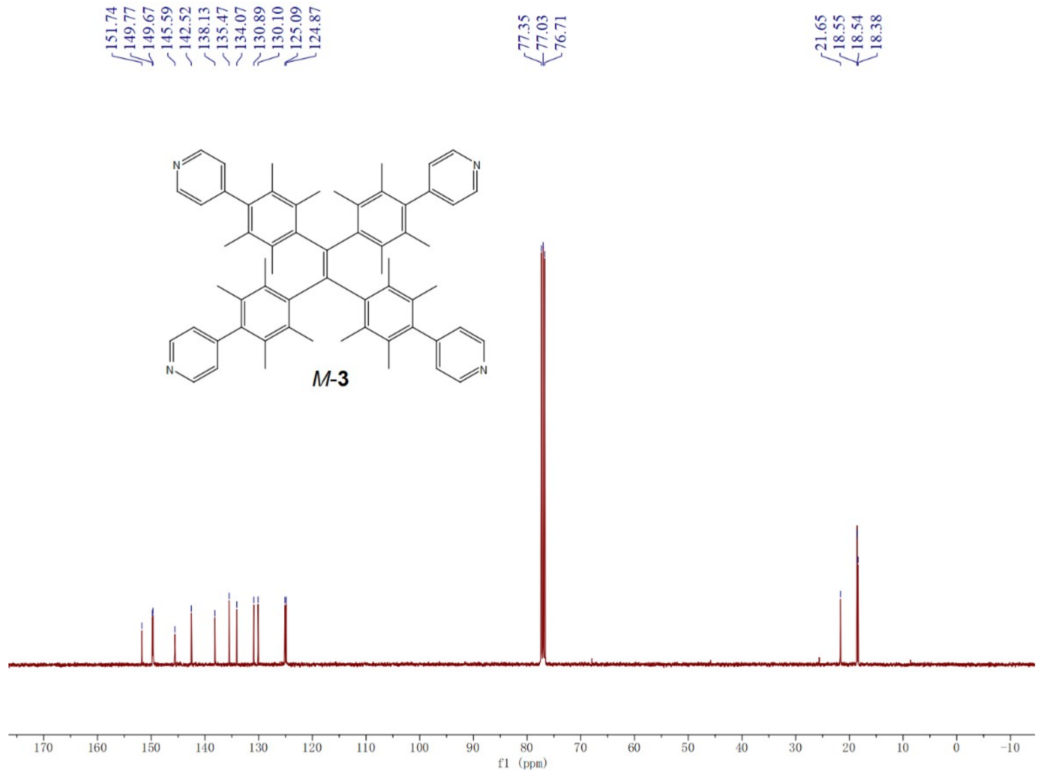

Figure S10. ^{13}C NMR spectrum of compound *M*-**3** (101 MHz, $CDCl_3$).

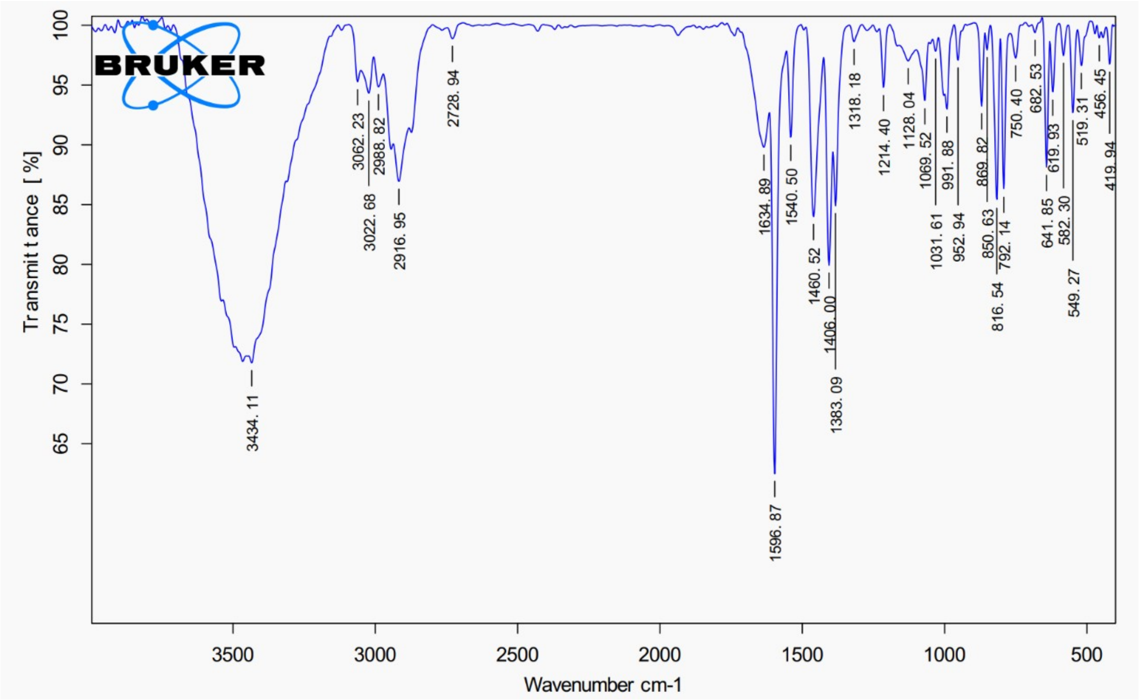

Figure S11. IR spectrum of compound *M*-**3**.

Mass Spectrum List Report

Analysis Info

Analysis Name	D:\Data\ZhengYS\zheng-yuwei20240408-3.d	Acquisition Date	4/8/2024 2:43:42 PM
Method	tune_wide.m	Operator	BDAL@DE
Sample Name	zheng-yuwei20240408-3	Instrument / Ser#	micrOTOF 10401
Comment			

Acquisition Parameter

Source Type	ESI	Ion Polarity	Positive	Set Nebulizer	0.3 Bar
Focus	Active			Set Dry Heater	180 °C
Scan Begin	50 m/z	Set Capillary	4500 V	Set Dry Gas	4.0 l/min
Scan End	3000 m/z	Set End Plate Offset	-500 V	Set Divert Valve	Waste

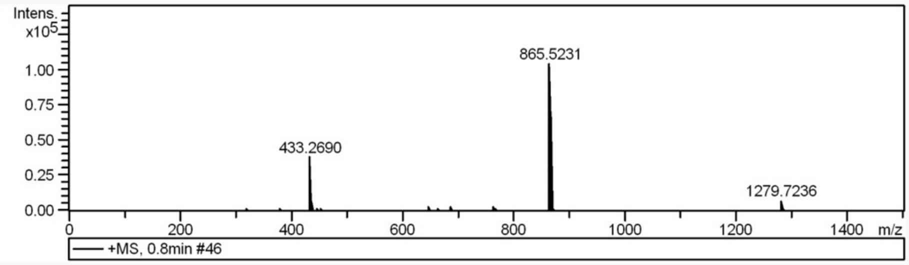

#	m/z	Res.	S/N	I	FWHM
1	433.2690	8140	688.1	38039	0.0532
2	433.7699	8057	472.5	26145	0.0538
3	434.2729	8548	195.6	10832	0.0508
4	865.5231	10079	1321.0	104497	0.0859
5	866.5239	9233	885.4	70090	0.0938
6	867.5270	7444	303.1	24007	0.1165
7	1279.7236	8488	120.0	7430	0.1508

Figure S12. HRMS spectrum of compound *M*-**3**.

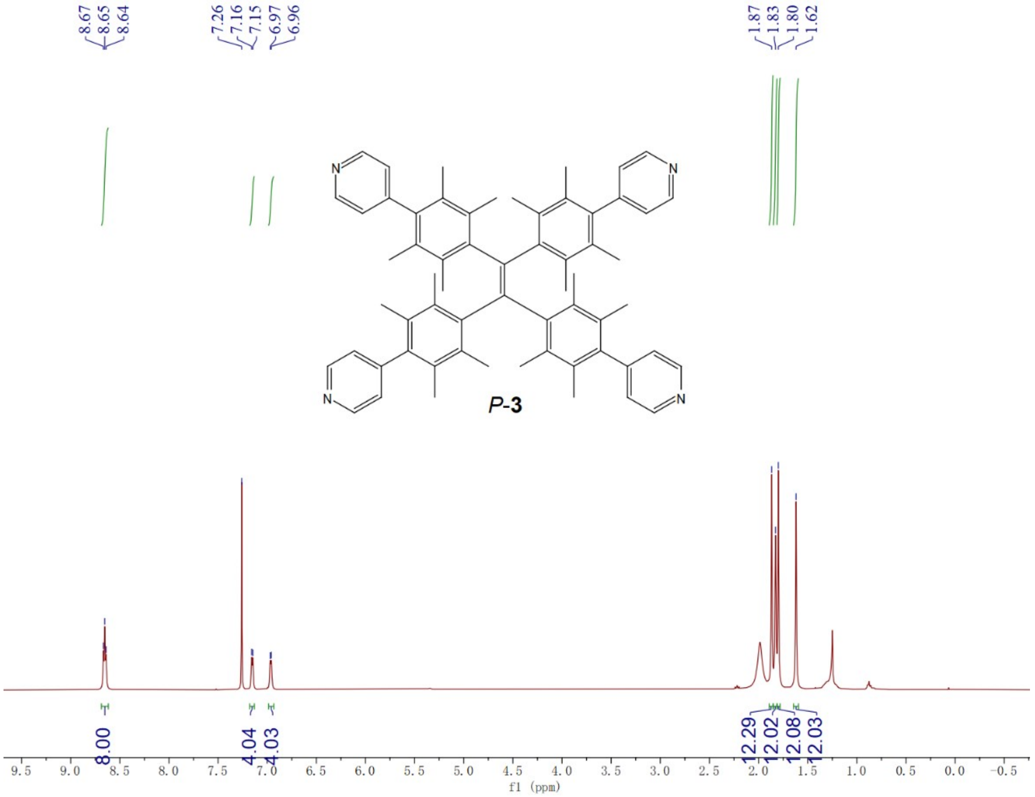

Figure S13. ^{1}H spectrum of compound *P*-**3** (400 MHz, $CDCl_3$).

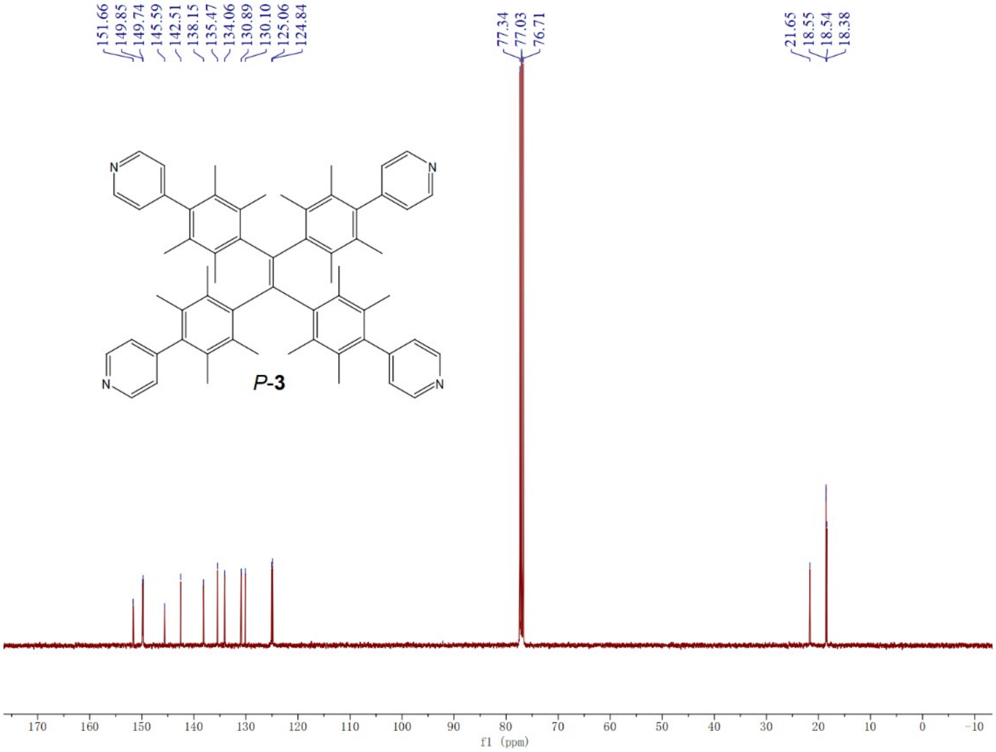

Figure S14. ^{13}C spectrum of compound *P*-**3** (101 MHz, $CDCl_3$).

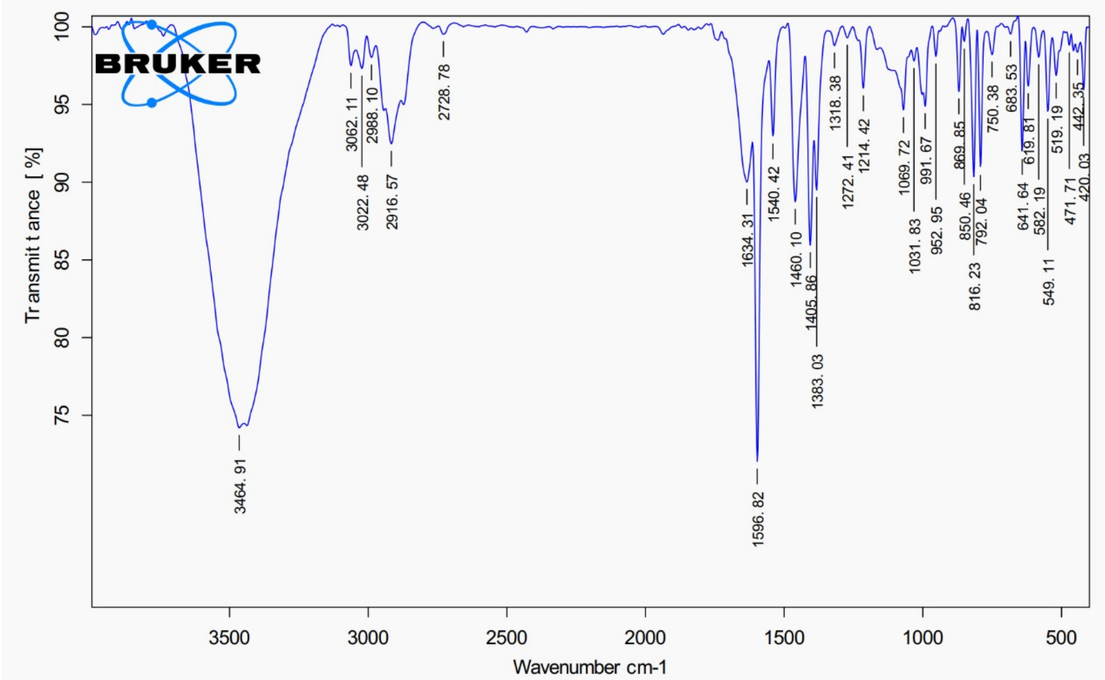

Figure S15. IR spectrum of compound *P*-**3**.

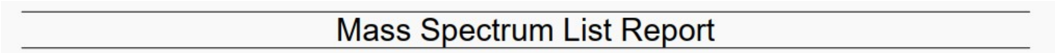

Mass Spectrum List Report

Analysis Info

Analysis Name	D:\Data\ZhengYS\zheng-yuwei20240408-4.d	Acquisition Date	4/8/2024 2:46:17 PM
Method	tune_wide.m	Operator	BDAL@DE
Sample Name	zheng-yuwei20240408-4	Instrument / Ser#	micrOTOF 10401
Comment			

Acquisition Parameter

Source Type	ESI	Ion Polarity	Positive	Set Nebulizer	0.3 Bar
Focus	Active			Set Dry Heater	180 °C
Scan Begin	50 m/z	Set Capillary	4500 V	Set Dry Gas	4.0 l/min
Scan End	3000 m/z	Set End Plate Offset	-500 V	Set Divert Valve	Waste

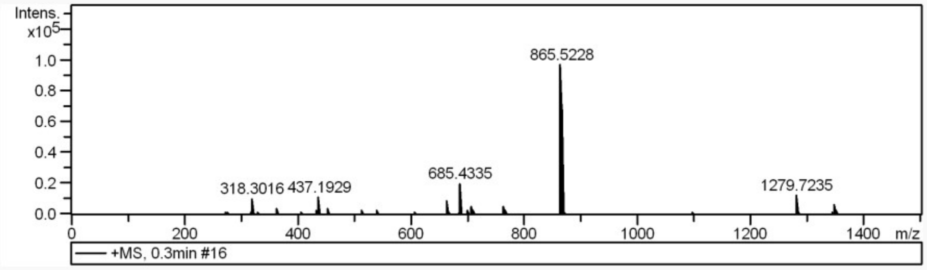

#	m/z	Res.	S/N	I	FWHM
1	318.3016	8272	222.5	10727	0.0385
2	437.1929	8690	197.6	11488	0.0503
3	685.4335	7465	271.5	20477	0.0918
4	865.5228	9517	1131.0	97309	0.0909
5	866.5236	9152	824.9	70991	0.0947
6	867.5274	7289	275.7	23741	0.1190
7	1279.7235	8299	165.9	12226	0.1542

Figure S16. HMRS spectrum of compound *P*-**3**.

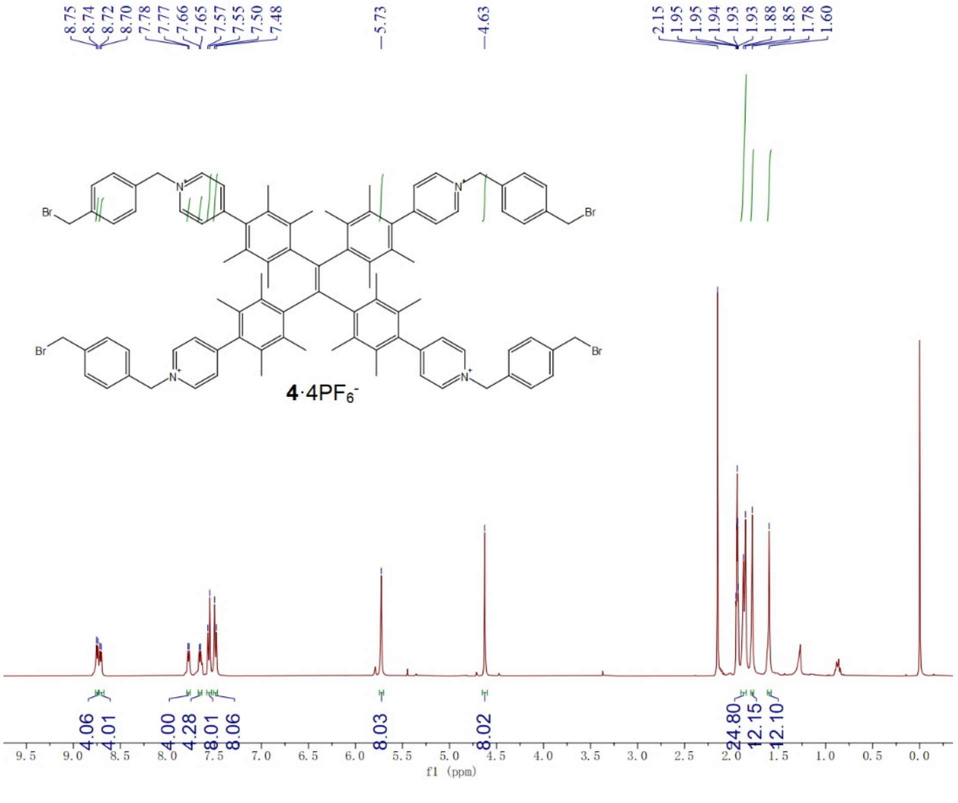

Figure S17. ^{1}H NMR spectrum of compound **4**·4PF_6^- (400 MHz, CD_3CN).

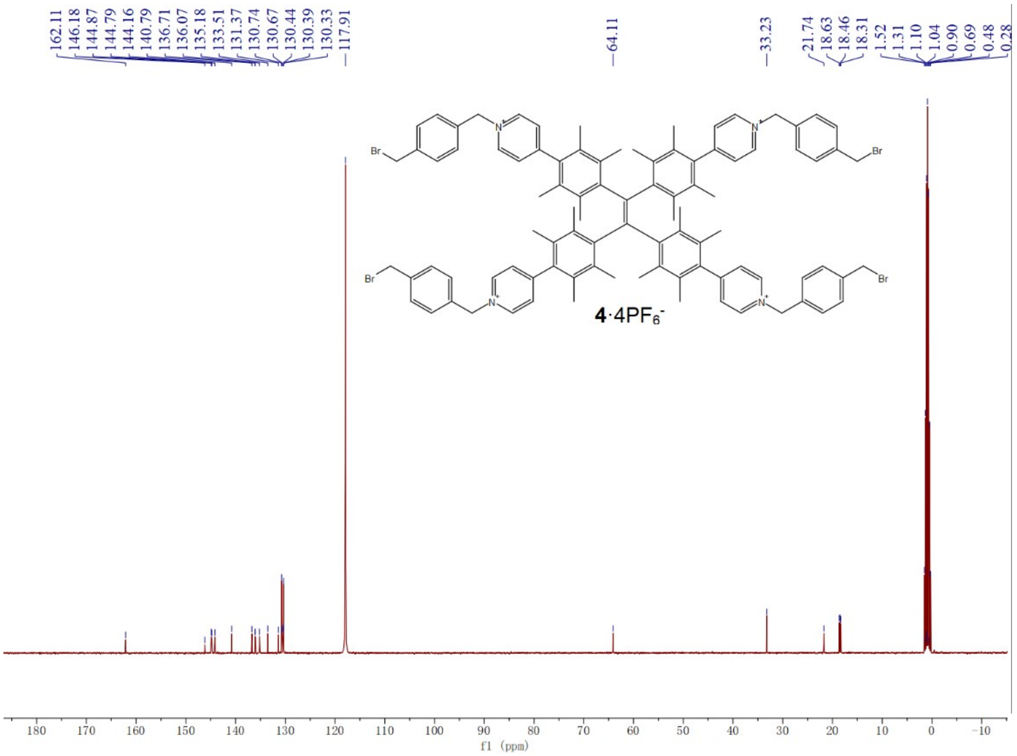

Figure S18. ^{13}C NMR spectrum of compound **4**·4PF_6^- (101 MHz, CD_3CN).

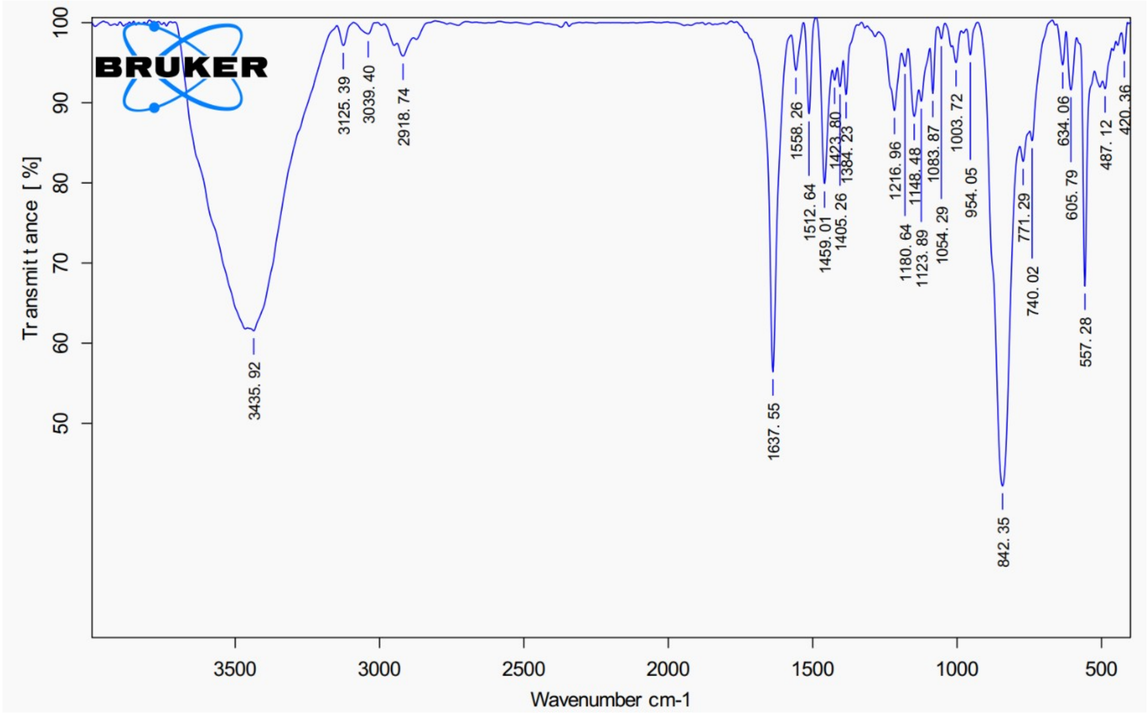

Figure S19. IR spectrum of compound **4**·4PF_6^-.

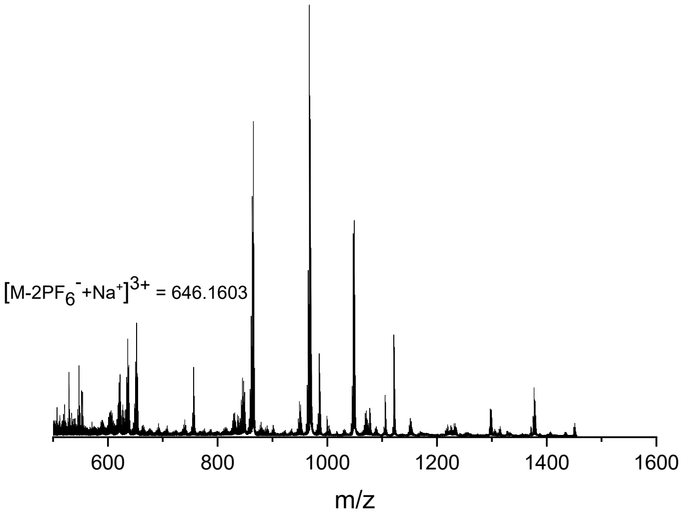

Figure S20. Experimental electrospray ionization mass spectra of **4**·4PF$_6^-$.

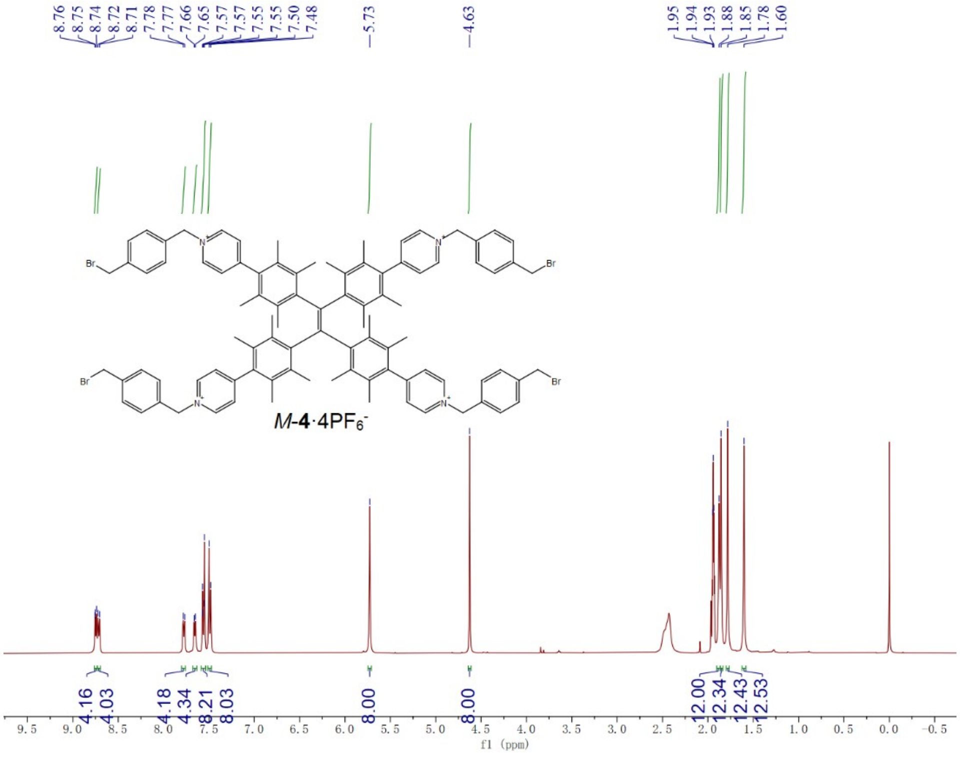

Figure S21. ^{1}H NMR spectrum of compound *M*-**4**·4PF$_6^-$ (400 MHz, CD_3CN).

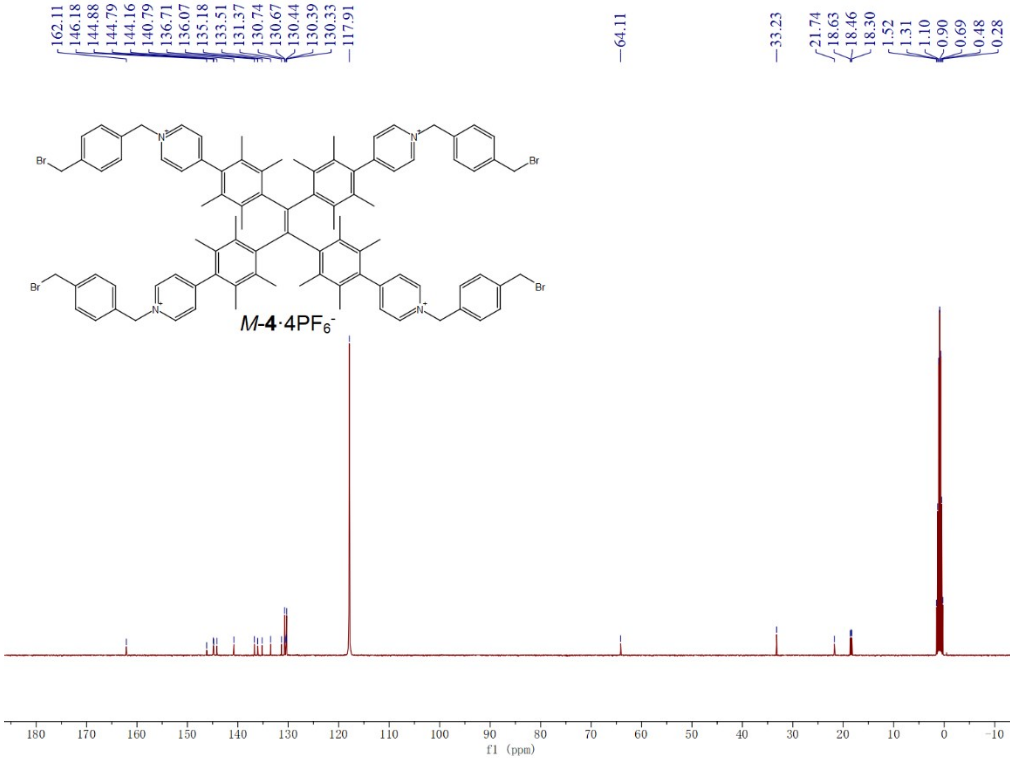

Figure S22. ^{13}C NMR spectrum of compound *M*-**4**·4PF_6^- (101 MHz, CD_3CN).

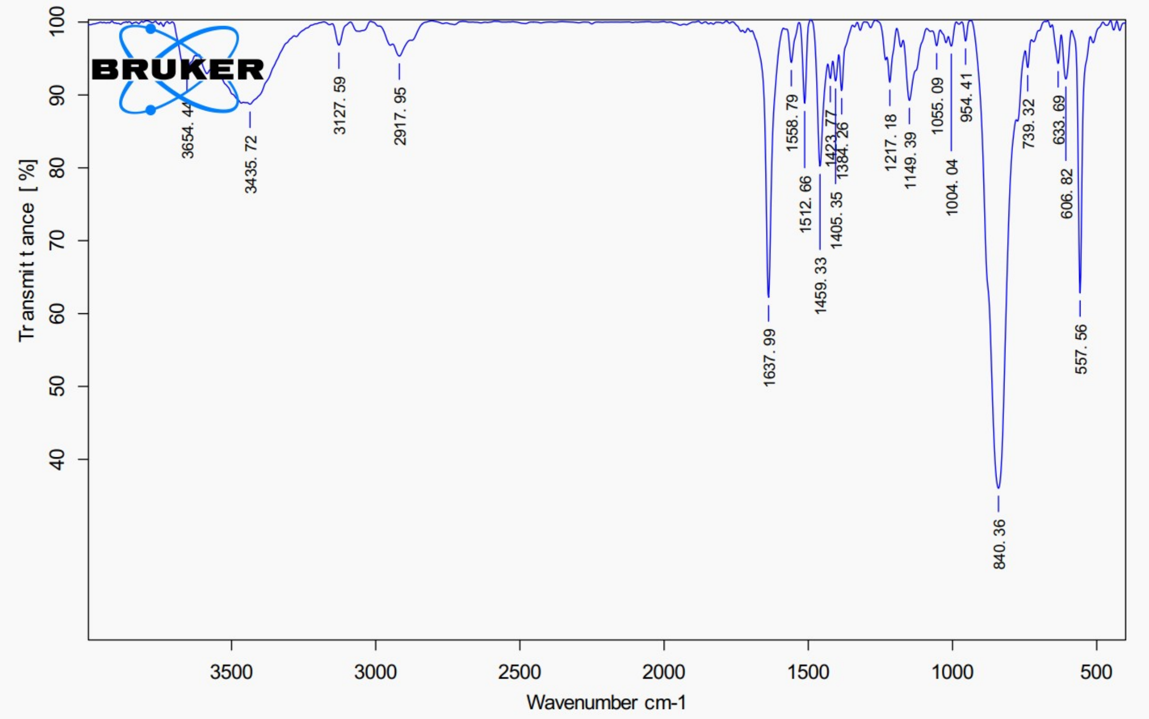

Figure S23. IR spectrum of compound *M*-**4**·4PF_6^-.

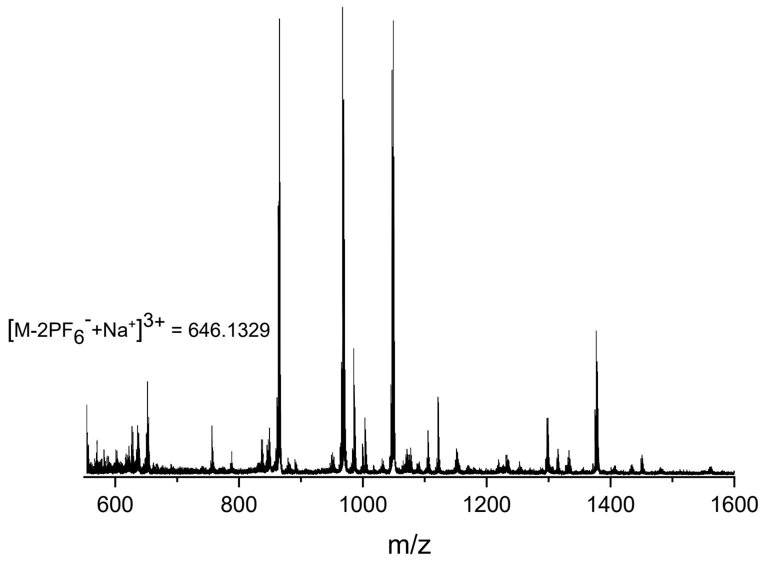

Figure S24. Experimental electrospray ionization mass spectra of *M*-**4**·$4PF_6^-$.

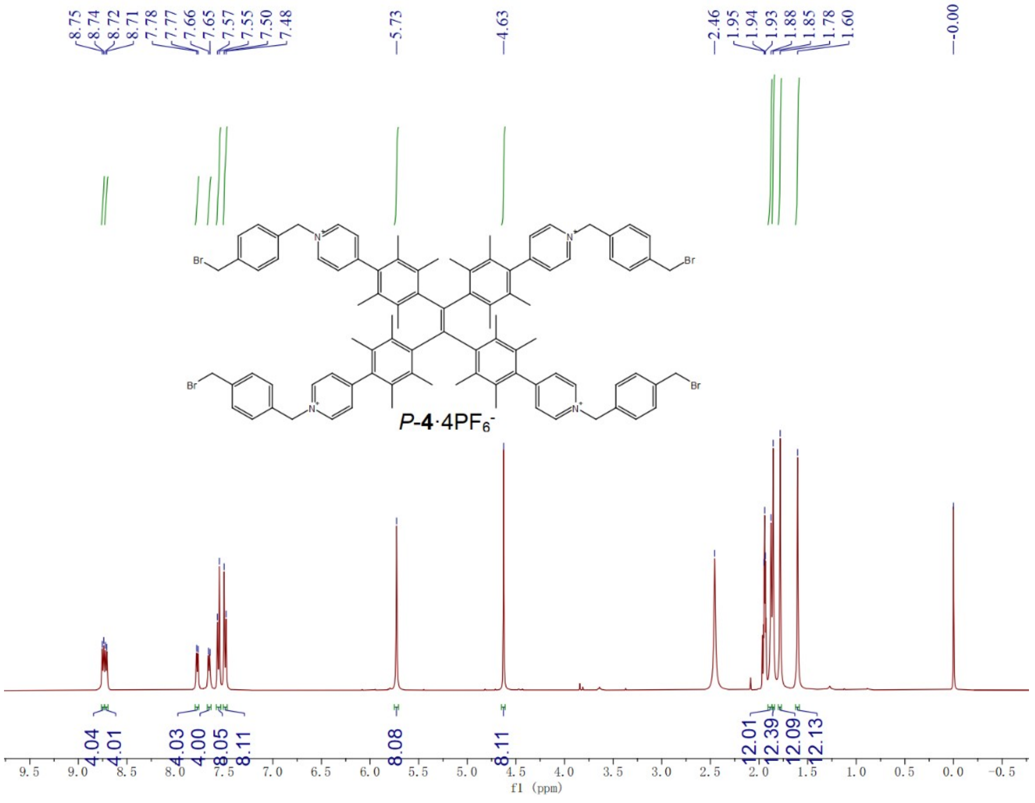

Figure S25. ^{1}H NMR spectrum of compound *P*-**4**·$4PF_6^-$ (400 MHz, CD_3CN).

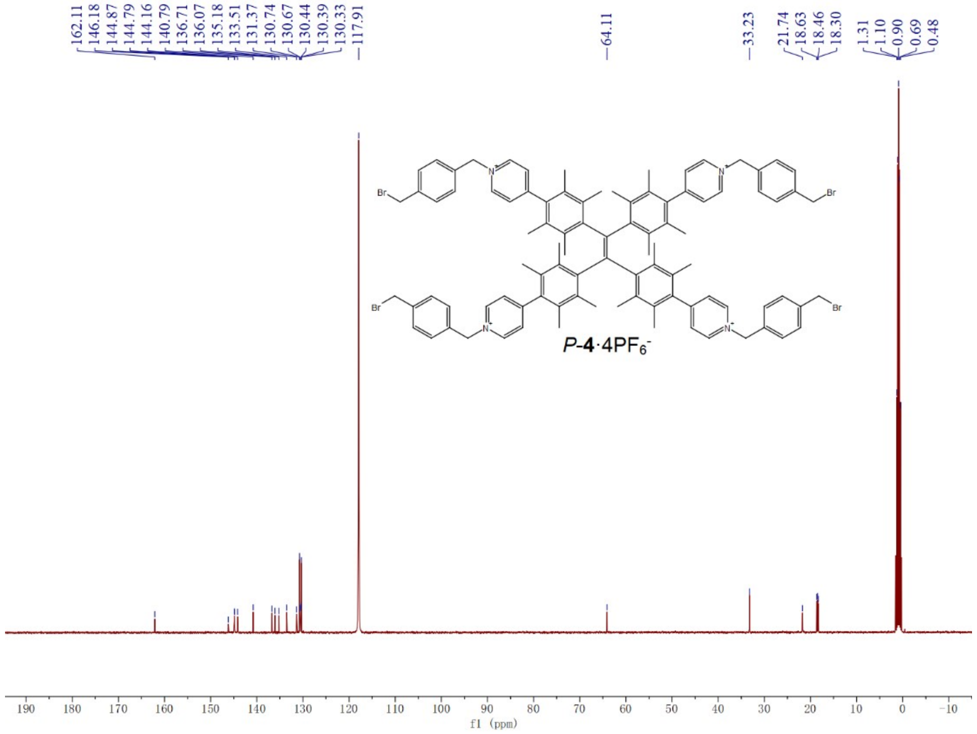

Figure S26. ^{13}C NMR spectrum of compound *P*-**4**·4PF_6^- (101 MHz, CD_3CN).

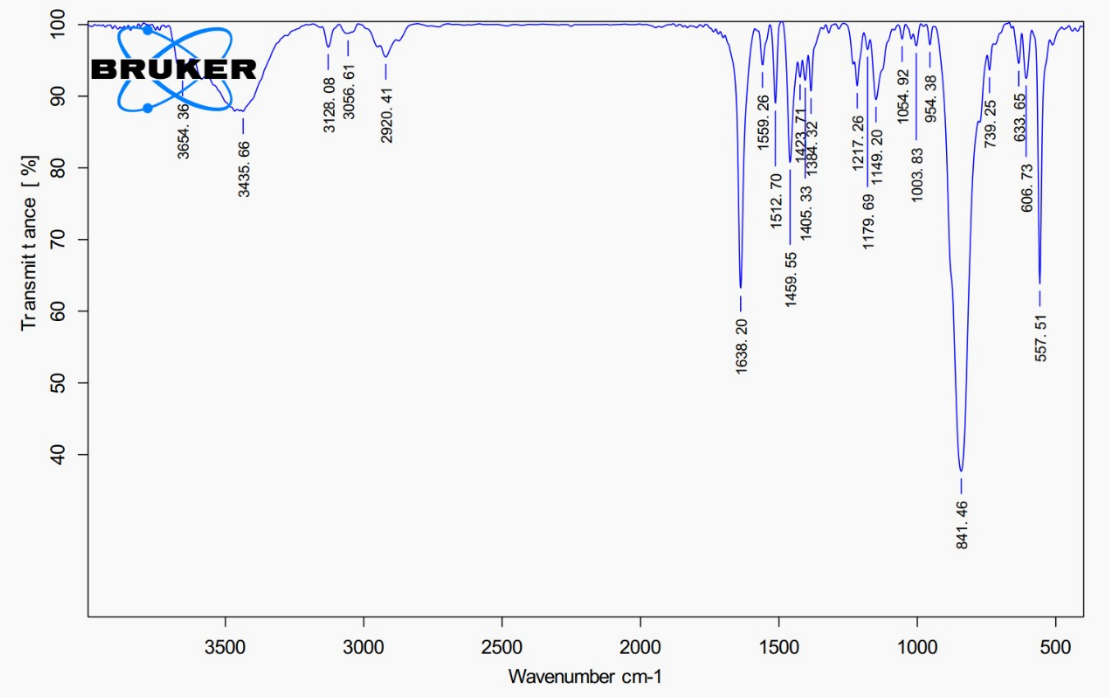

Figure S27. IR spectrum of compound *P*-**4**·4PF_6^-.

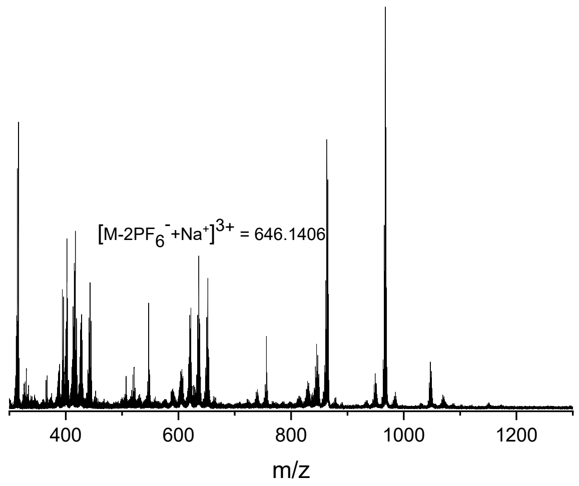

Figure S28. Experimental electrospray ionization mass spectra of *P*-**4**·$4PF_6^-$.

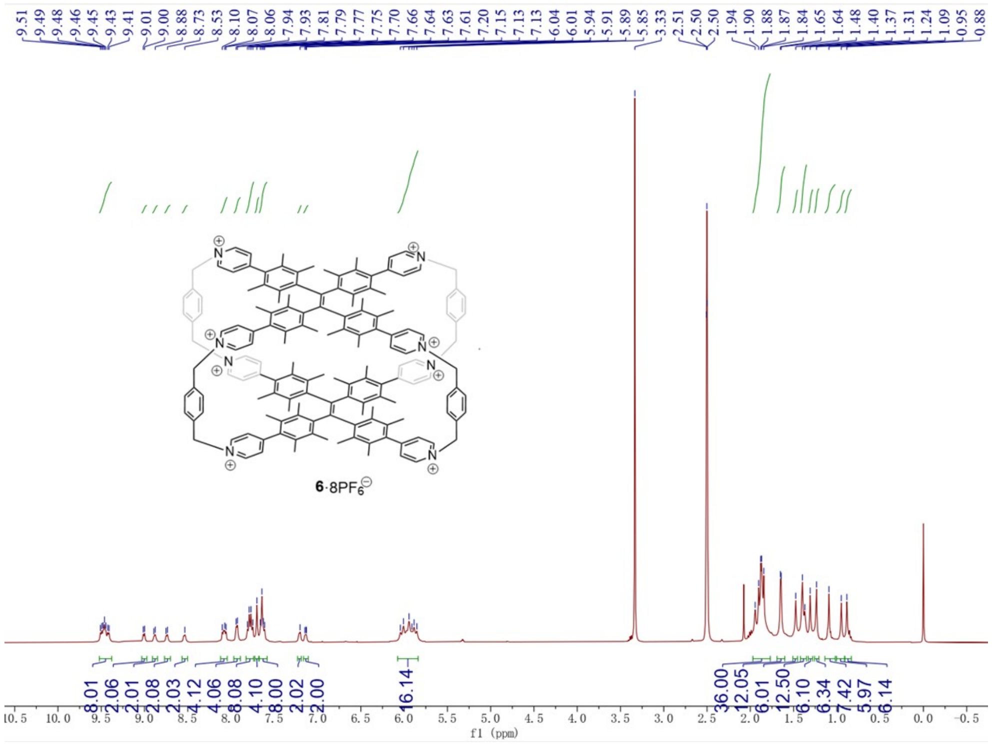

Figure S29. ^{1}H NMR spectrum of compound **6**·$8PF_6^-$ (400 MHz, DMSO).

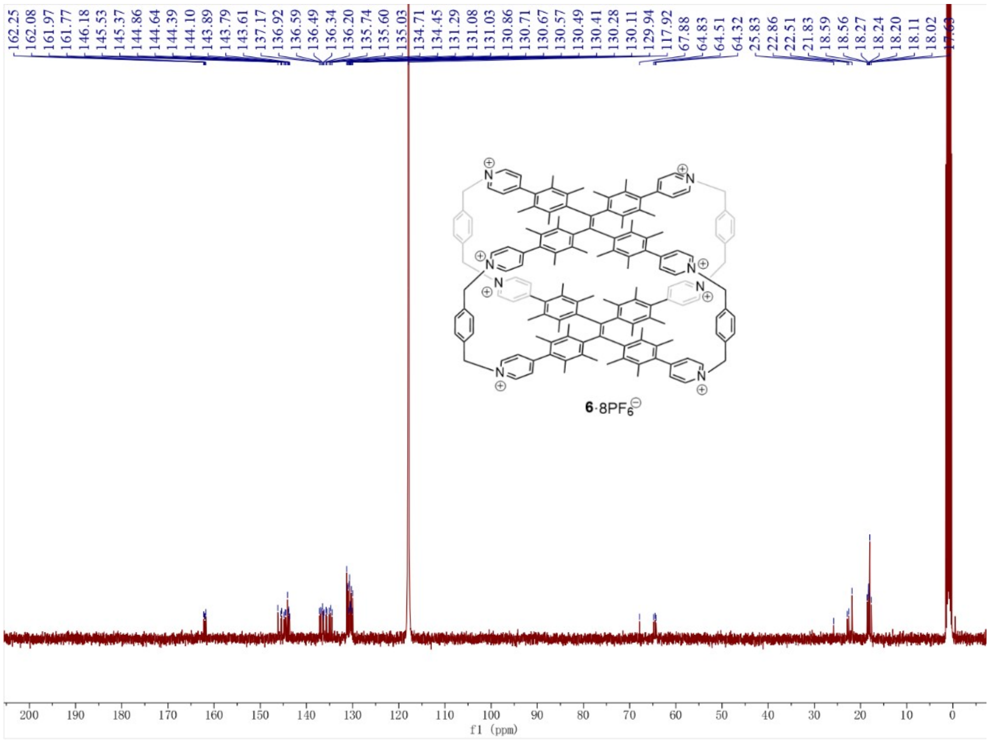

Figure S30. ^{13}C NMR spectrum of compound **6**·8PF$_6^-$ (101 MHz, CD$_3$CN).

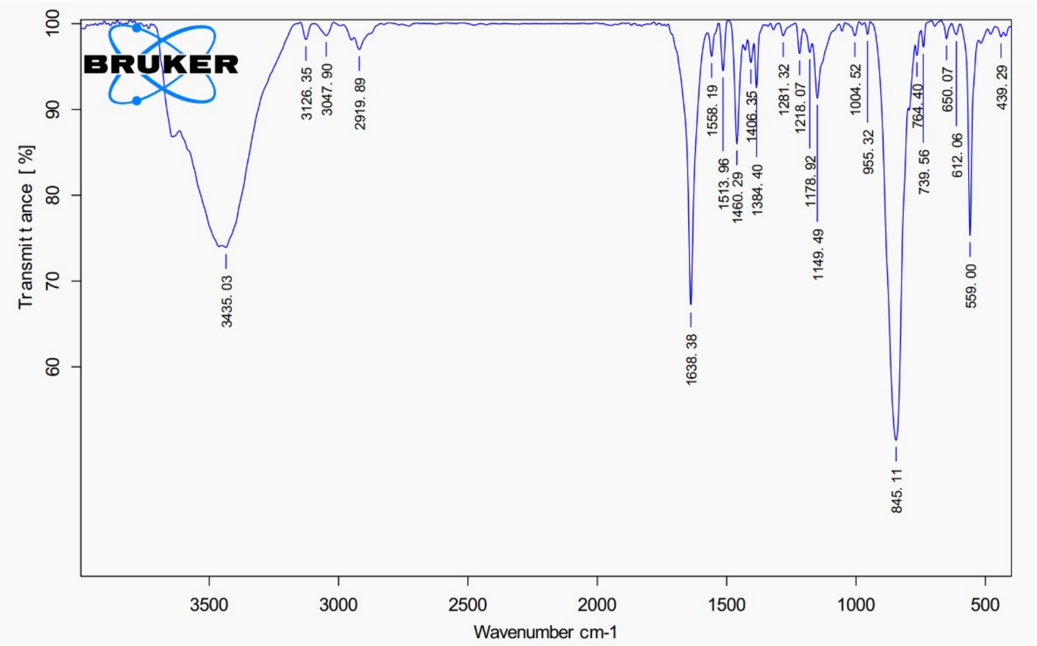

Figure S31. IR spectrum of compound **6**·8PF$_6^-$.

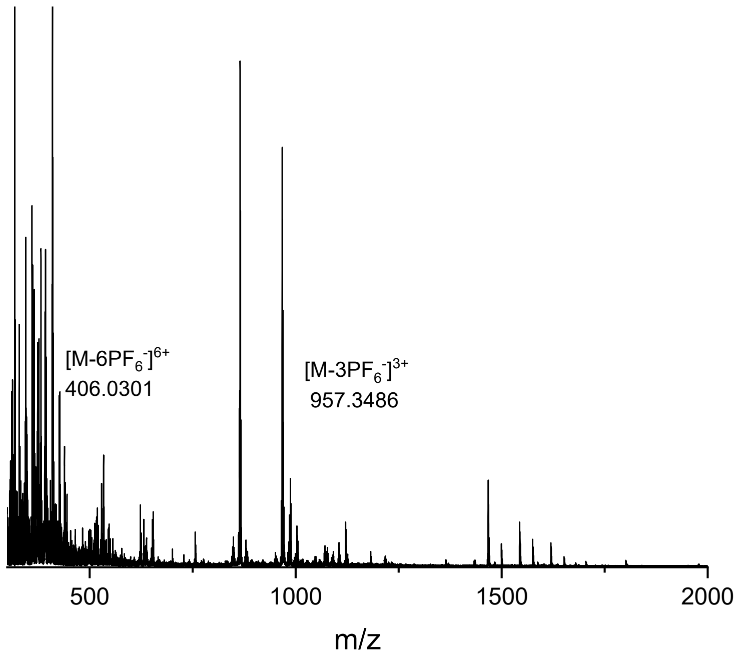

Figure S32. Experimental electrospray ionization mass spectra of **6**·8PF$_6^-$.

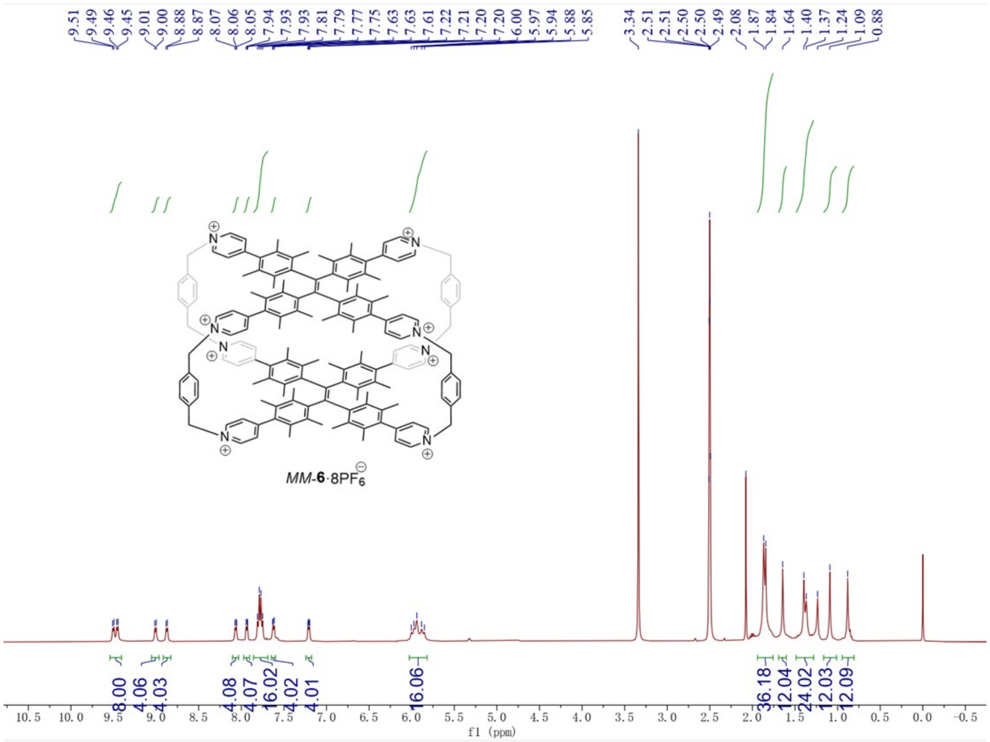

Figure S33. ^{1}H NMR spectrum of compound *MM*-**6**·8PF$_6^-$ (400 MHz, DMSO).

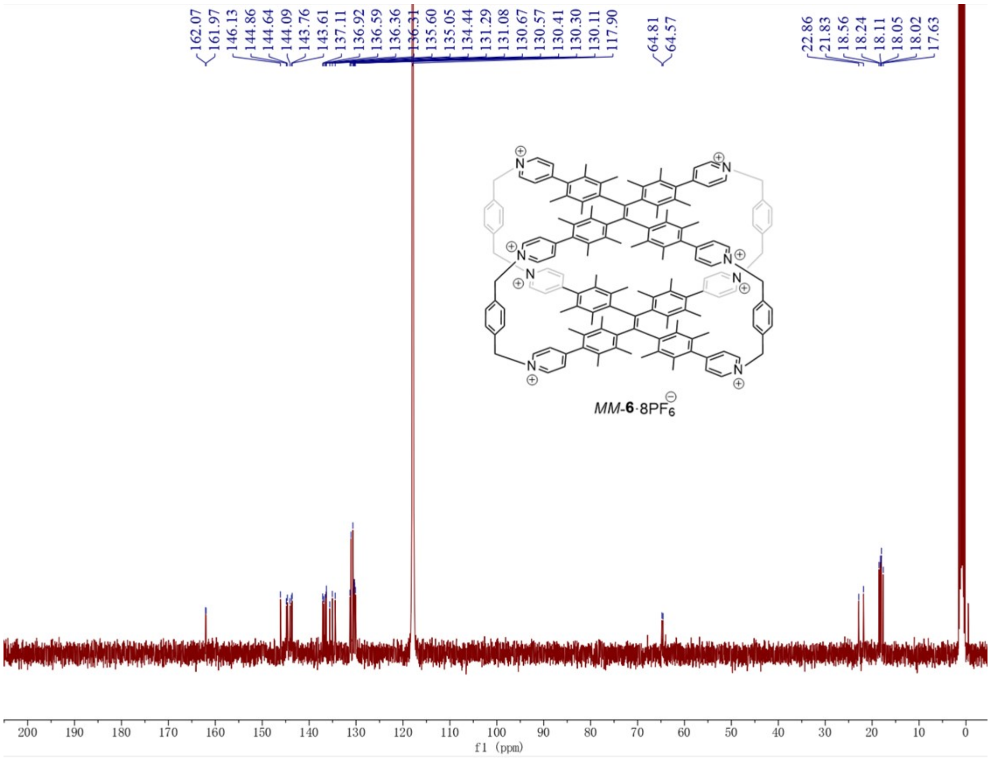

Figure S34. ^{13}C NMR spectrum of compound *MM*-**6**·$8PF_6^-$ (101 MHz, CD_3CN).

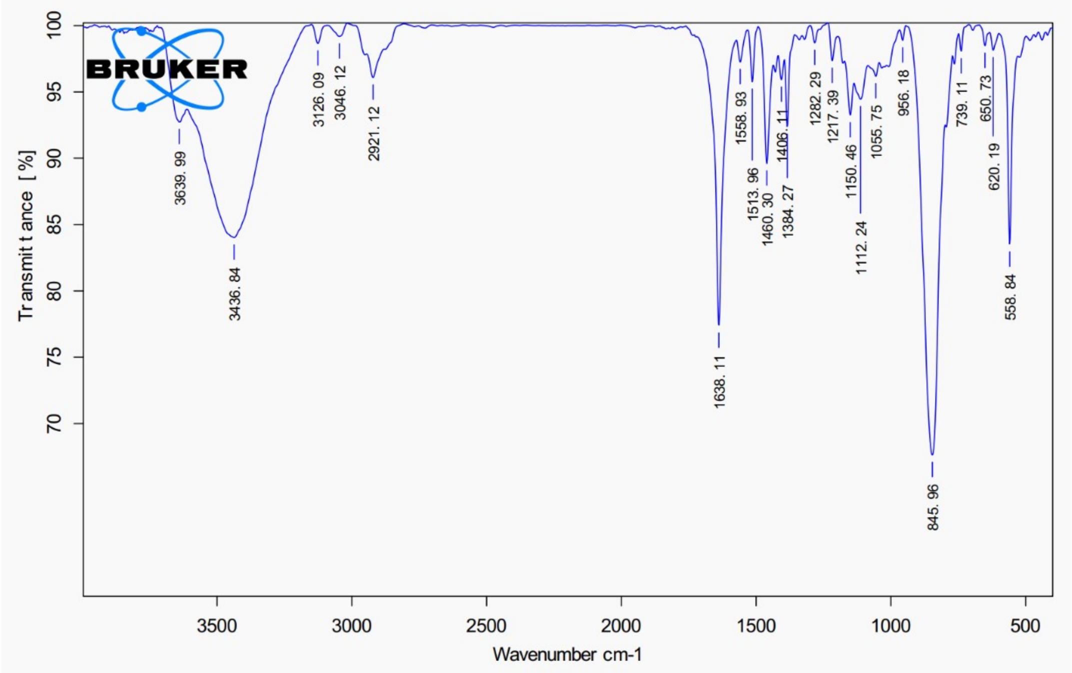

Figure S35. IR spectrum of compound *MM*-**6**·$8PF_6^-$.

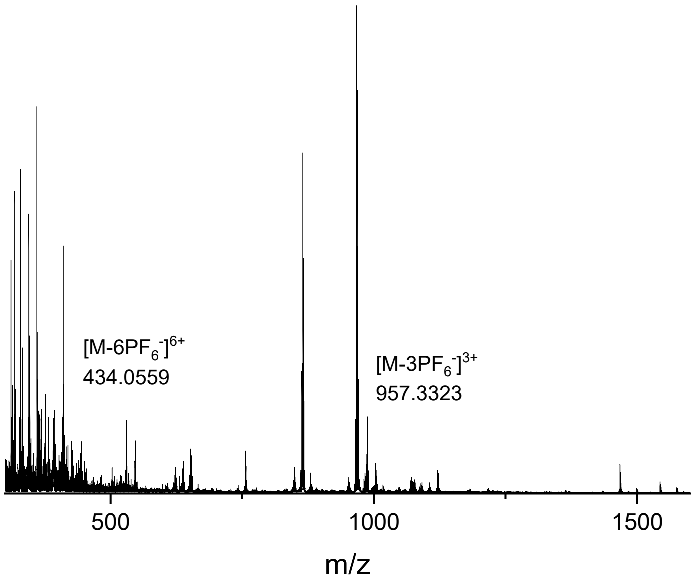

Figure S36. Experimental electrospray ionization mass spectra of *MM*-**6**·8PF$_6^-$.

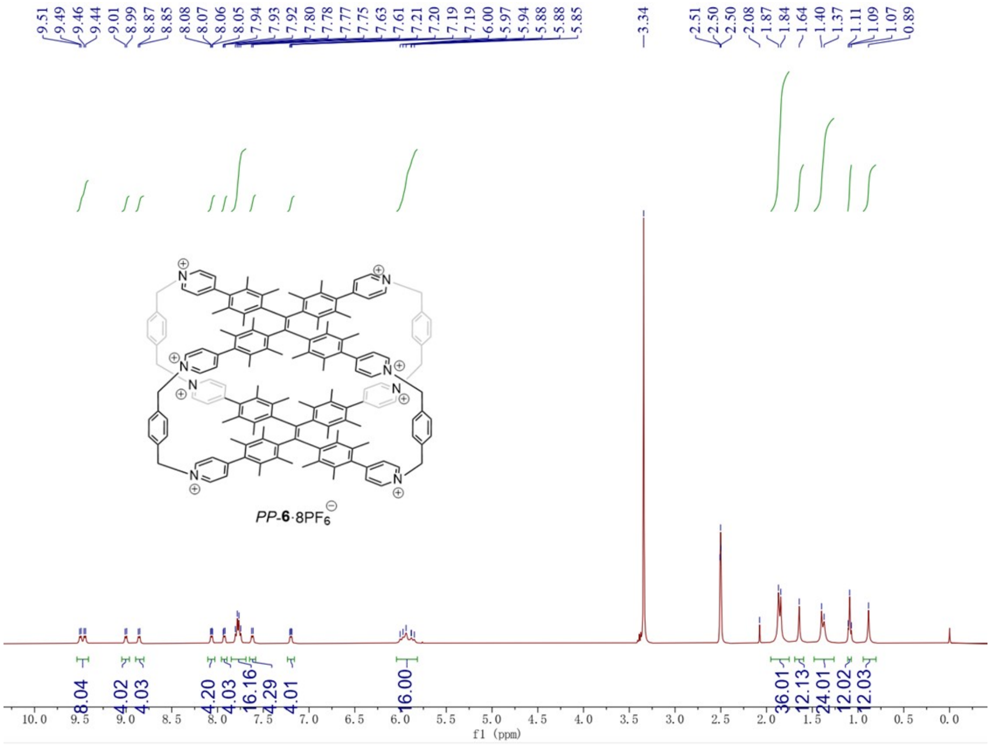

Figure S37. ^{1}H NMR spectrum of compound *PP*-**6**·8PF$_6^-$ (400 MHz, DMSO).

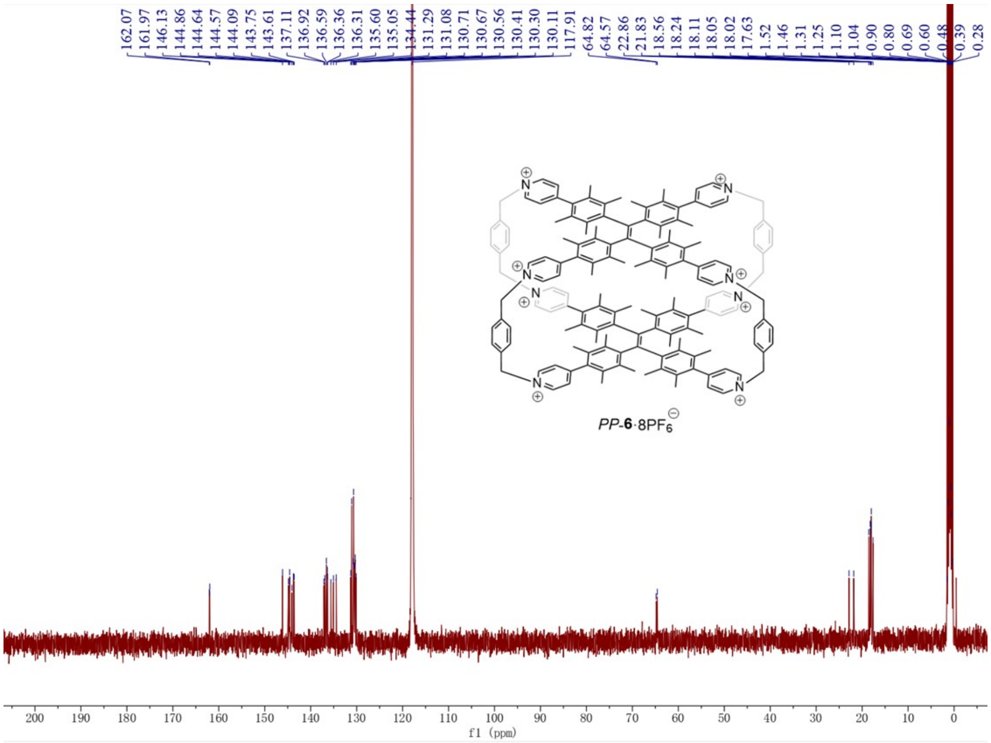

Figure S38. ^{13}C NMR spectrum of compound *PP*-**6**·8PF$_6^-$ (101 MHz, CD_3CN).

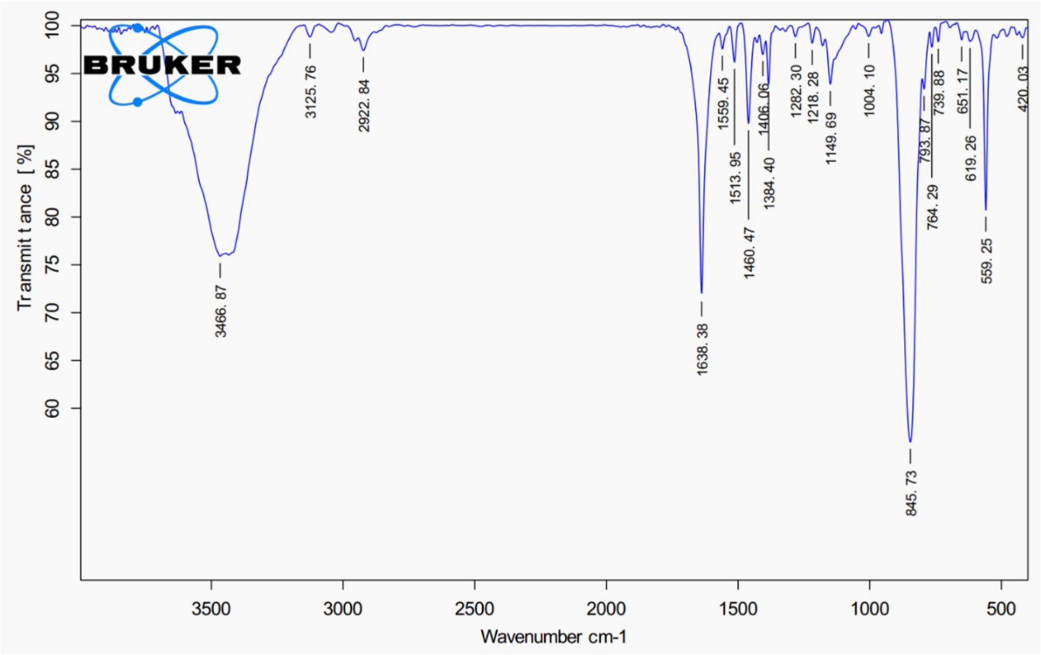

Figure S39. IR spectrum of compound *PP*-**6**·8PF$_6^-$.

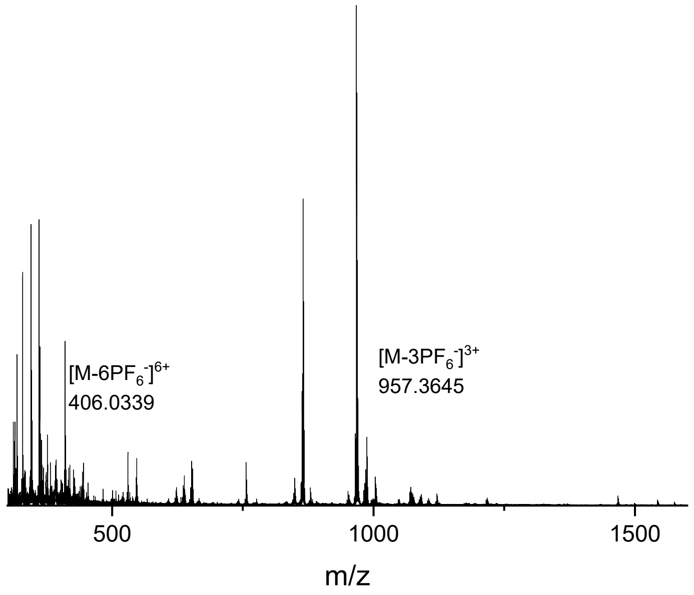

Figure S40. Experimental electrospray ionization mass spectra of *PP*-**6**·$8PF_6^-$.

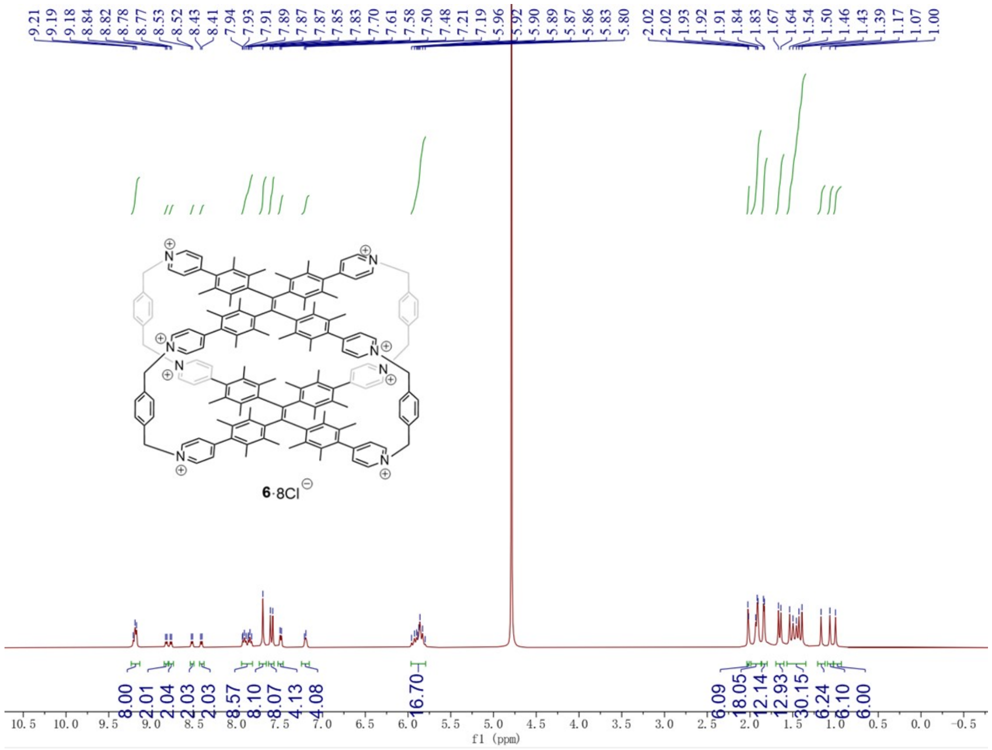

Figure S41. ^{1}H NMR spectrum of compound **6**·$8Cl^-$ (400 MHz, D_2O).

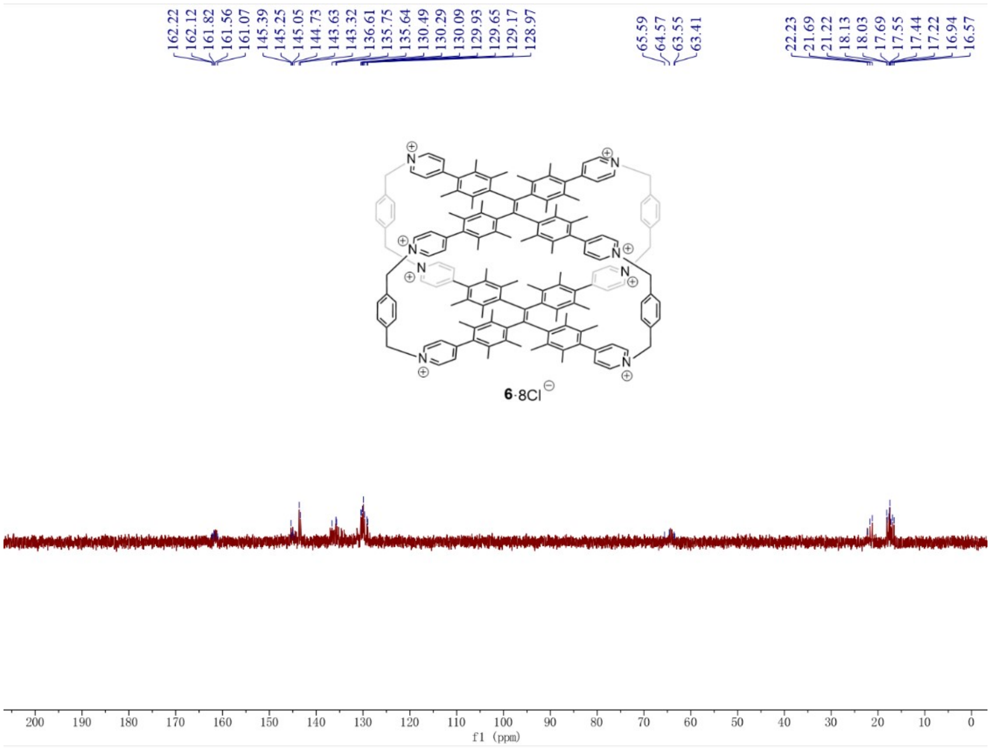

Figure S42. ^{13}C NMR spectrum of compound **6**·8Cl⁻ (101 MHz, D_2O).

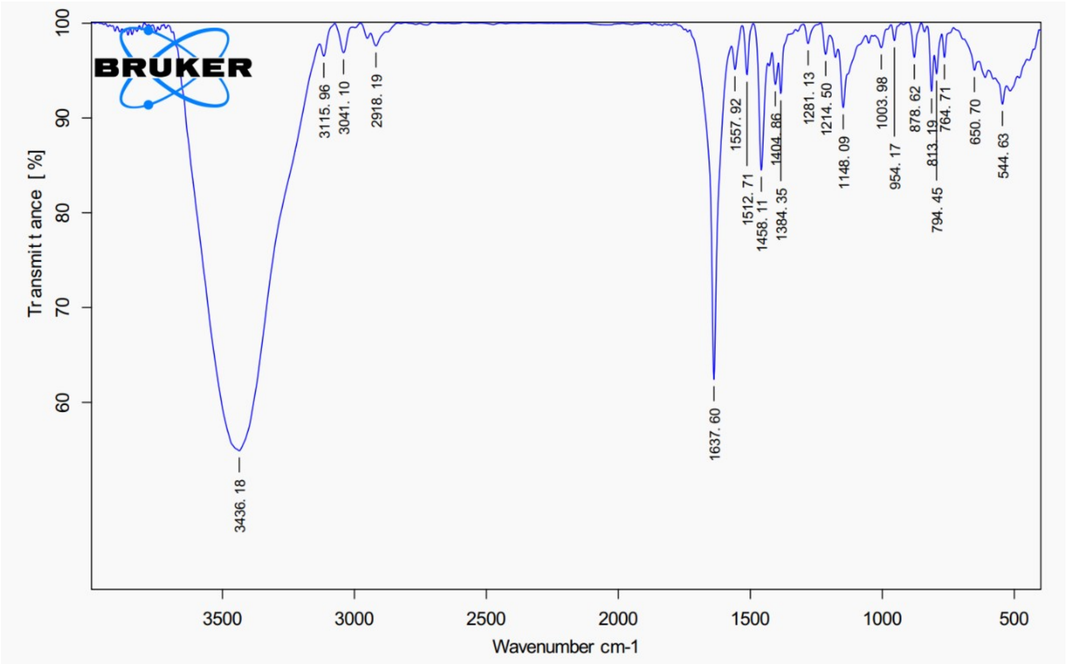

Figure S43. IR spectrum of compound **6**·8Cl⁻.

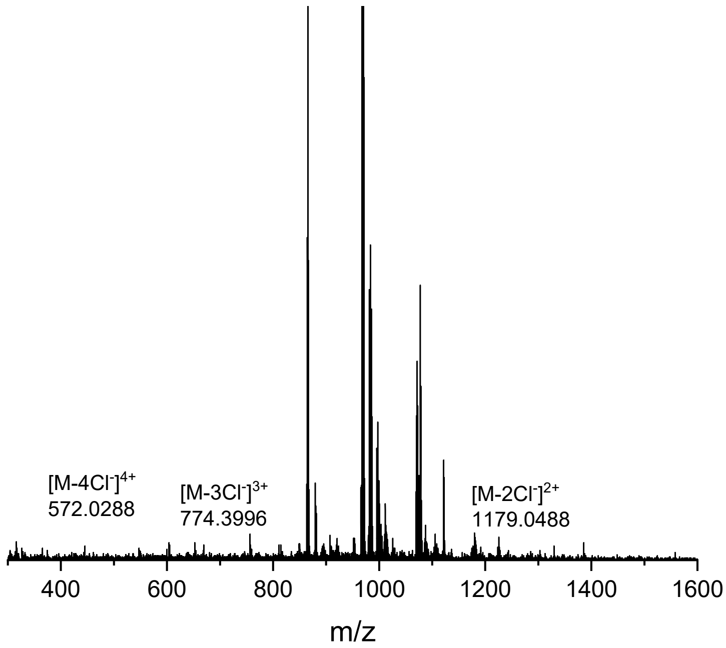

Figure S44. Experimental electrospray ionization mass spectra of **6**·8Cl⁻.

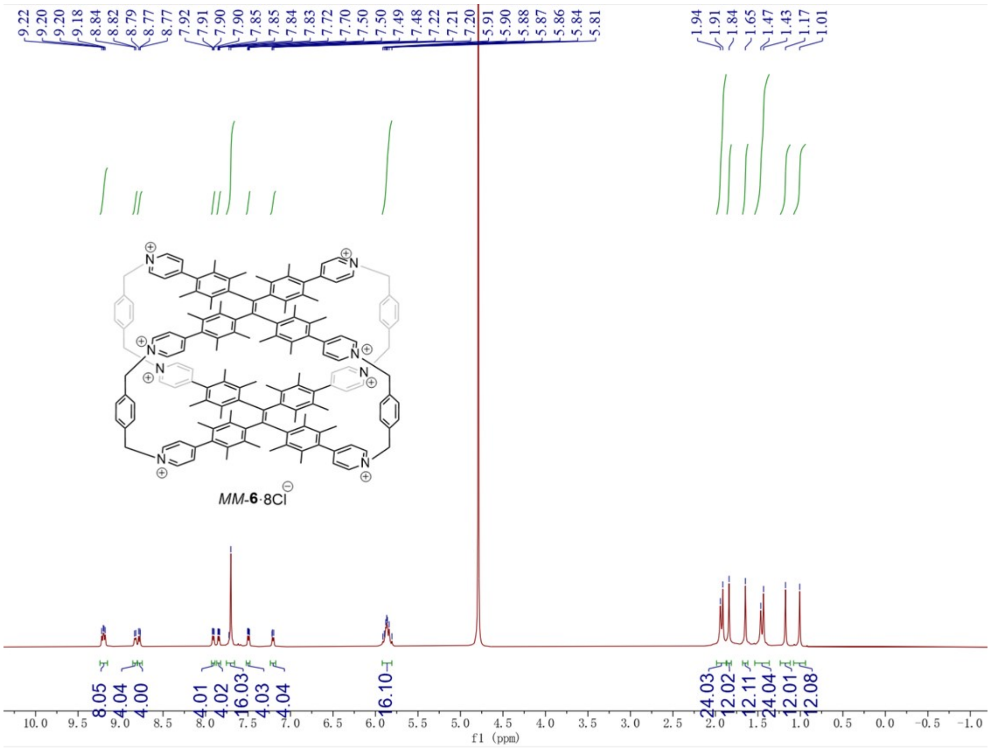

Figure S45. ^{1}H NMR spectrum of compound *MM*-**6**·8Cl⁻ (400 MHz, D_2O).

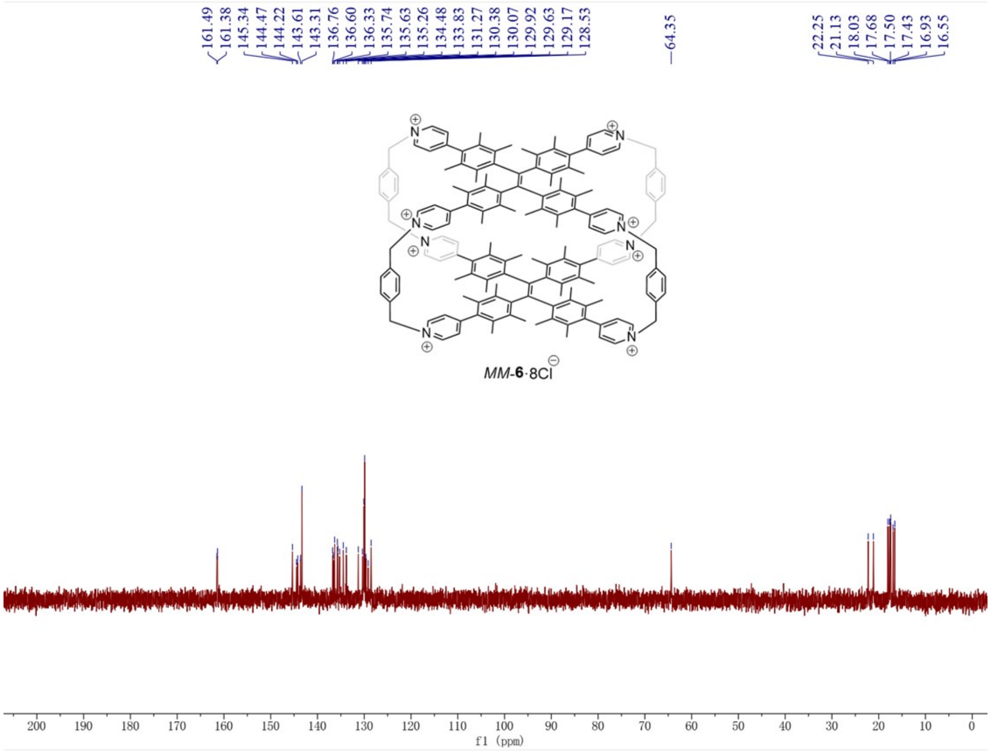

Figure S46. ^{13}C NMR spectrum of compound *MM*-**6**·8Cl^{-} (101 MHz, D_2O).

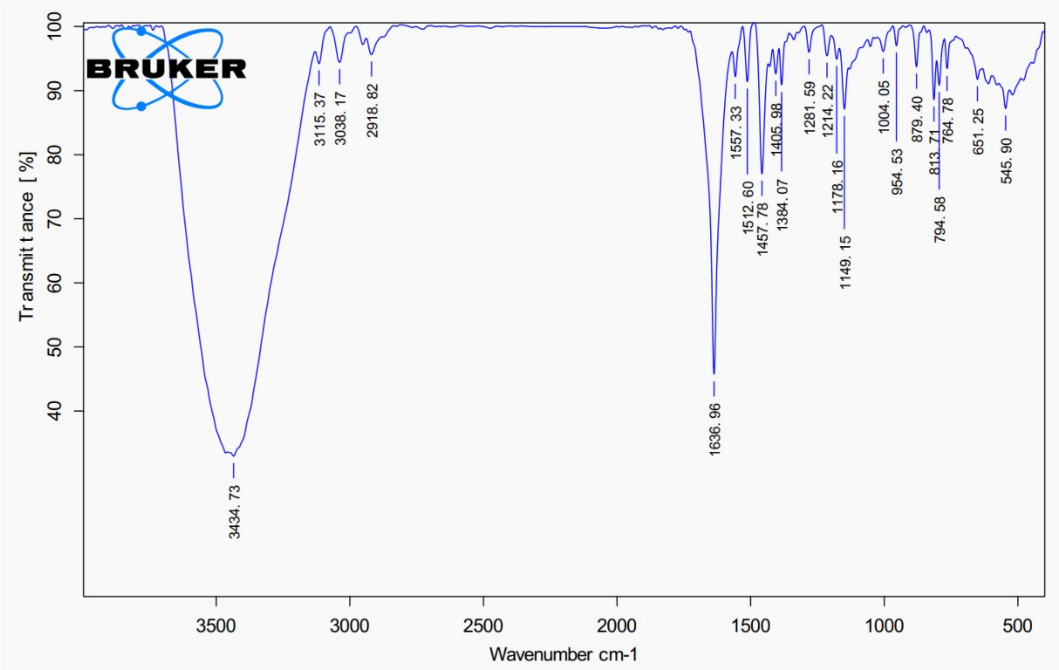

Figure S47. ^{1}IR spectrum of compound *MM*-**6**·8Cl^{-}.

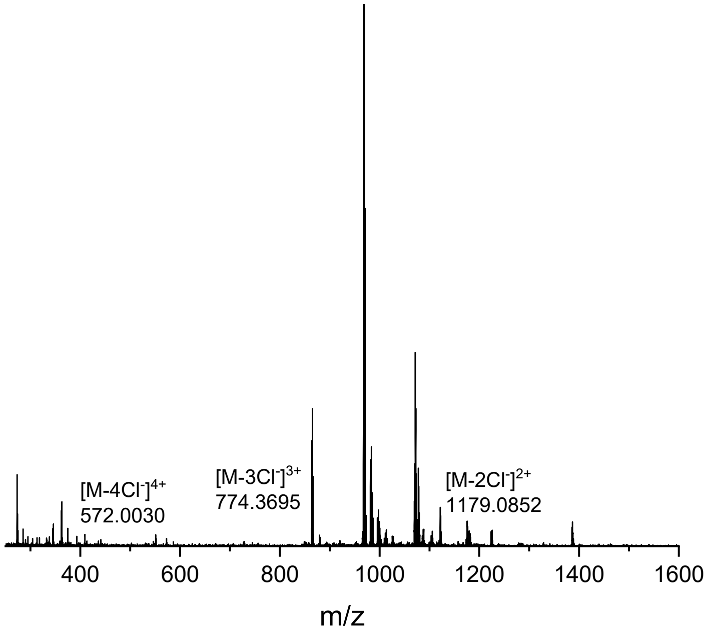

Figure S48. Experimental electrospray ionization mass spectra of *MM*-**6**·8Cl⁻.

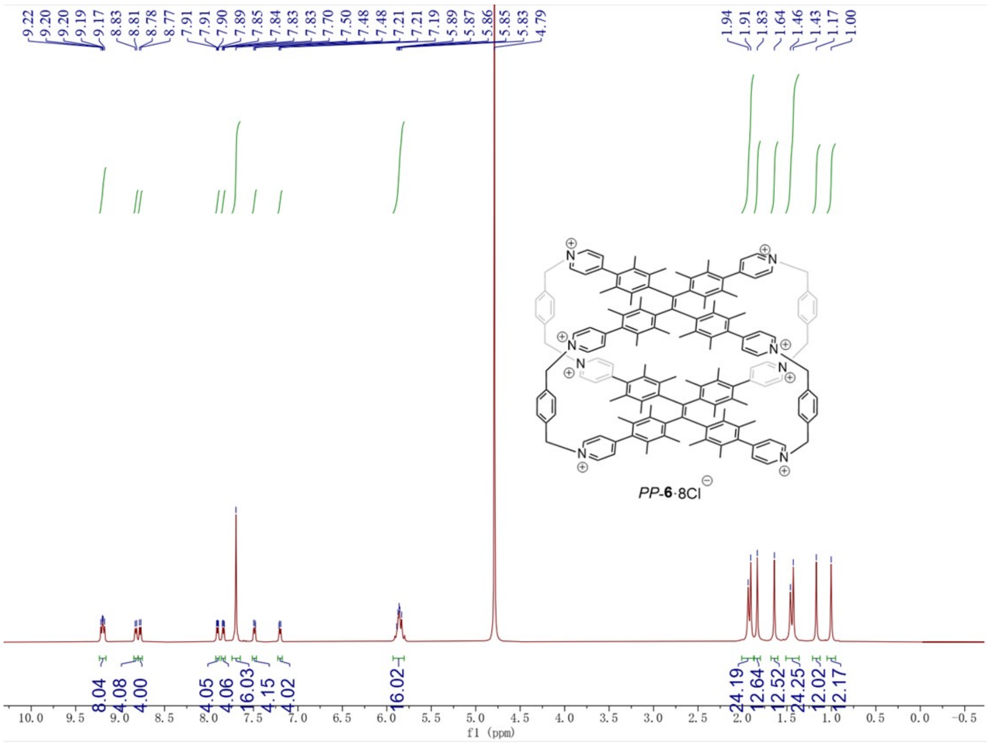

Figure S49. ^{1}H NMR spectrum of compound *PP*-**6**·8Cl⁻ (400 MHz, D_2O).

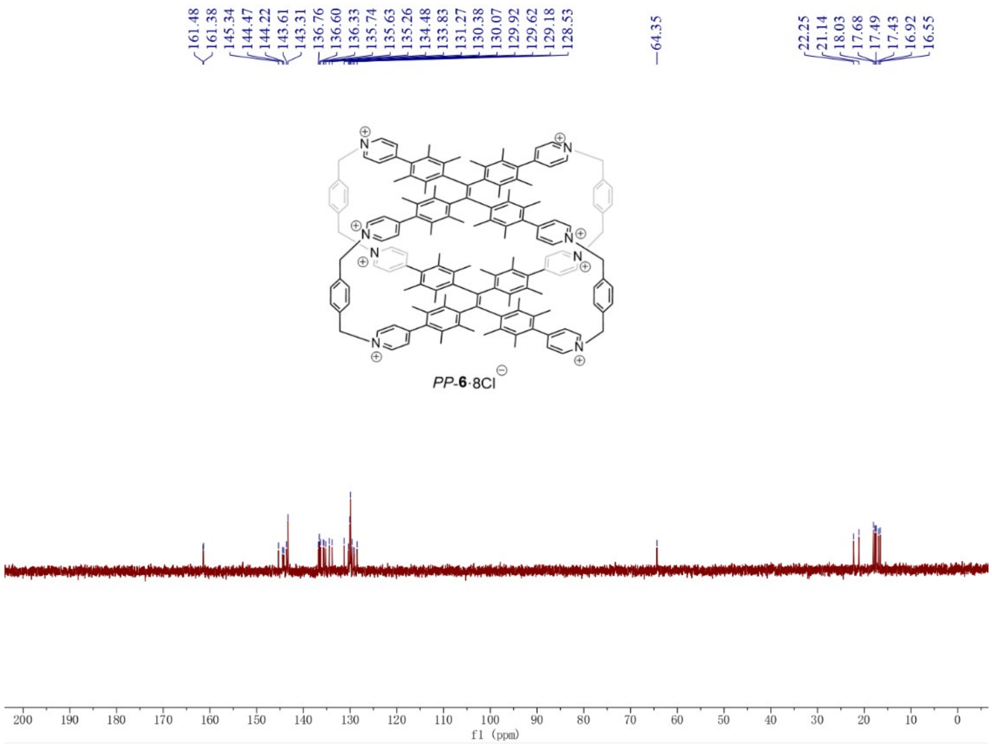

Figure S50. ^{13}C NMR spectrum of compound *PP*-**6**·8Cl⁻ (101 MHz, D_2O).

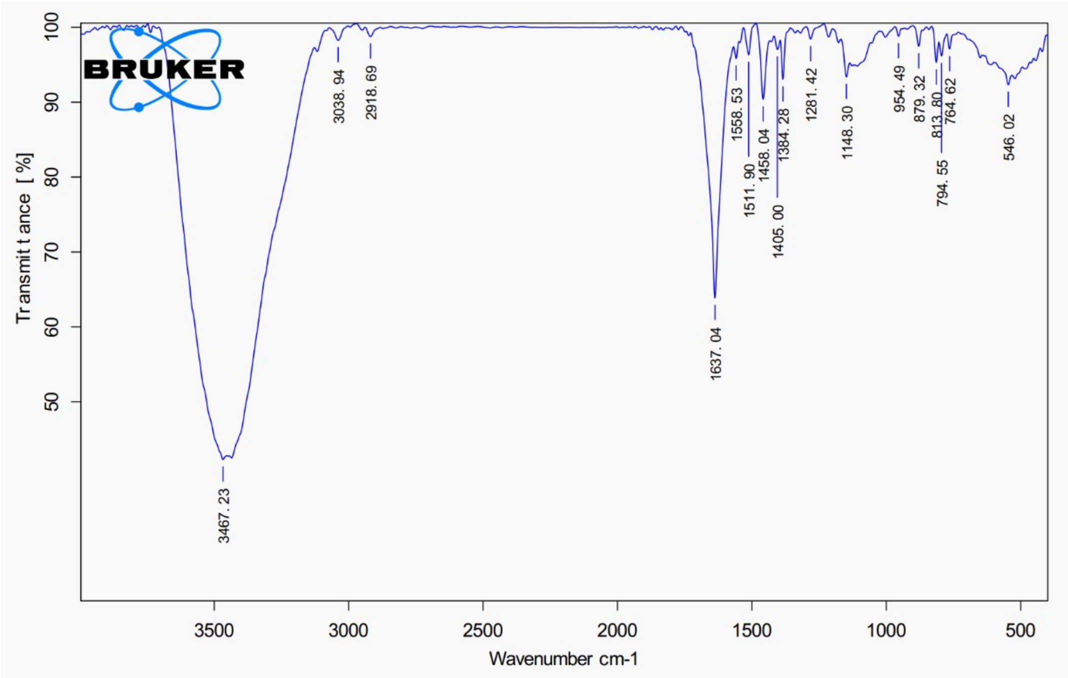

Figure S51. [1]IR spectrum of compound *PP*-**6**·8Cl⁻.

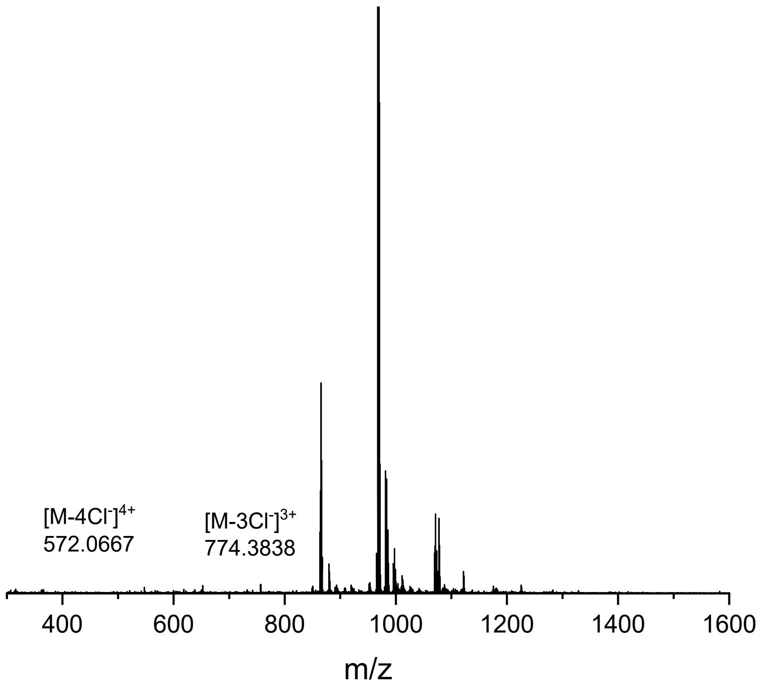

Figure S52. Experimental electrospray ionization mass spectra of *PP*-**6**·8Cl⁻.

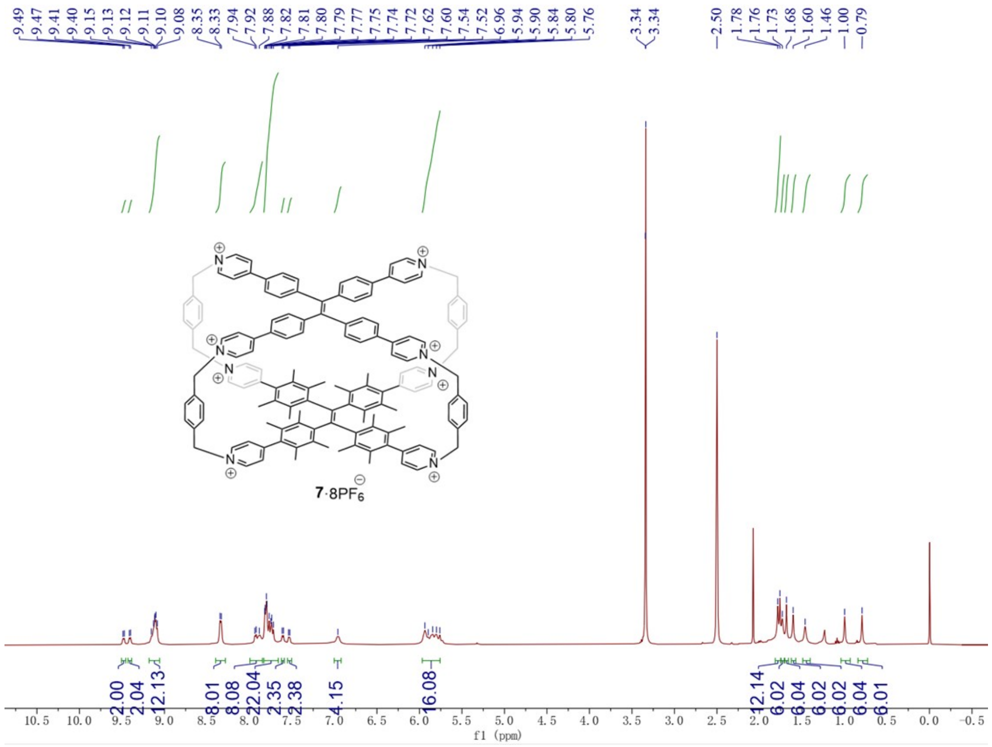

Figure S53. ^{1}H NMR spectrum of **7**·8PF_6^- (400 MHz, DMSO).

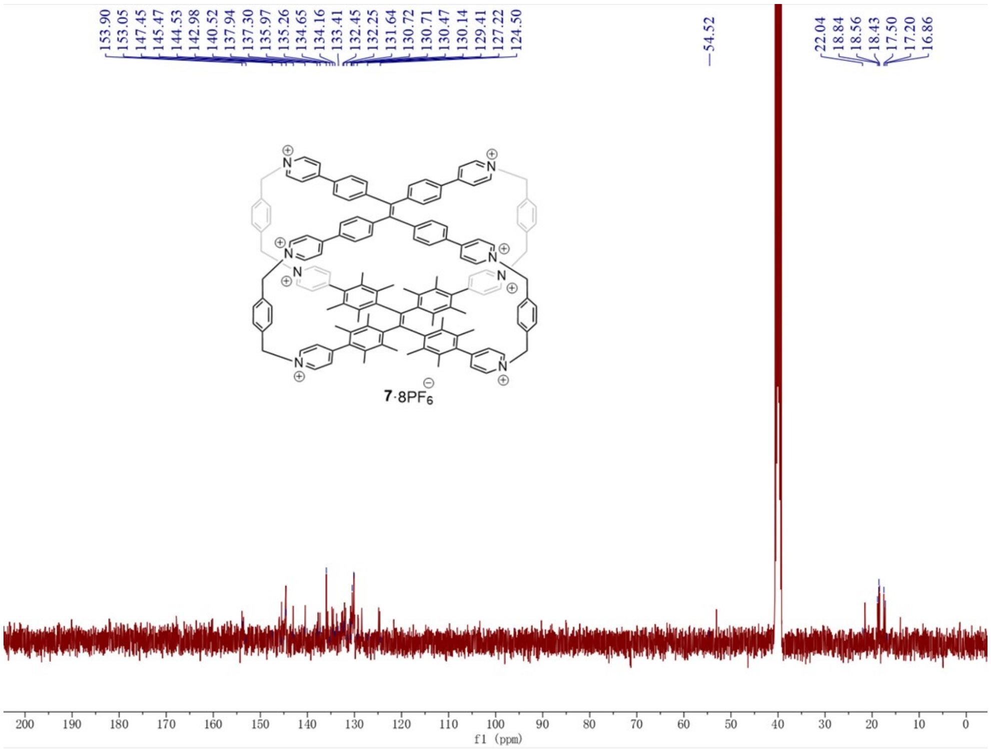

Figure S54. ^{13}C NMR spectrum of **7**·8PF_6^- (101 MHz, DMSO).

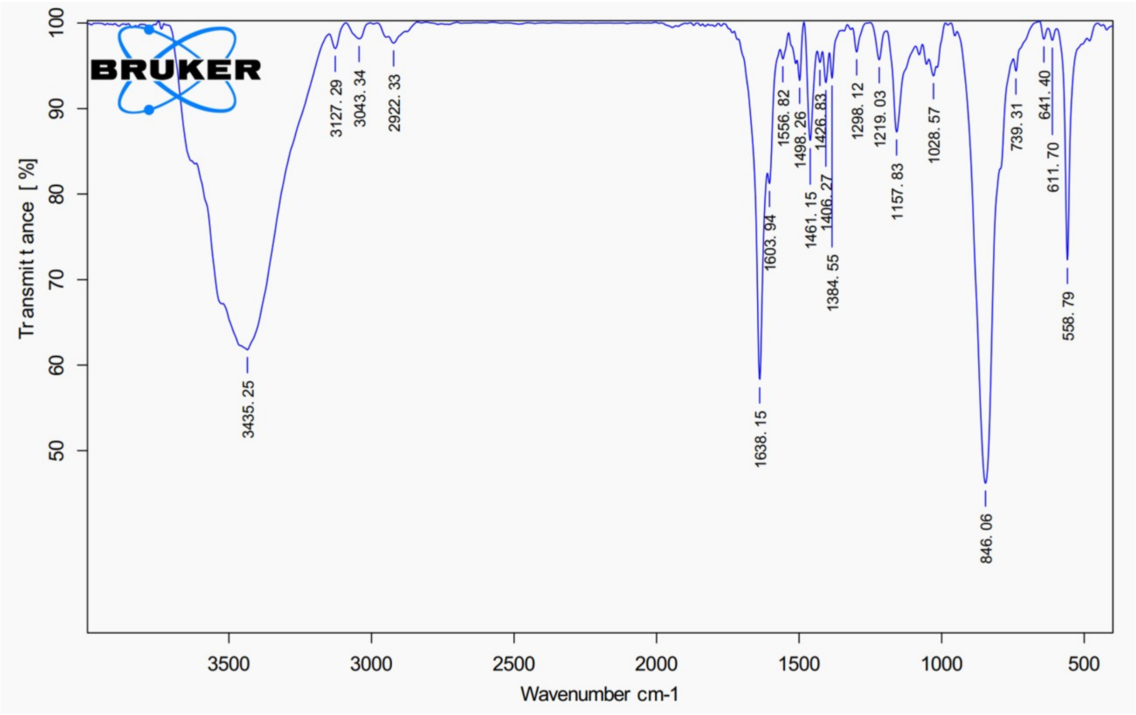

Figure S55. IR spectrum of **7**·8PF_6^-.

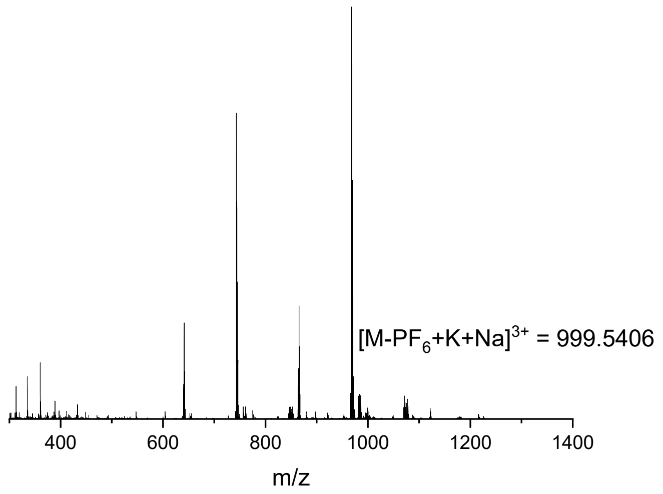

Figure S56. Experimental electrospray ionization mass spectra of **7**·8PF$_6^-$.

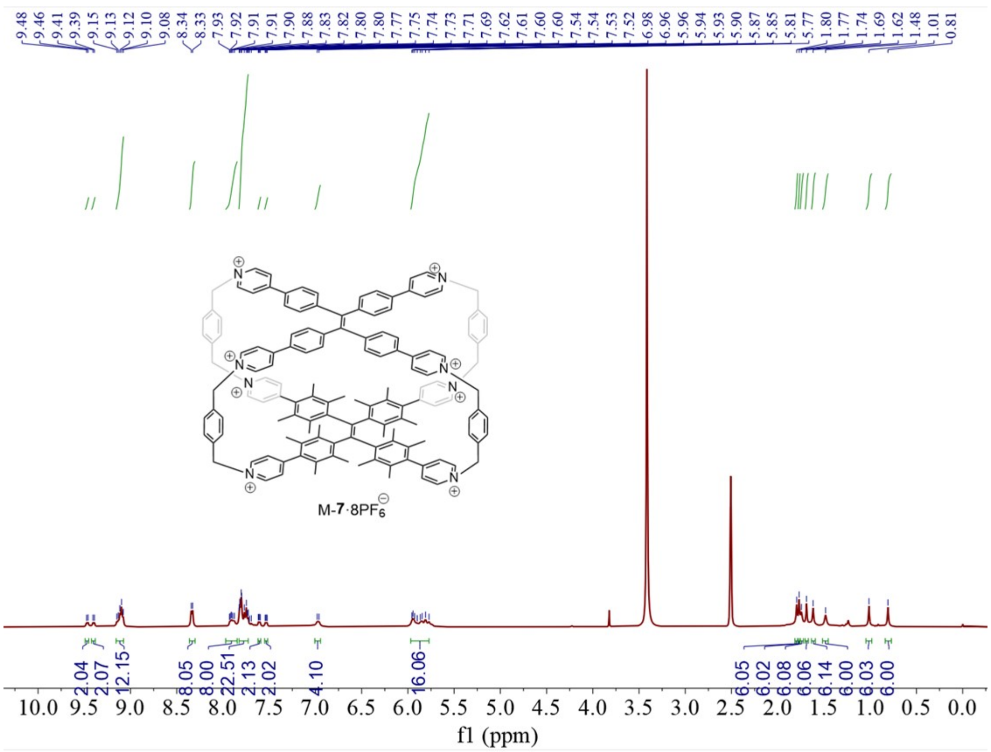

Figure S57. ^{1}H NMR spectrum of *M*-**7**·8PF$_6^-$ (400 MHz, DMSO).

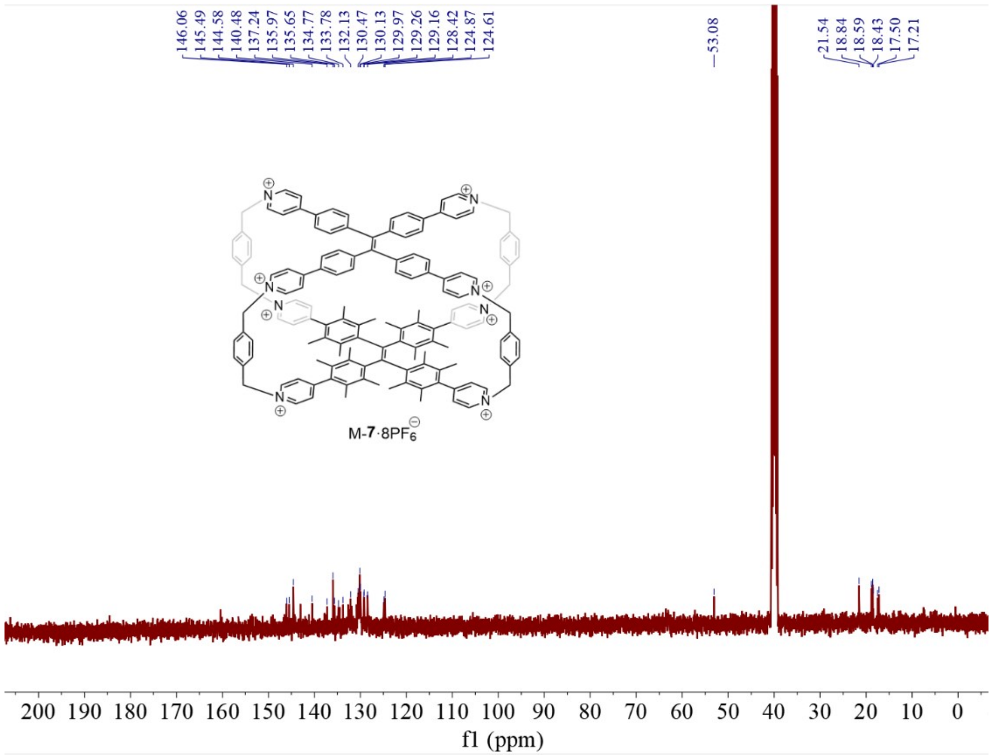

Figure S58. ^{13}C NMR spectrum of *M*-**7**·8PF$_6^-$ (101 MHz, DMSO).

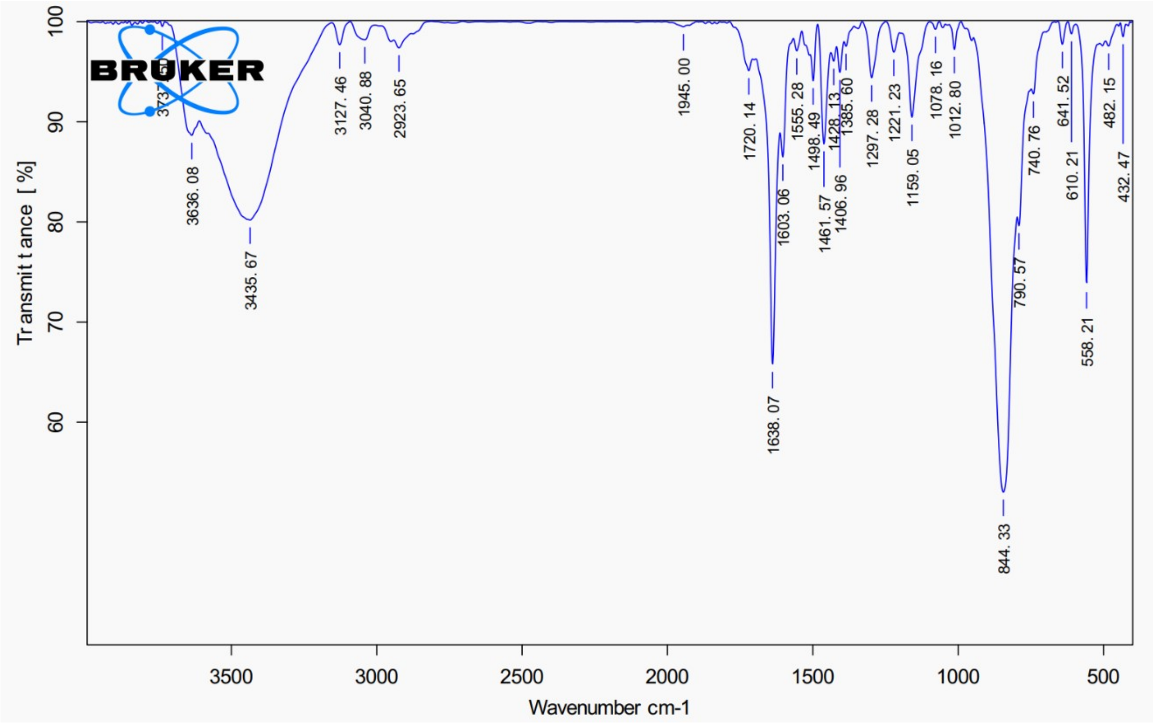

Figure S59. IR spectrum of *M*-**7**·8PF$_6^-$.

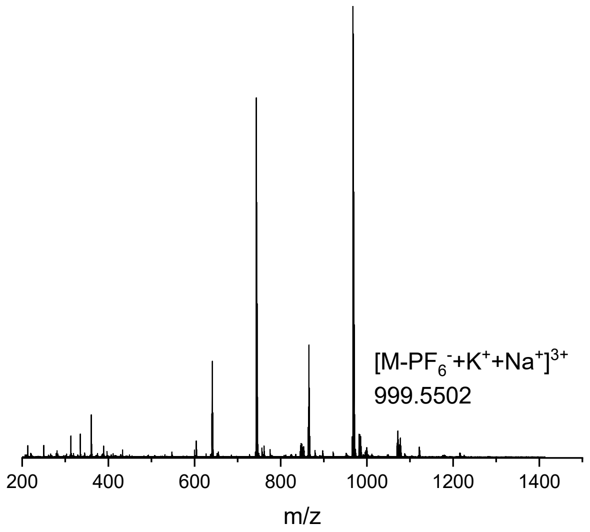

Figure S60. Experimental electrospray ionization mass spectra of *M*-**7**·8PF$_6^-$.

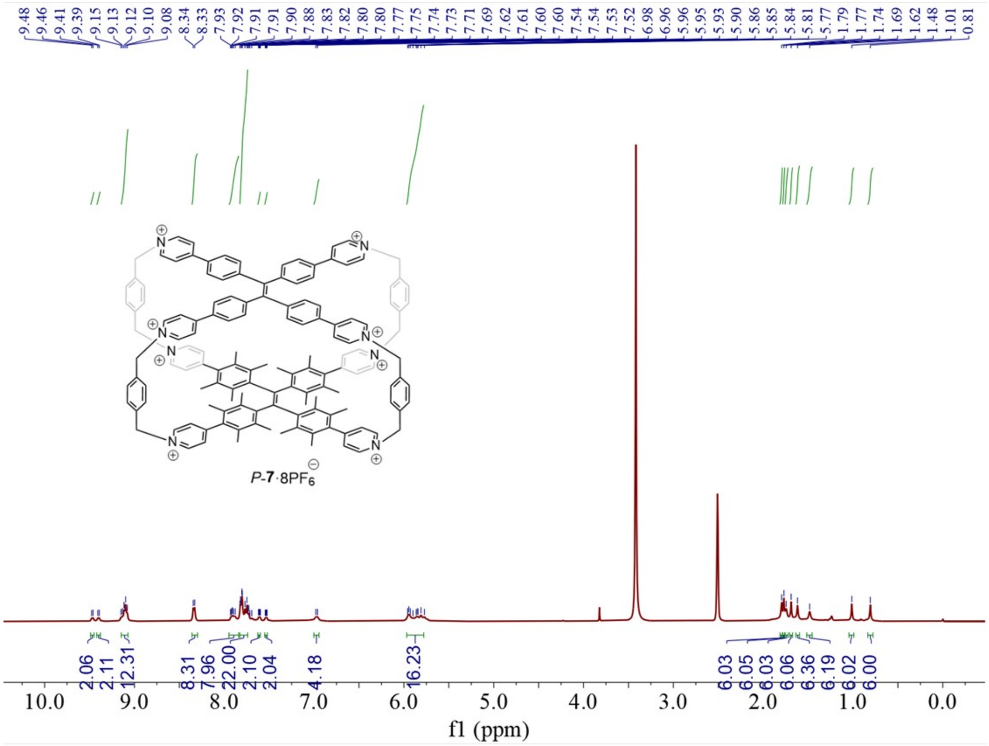

Figure S61. ^{1}H NMR spectrum of *P*-**7**·8PF$_6^-$ (400 MHz, DMSO).

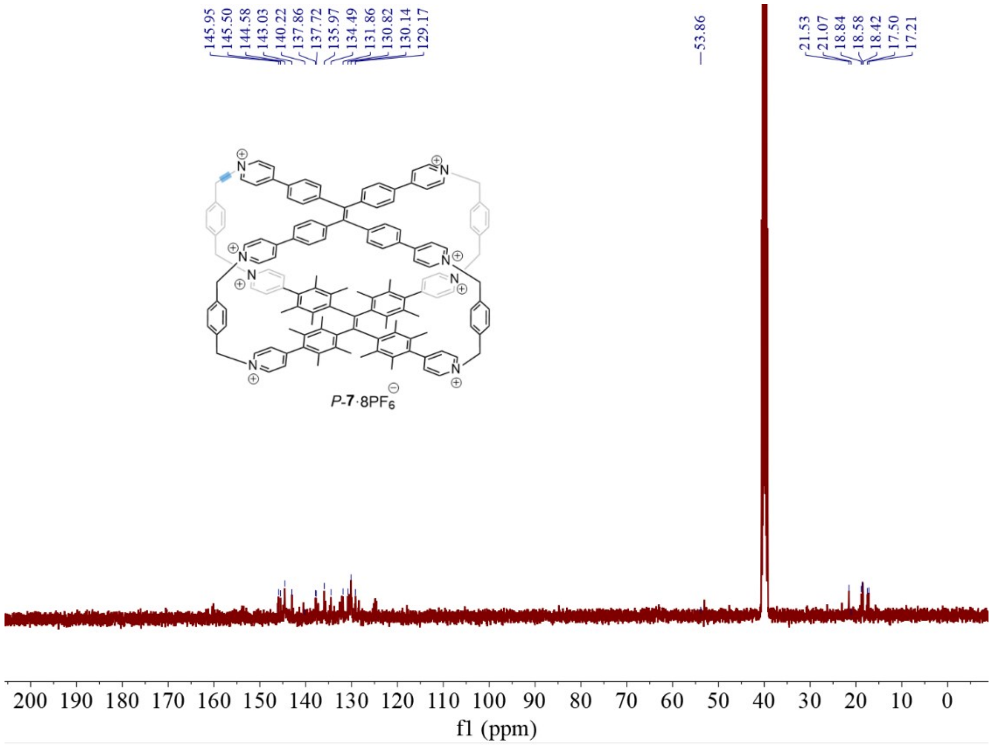

Figure S62. ^{13}C NMR spectrum of *P*-**7**·8PF_6^- (101 MHz, DMSO).

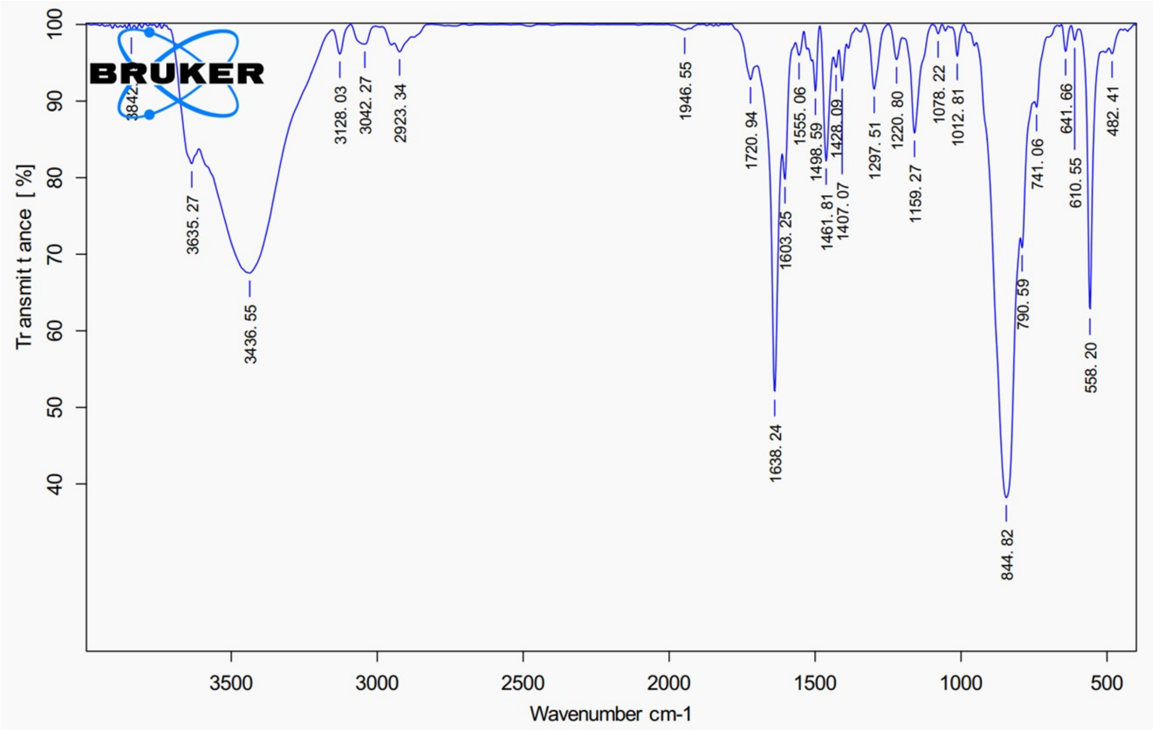

Figure S63. IR spectrum of *P*-**7**·8PF_6^-.

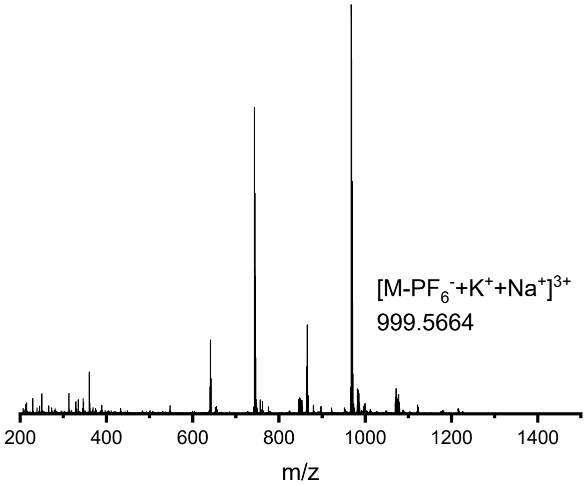

Figure S64. Experimental electrospray ionization mass spectra of *P*-**7**·$8PF_6^-$.

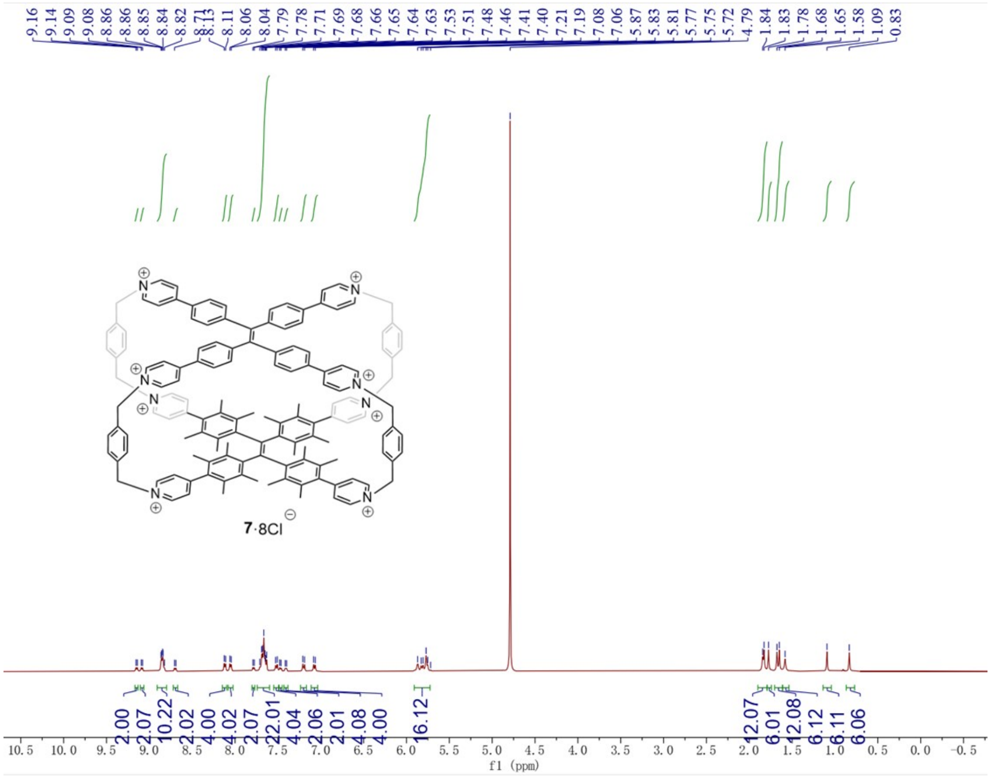

Figure S65. ^1H NMR spectrum of **7**·$8Cl^-$ (400 MHz, D_2O).

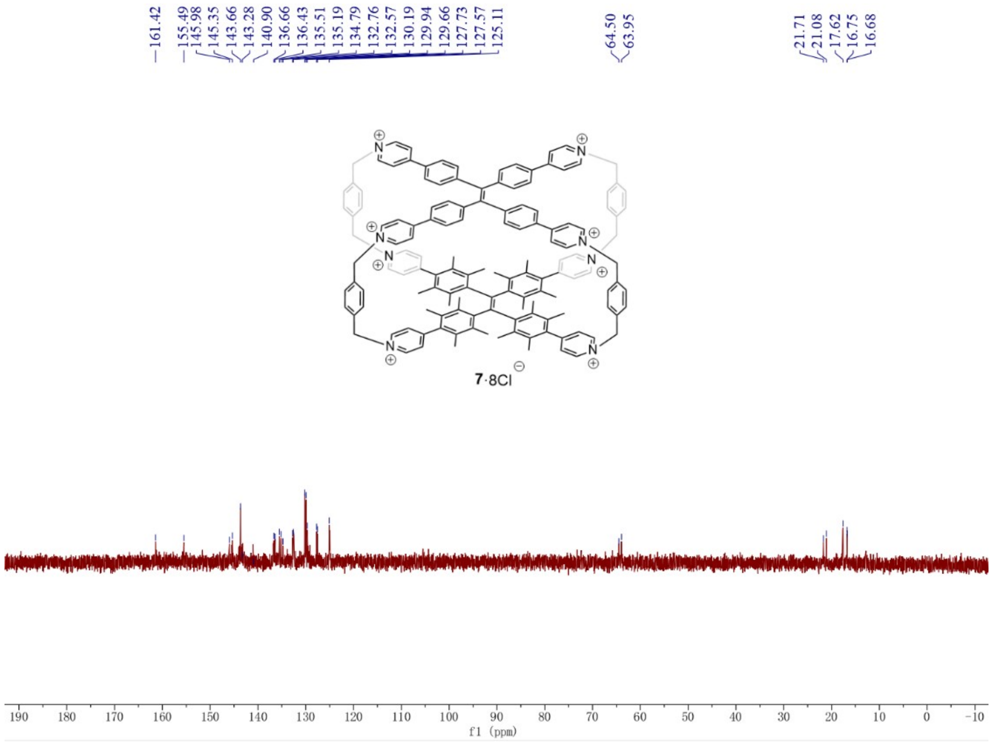

Figure S66. ^{13}C NMR spectrum of **7**·8Cl^{-} (101 MHz, D_2O).

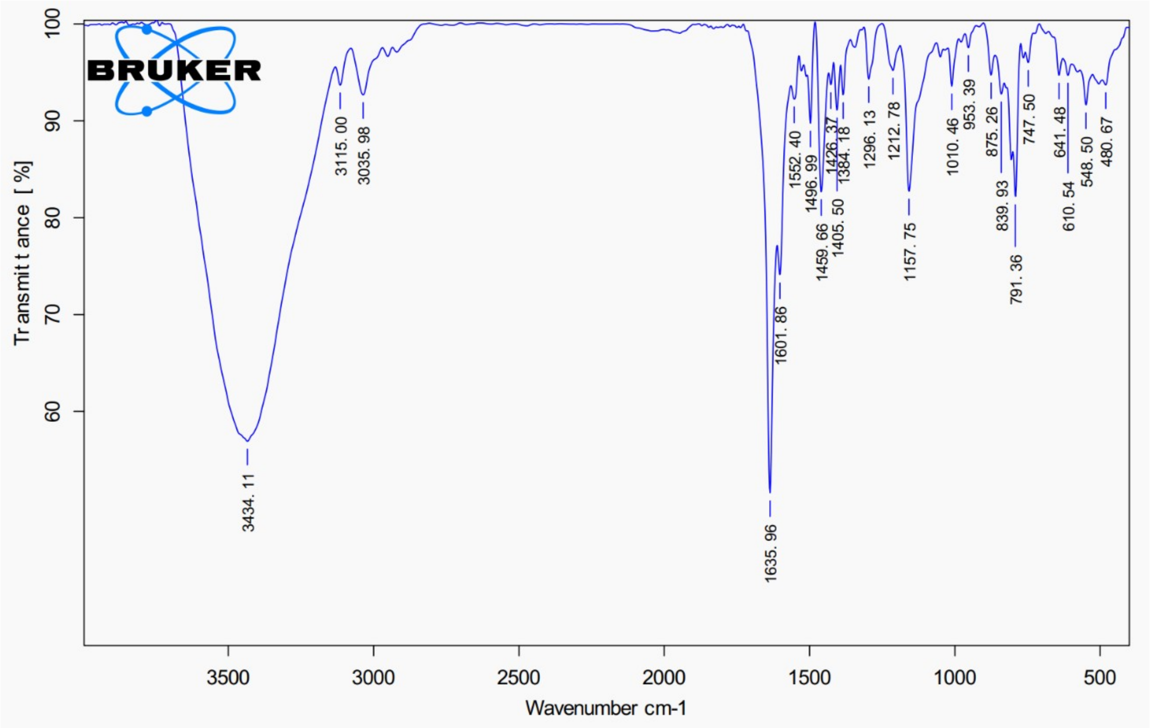

Figure S67. IR spectrum of **7**·8Cl^{-}.

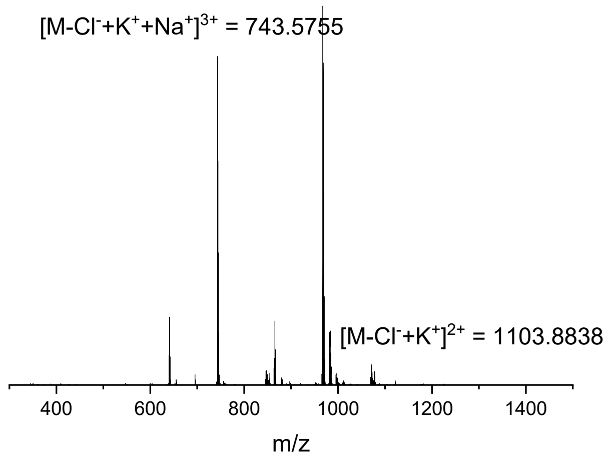

Figure S68. Experimental electrospray ionization mass spectra of **7**·8Cl⁻.

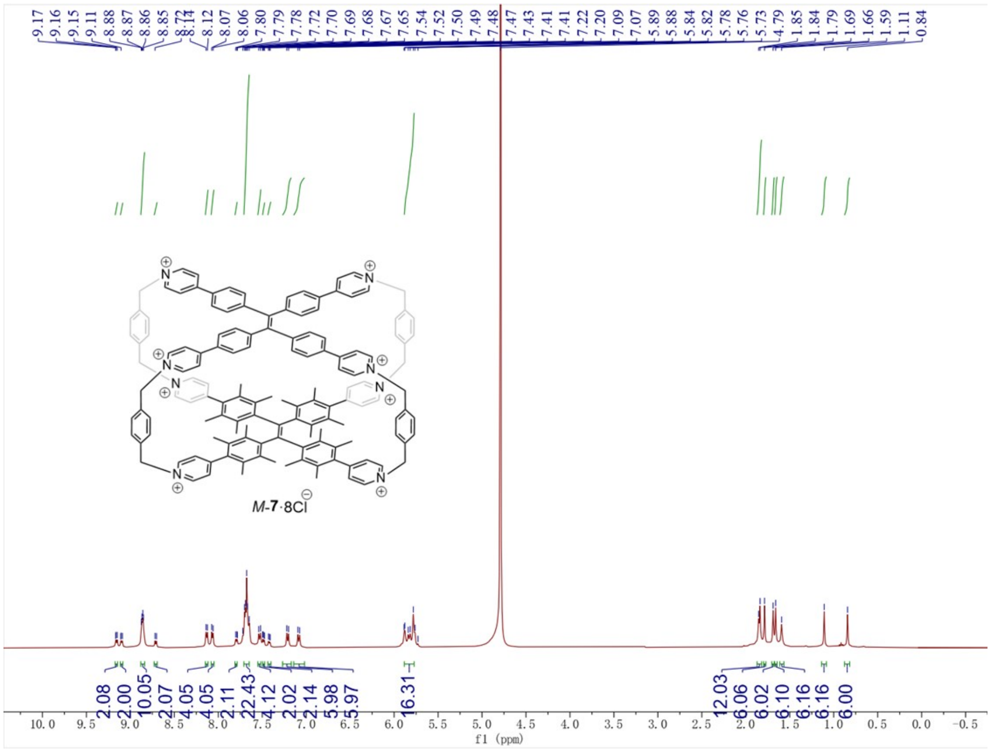

Figure S69. ¹H NMR spectrum of *M*-**7**·8Cl⁻ (400 MHz, D_2O).

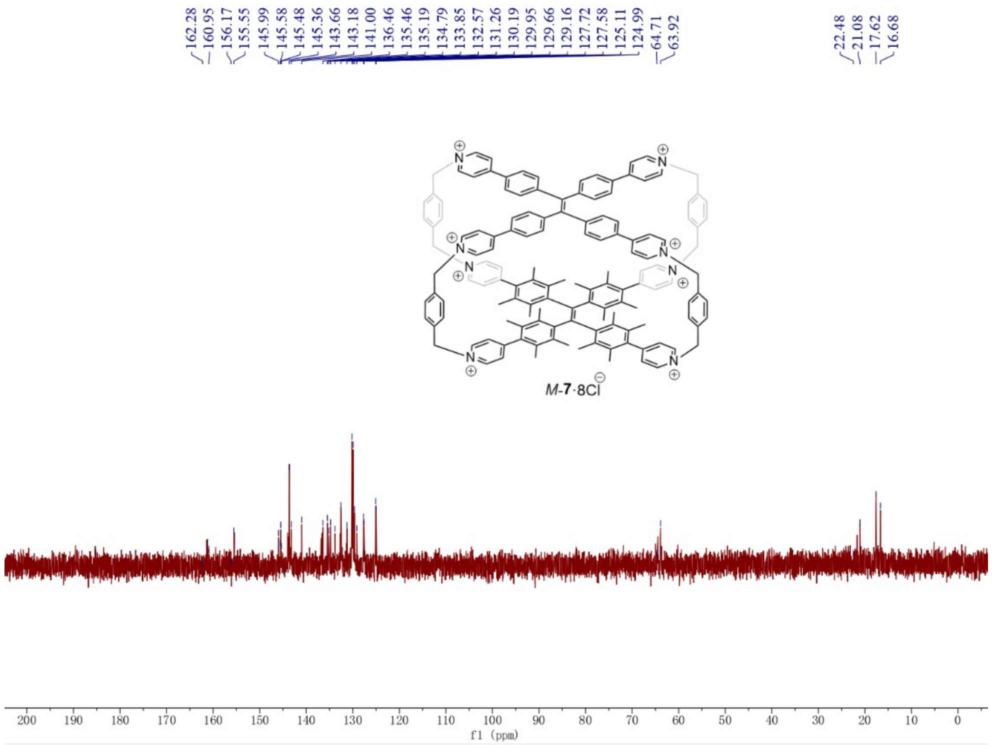

Figure S70. ^{13}C NMR spectrum of *M*-**7**·8Cl⁻ (101 MHz, D_2O).

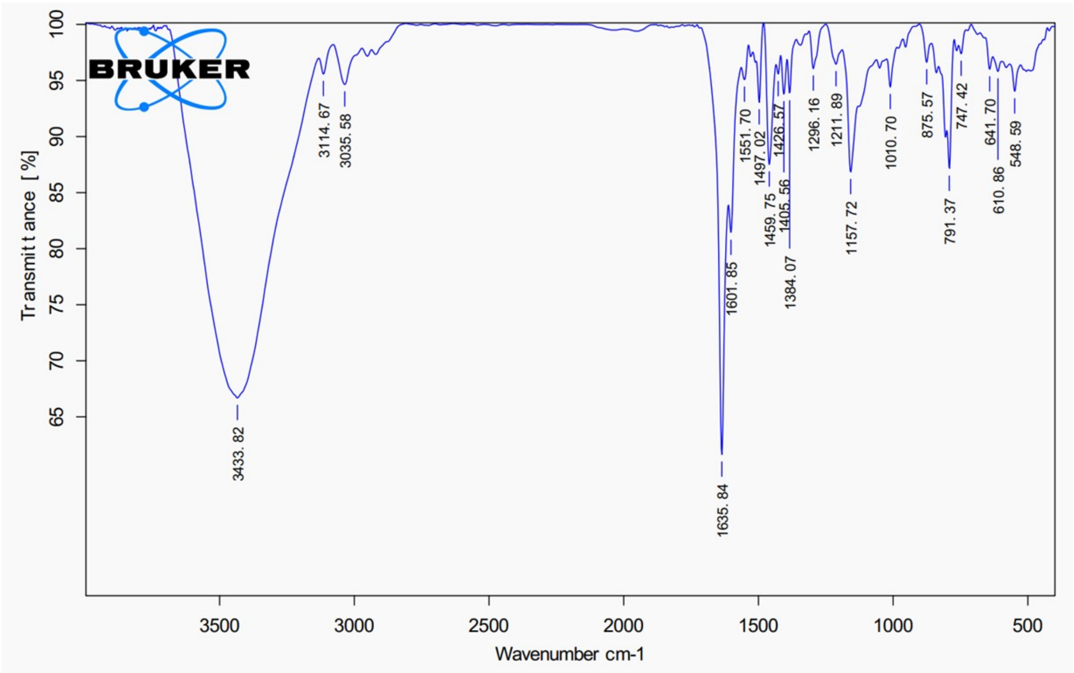

Figure S71. IR spectrum of *M*-**7**·8Cl⁻.

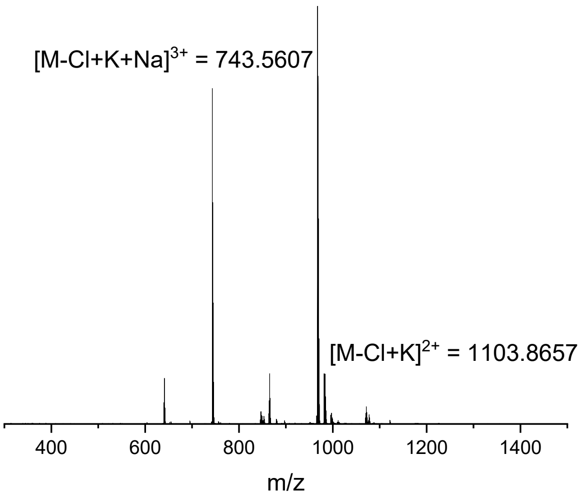

Figure S72. Experimental electrospray ionization mass spectra of *M*-**7**·8Cl⁻.

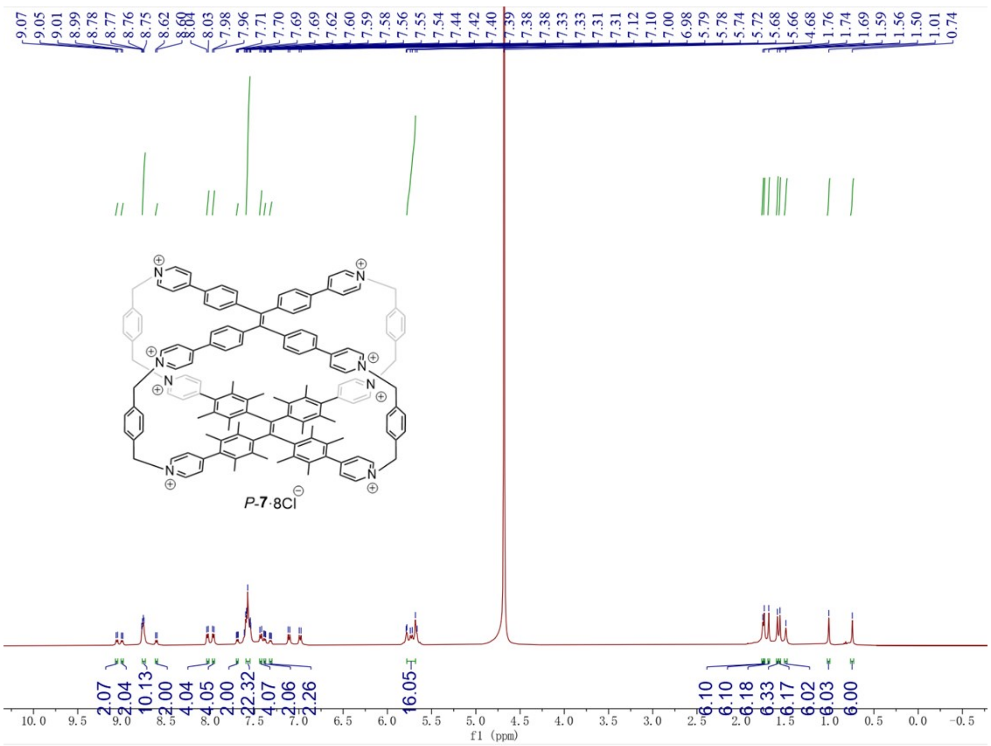

Figure S73. ^{1}H NMR spectrum of *P*-**7**·8Cl⁻ (400 MHz, D_2O).

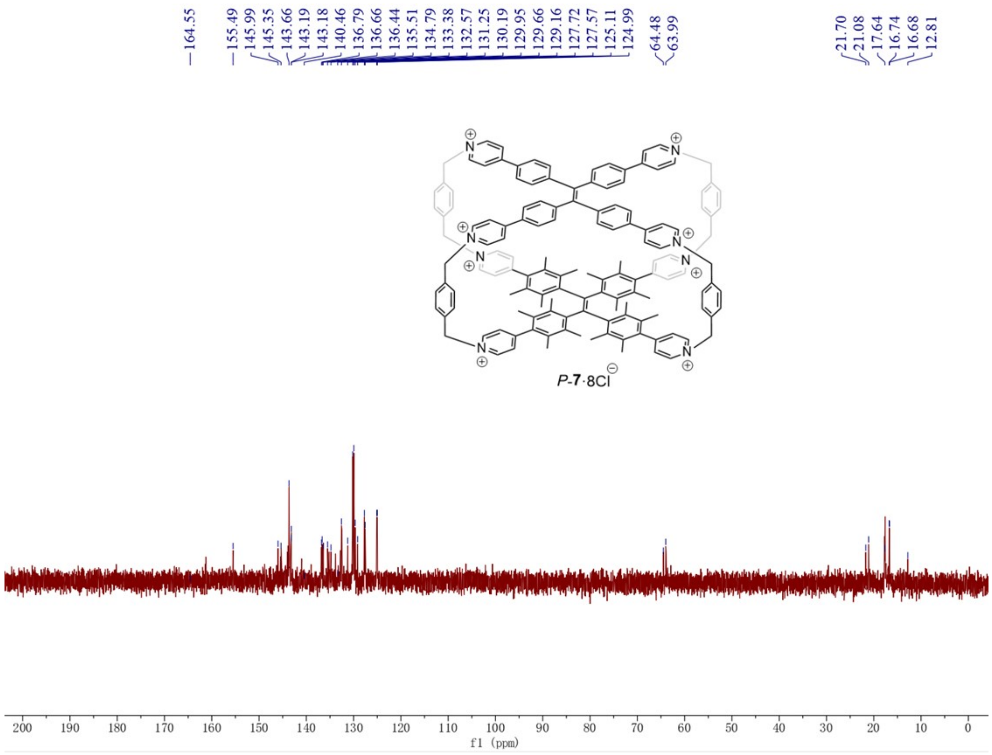

Figure S74. ^{13}C NMR spectrum of *P*-**7**·8Cl⁻ (101 MHz, D_2O).

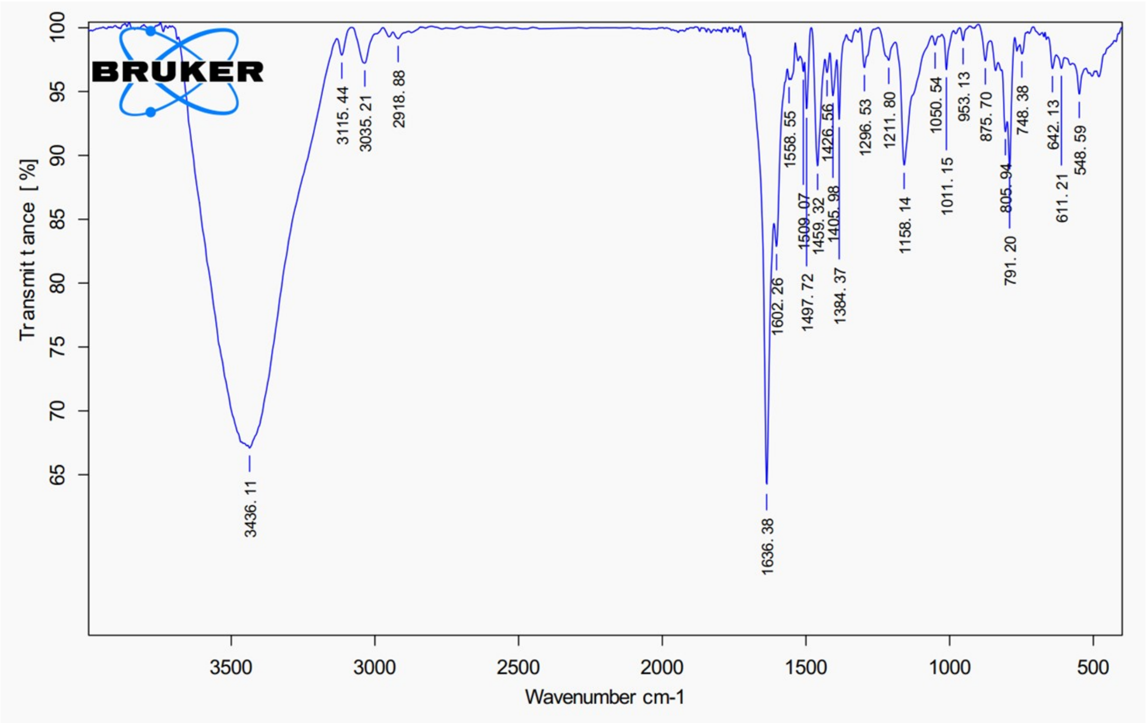

Figure S75. IR spectrum of *P*-**7**·8Cl⁻.

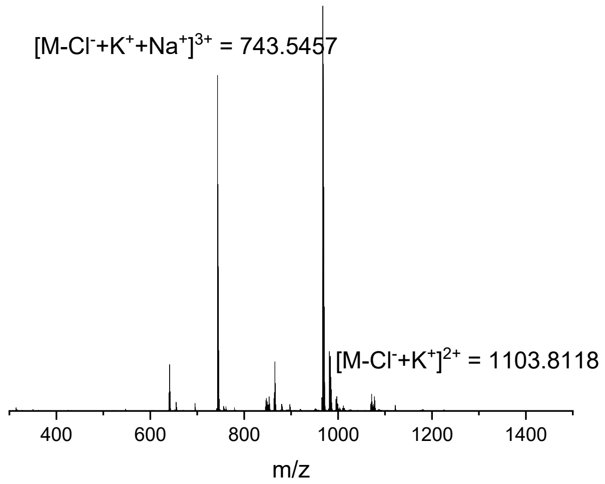

Figure S76. Experimental electrospray ionization mass spectra of *P*-**7**·8Cl⁻.

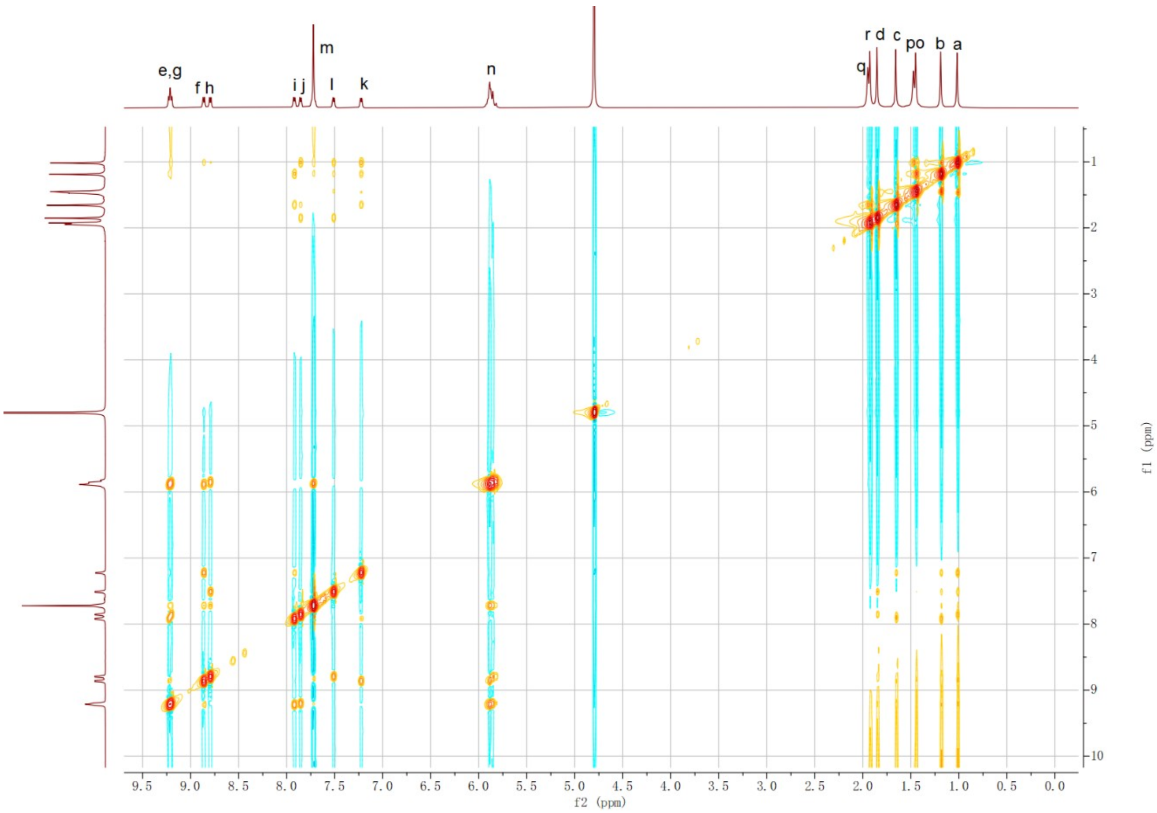

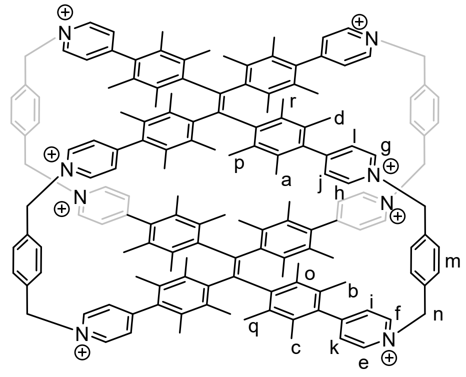

Figure S77. ^{1}H-^{1}H NOESY NMR spectrum of *PP*-**6**·8Cl^{-} in D$_2$O. Notes: The assignment of hydrogen atoms is determined jointly by the single-crystal structure and the ^{1}H-^{1}H NOESY NMR spectrum.

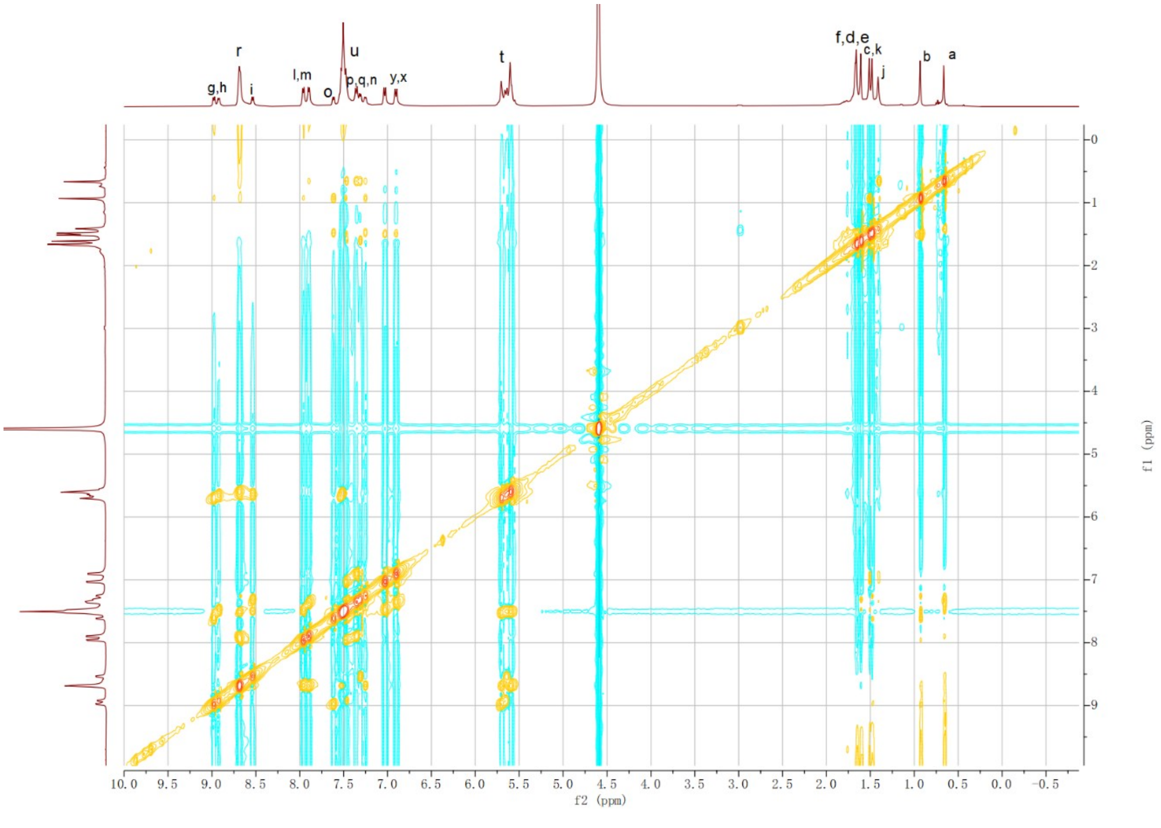

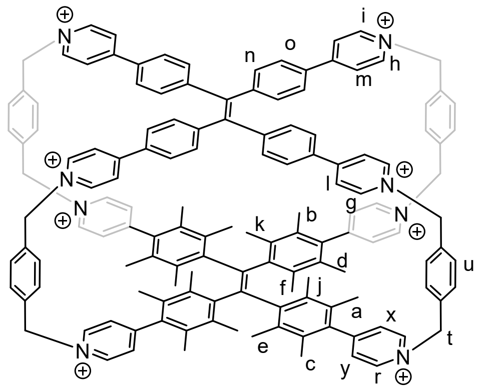

Figure S78. ^{1}H-^{1}H NOESY NMR spectrum of *P*-**7**·8Cl$^-$ in D_2O. Notes: The assignment of hydrogen atoms is determined jointly by the single-crystal structure and the ^{1}H-^{1}H NOESY NMR spectrum. Regarding the assignment of hydrogen atoms on the TPE unit, due to the overlap of the peak positions of most hydrogen atoms, only a few hydrogen atoms can be identified.

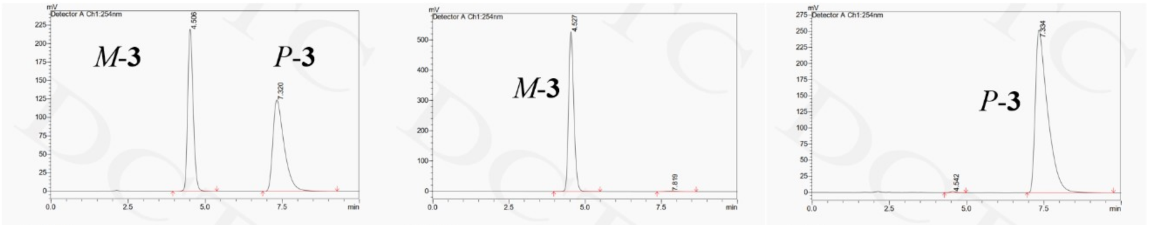

<Peak Table>

Peak#	Ret. Time	Area	Area%	T.Plate#	Tailing F.	Resolution
1	4.506	2697181	46.2132	3007.860	1.261	--
2	7.320	3139210	53.7868	1944.456	1.706	5.669

<Peak Table>

Peak#	Ret. Time	Area	Area%	T.Plate#	Tailing F.	Resolution
1	4.527	6344108	99.4599	3183.594	1.258	--
2	7.819	34451	0.5401	2098.243	1.251	6.560

<Peak Table>

Peak#	Ret. Time	Area	Area%	T.Plate#	Tailing F.	Resolution
1	4.542	20361	0.2896	3224.886	1.182	--
2	7.334	7011049	99.7104	1474.367	2.087	5.152

Figure S79. Chiral HPLC analysis of (A) **3**, (B) *M*-**3** and (C)*P*-**3**.

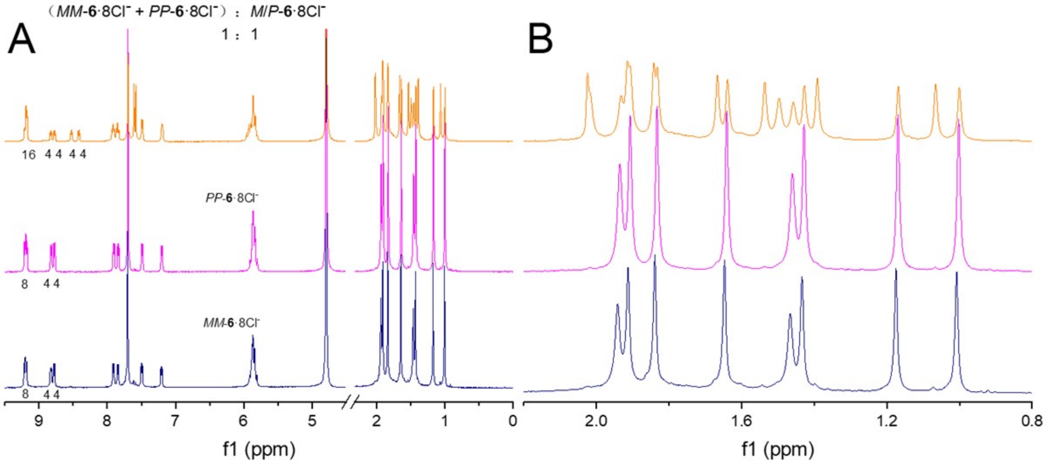

Figure S80. ^{1}H NMR spectra of *MM*-**6**·8Cl$^-$, *PP*-**6**·8Cl$^-$, and **6**·8Cl$^-$ in D_2O, displayed as (A) full spectrum and (B) partial spectrum of high field.

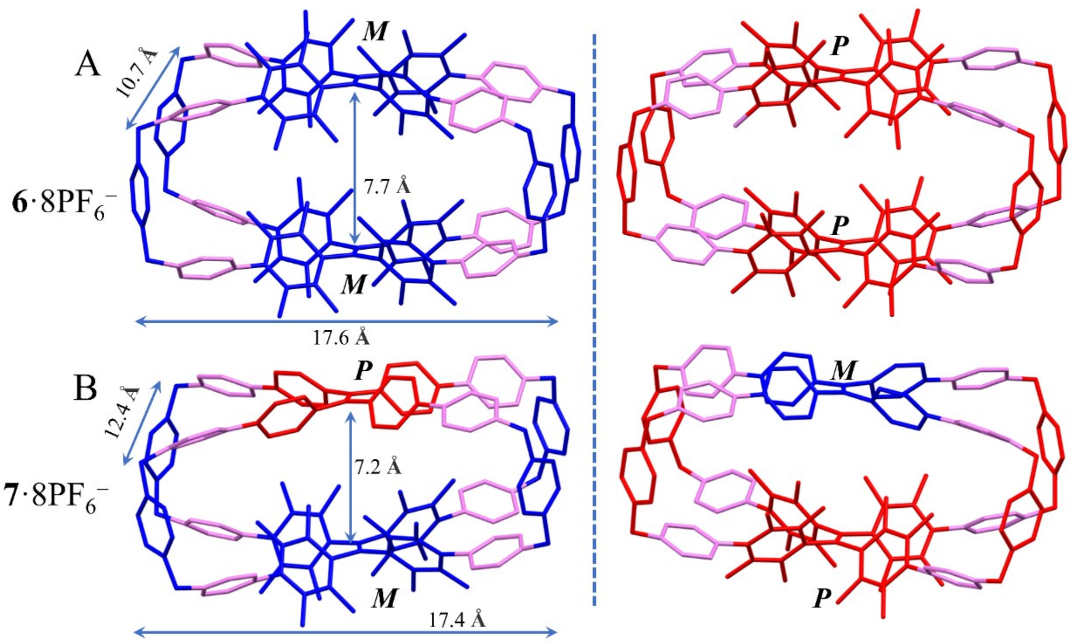

Figure S81. X-ray crystal structures of (A) racemic **6**·$8PF_6^-$ and (B) racemic **7**·$8PF_6^-$. Counterions, solvents and hydrogen atoms have been omitted for clarity.

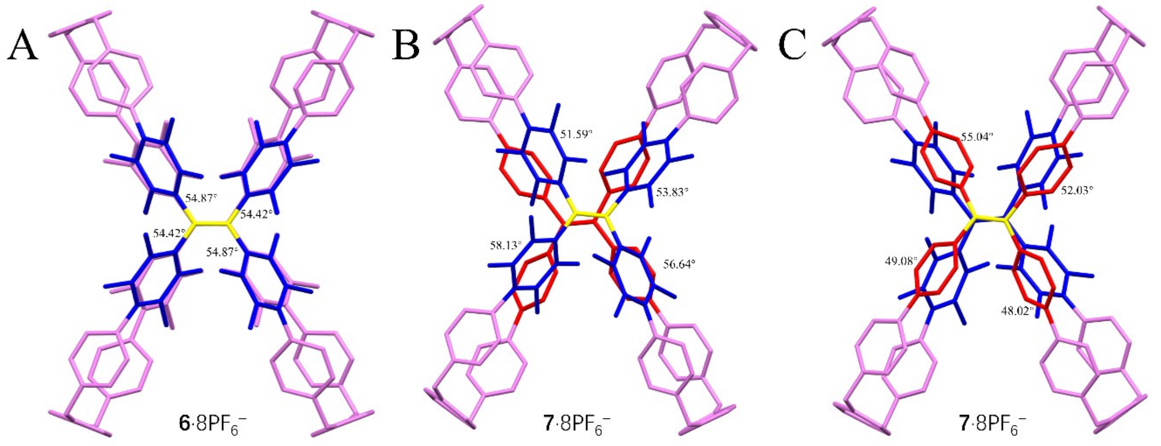

Figure S82. (A) The dihedral angle between the double-bond plane and the phenyl rings of **6**·$8PF_6^-$. (B) The dihedral angle between the h-TPE double-bond plane and the phenyl rings of **7**·$8PF_6^-$. (C) The dihedral angle between the TPE double-bond plane and the phenyl rings of **7**·$8PF_6^-$. Counterions, solvents and hydrogen atoms have been omitted for clarity.

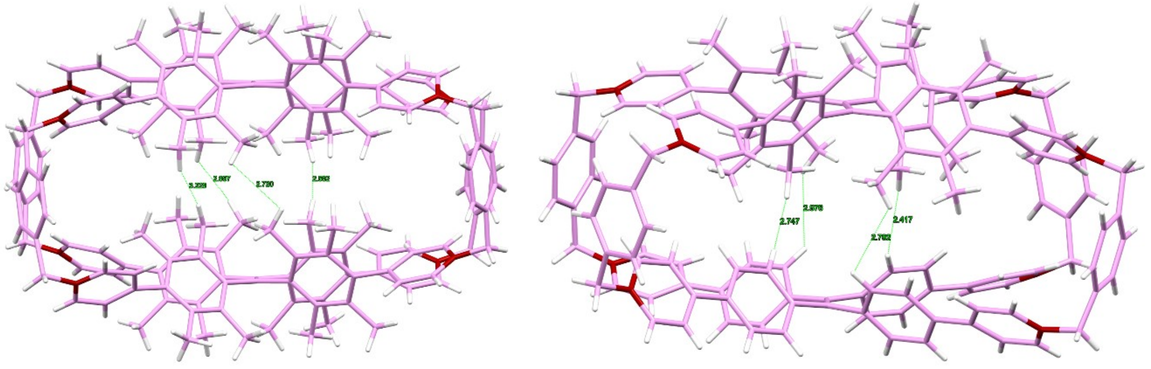

6·8PF_6^- **7**·8PF_6^-

Figure S83. The X-ray crystal structures revealed that the actual internal cavity height in **6**·8PF_6^- and **7**·8PF_6^-. Counterions and solvents have been omitted for clarity.

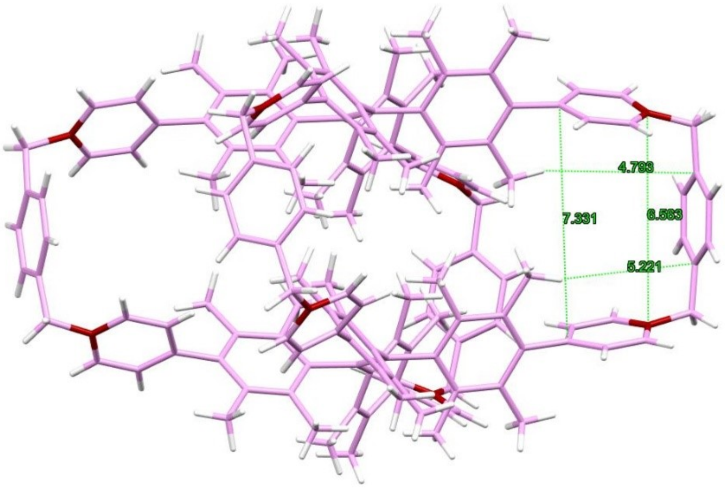

Figure S84. The X-ray crystal structures revealed that *p*-phenyl pillar columns and pyridine rings together form a macrocyclic structure in **6**·8PF_6^-. Counterions and solvents have been omitted for clarity.

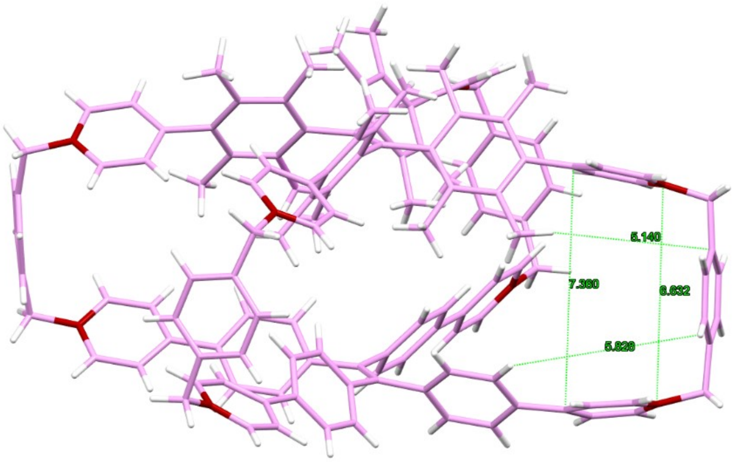

Figure S85. The X-ray crystal structures revealed that *p*-phenyl pillar columns and pyridine rings together form a macrocyclic structure in **7**·$8PF_6^-$. Counterions and solvents have been omitted for clarity.

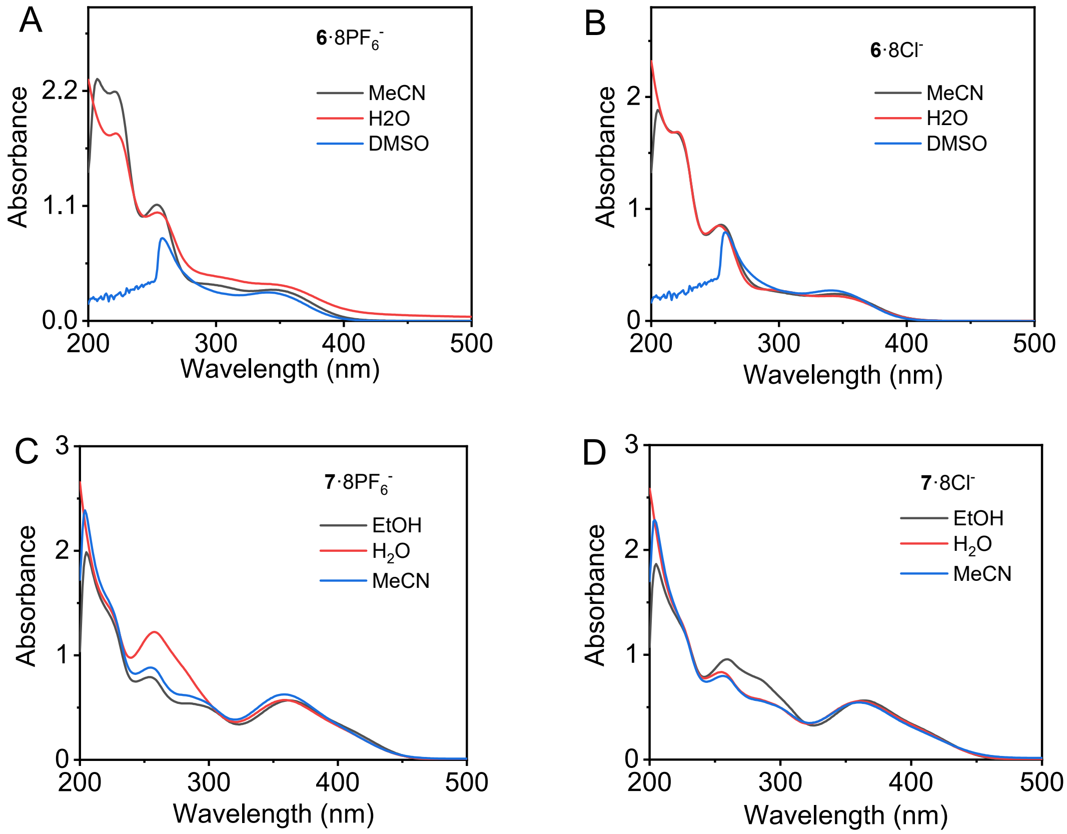

Figure S86. UV-Vis spectra of (A) **6**·8PF_6^-, (B) **6**·8Cl^-, (C) **7**·8PF_6^- and (D) **7**·8Cl^- in different solvents. ([c] = 1 × 10^{-5} M).

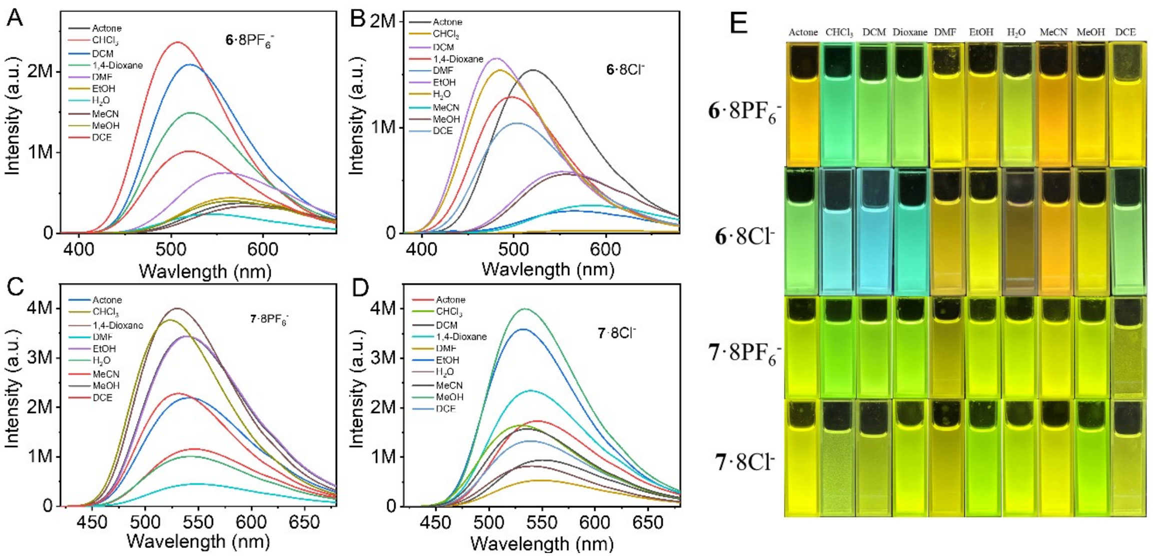

Figure S87. Emission spectra of (A) **6**·8PF$_6^-$, (B) **6**·8Cl$^-$, (C) **7**·8PF$_6^-$, (D) **7**·8Cl$^-$ in different solvents were presented, along (E) with their corresponding fluorescence photographs under UV 365 nm light. ([c] = 1.0 × 10^{-5} M, λ_{ex} = 350 nm).

Table S1. The absolute fluorescence quantum yield (Φ_F, %) and emission maximum wavelength (λ_{em}) of **6**·8PF$_6^-$, **6**·8Cl$^-$, **7**·8PF$_6^-$ and **6**·8Cl$^-$ in different solvents. ([c] = 1.0 × 10^{-5} M, λ_{ex} = 350 nm)

Solvent	**6**·8PF$_6^-$		**6**·8Cl$^-$		**7**·8PF$_6^-$		**7**·8Cl$^-$	
	Φ_F (%)	λ_{em} (nm)	Φ_F (%)	λ_{em} (nm)	Φ_F (%)	λ_{em} (nm)	Φ_F (%)	λ_{em} (nm)
MeCN	12.9	582	10.3	586	23.0	545	28.0	551
MeOH	15.3	566	20.9	559	63.4	530	67.5	533
Actone	14.3	575	20.7	521	43.8	532	23.4	545
H_2O	7.2	544	0.78	582	19.4	544	19.0	551
DMSO	29.5	559	24.6	558	60.0	539	59.7	539
$CHCl_3$	68.9	506	46.3	485	58.3	524	59.4	530

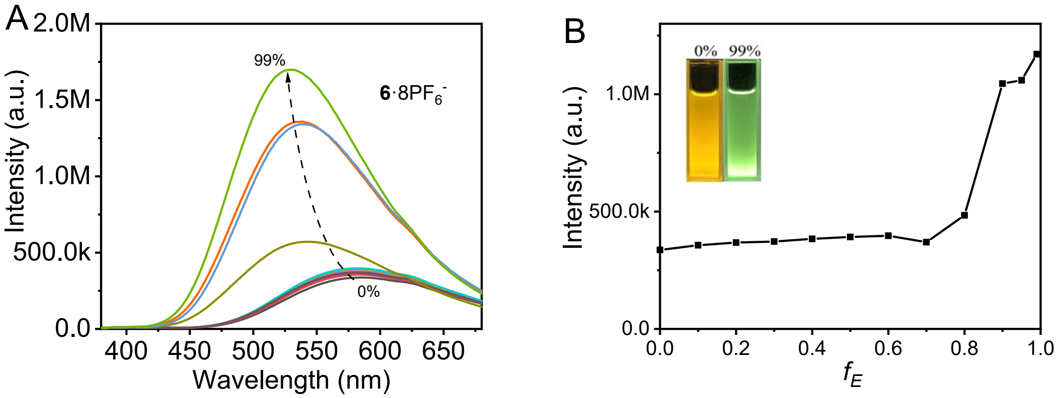

Figure S88. Fluorescence spectra of (A) **6**·8PF_6^- in MeCN/Et_2O mixture with different Et_2O fractions (f_E%); (B) Plot of maximum emission intensity of **6**·8PF_6^- versus Et_2O fraction in MeCN/Et_2O mixture, inset: photos taken under 365 UV light. (λ_{ex} = 350 nm, [**6**·8PF_6^-] = 1 × 10^{-5} M).

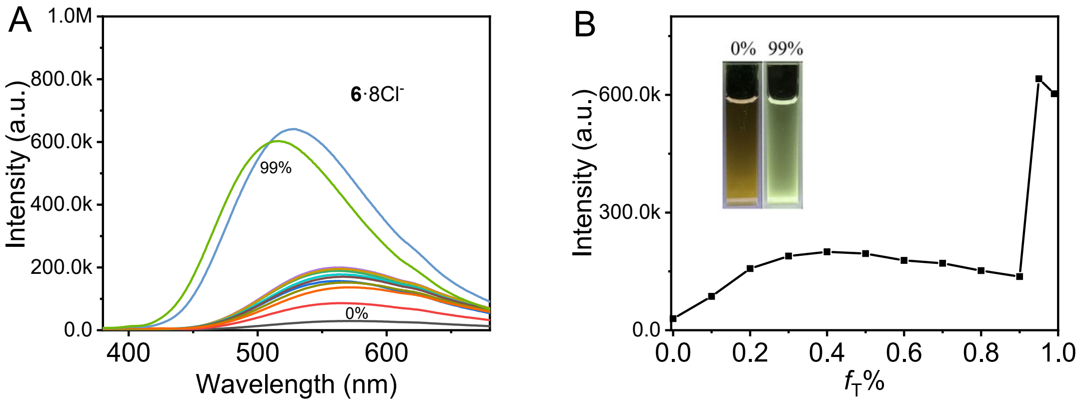

Figure S89. Fluorescence spectra of (A) **6**·8Cl^- in H_2O/THF mixture with different THF fractions (f_T%); (B) Plot of maximum emission intensity of **6**·8Cl^- versus H_2O fraction in H_2O/THF mixture, inset: photos taken under 365 UV light. (λ_{ex} = 350 nm, [**6**·8Cl^-] = 1 × 10^{-5} M).

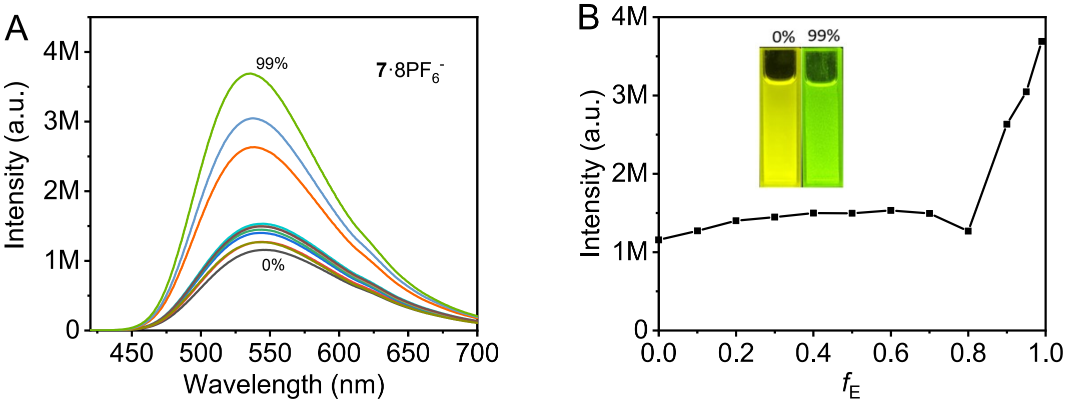

Figure S90. Fluorescence spectra of (A) $\mathbf{7}\cdot 8PF_6^-$ in MeCN/Et_2O mixture with different Et_2O fractions (f_E%); (B) Plot of maximum emission intensity of $\mathbf{7}\cdot 8PF_6^-$ versus Et_2O fraction in MeCN/Et_2O mixture, inset: photos taken under 365 UV light. (λ_{ex} = 350 nm, [$\mathbf{7}\cdot 8PF_6^-$] = 1 × 10^{-5} M).

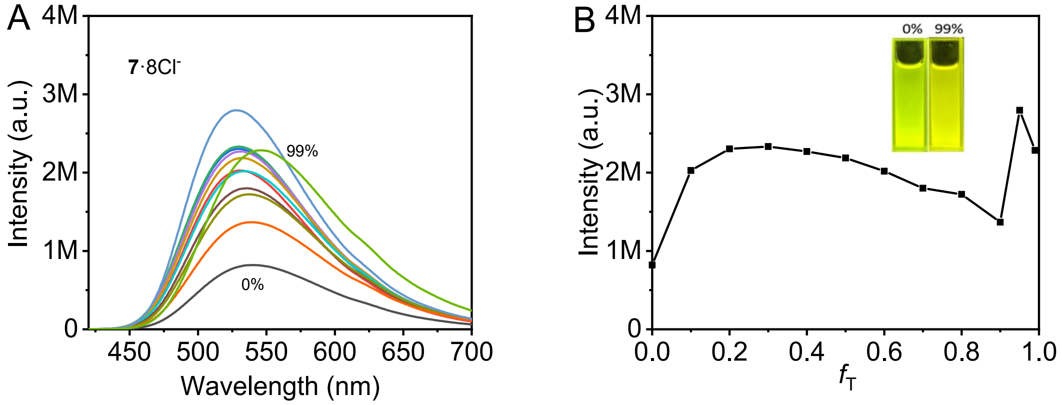

Figure S91. Fluorescence spectra of (A) $\mathbf{7}\cdot 8Cl^-$ in H_2O/THF mixture with different THF fractions (f_T%); (B) Plot of maximum emission intensity of $\mathbf{7}\cdot 8Cl^-$ versus H_2O fraction in H_2O/THF mixture, inset: photos taken under 365 UV light. (λ_{ex} = 350 nm, [$\mathbf{7}\cdot 8Cl^-$] = 1 × 10^{-5} M).

Table S2. Solid absolute fluorescence quantum yield (Φ_F, %) and emission maximum wavelength (λ_{em}) of $\mathbf{6}\cdot 8PF_6^-$, $\mathbf{6}\cdot 8Cl^-$, $\mathbf{7}\cdot 8PF_6^-$ and $\mathbf{6}\cdot 8Cl^-$. (λ_{ex} = 350 nm).

Compound	Φ_F (%)	λ_{em} (nm)
$\mathbf{6}\cdot 8PF_6^-$	43.2	552
$\mathbf{6}\cdot 8Cl^-$	12.7	565
$\mathbf{7}\cdot 8PF_6^-$	31.4	557
$\mathbf{7}\cdot 8Cl^-$	23.2	570

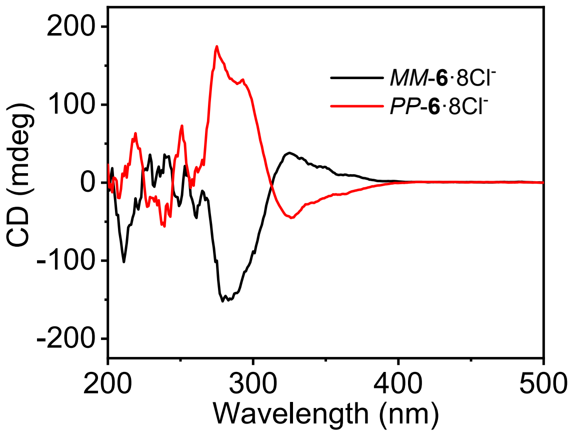

Figure S92. CD spectra of *MM*-**6**·8Cl⁻ and *PP*-**6**·8Cl⁻ in H_2O ([*MM*-**6**·8Cl⁻] = [*PP*-**6**·8Cl⁻] =1.0 ×10^{-3} M).

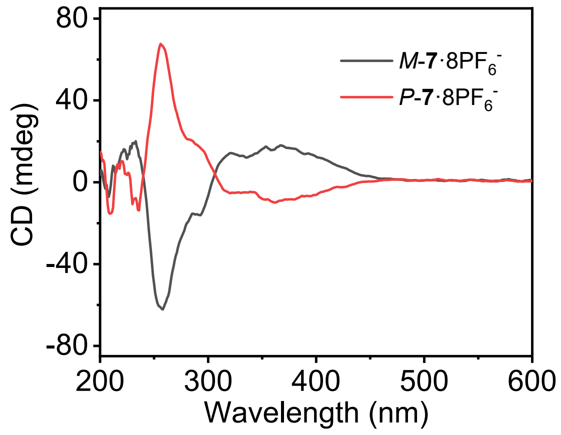

Figure S93. CD spectra of *M*-**7**·8PF_6^- and *P*-**7**·8PF_6^- in MeCN ([*M*-**7**·8PF_6^-] = [*P*-**7**·8PF_6^-] =5.0 ×10^{-4} M).

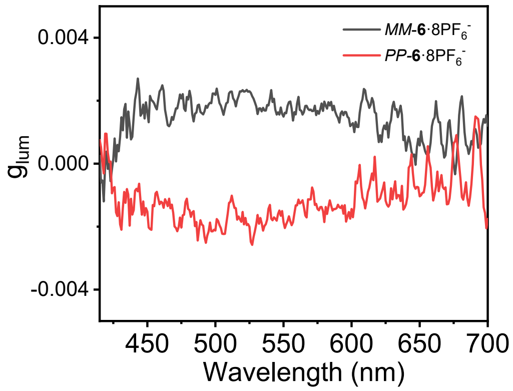

Figure S94. The g_{lum} spectra of *MM*-**6**·8PF_6^- and *PP*-**6**·8PF_6^- in MeCN/$CHCl_3$ suspension (10/90, v/v, [*MM*-**6**·8PF_6^-] = [*PP*-**6**·8PF_6^-] = 1 ×10^{-3} M).

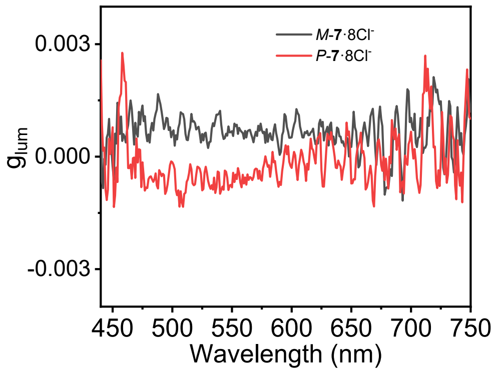

Figure S95. The g_{lum} spectra of *M*/*P*-**7**·8Cl^- in H_2O. ([*M*-**7**·8Cl^-] = [*P*-**7**·8Cl^-] = 1.0 × 10^{-3} M)

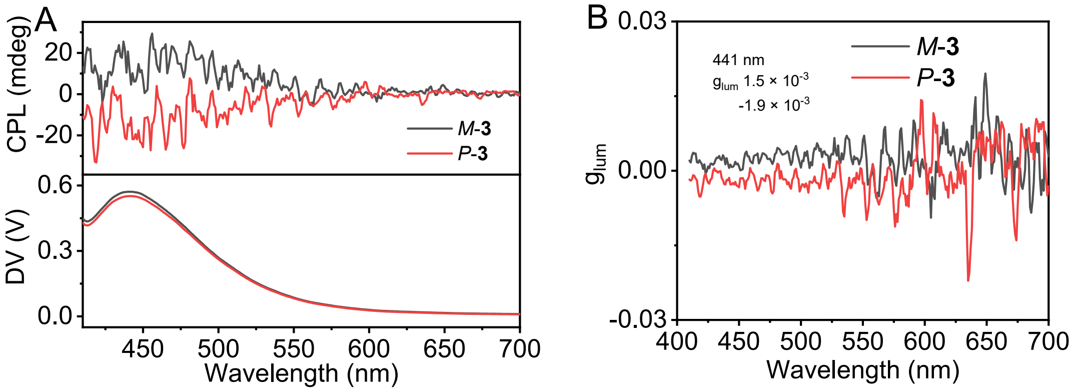

Figure S96. CPL (A) and g_{lum} (B) spectra of *M*-**3** and *P*-**3** in DMSO solution. ([*M*-**3**] = [*P*-**3**] 1.0 × 10^{-3} M)

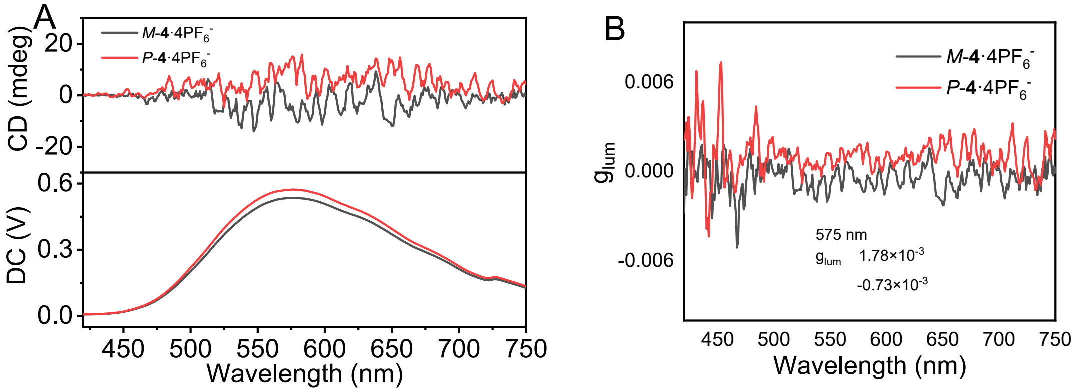

Figure S97. CPL (A) and g_{lum} (B) spectra of *P*-**4**·4PF_6^- and *M*-**4**·**4**PF_6^- in DMSO solution. ([c] = 1.0 × 10^{-3} M)

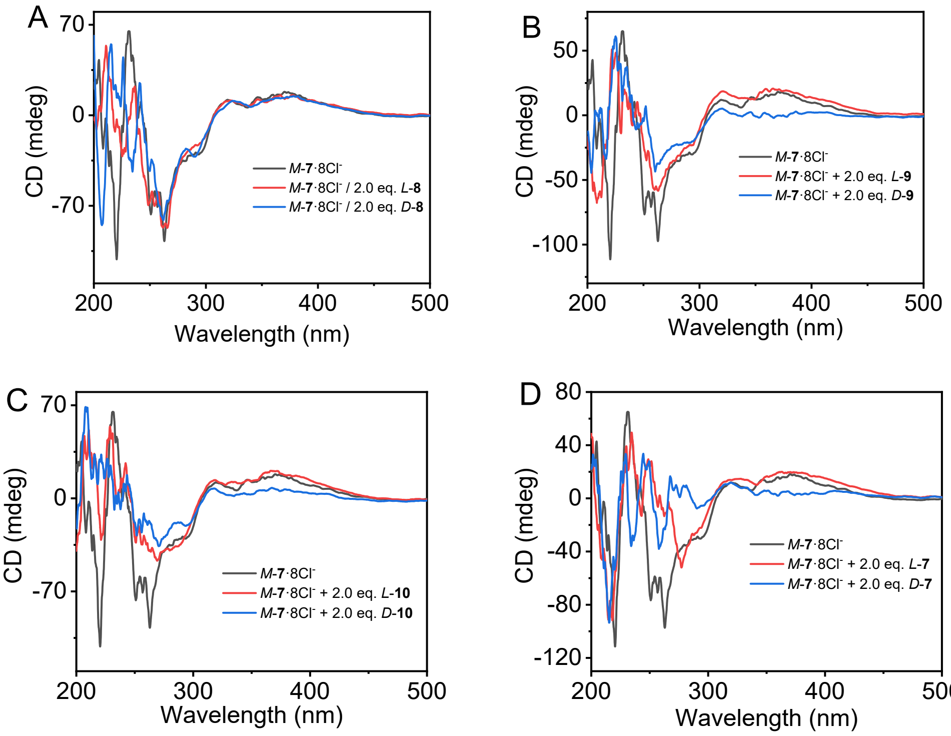

Figure S98. CD spectra of *M*-**7**·8Cl^- with 2.0 eq. of (A) *L*/*D*-**8**, (B) *L*/*D*-**9**, (C) *L*/*D*-**10** and (D) *L*/*D*-**11** in H_2O. ([*M*-**7**·8Cl^-] = 5.0 × 10^{-4}M)

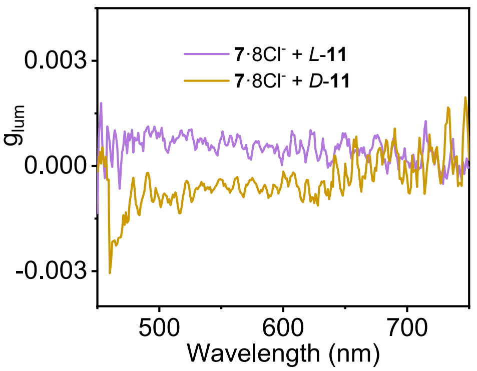

Figure S99. g_{lum} spectra of **7**·8Cl⁻ with 3.0 eq. *L*/*D*-**11** in H_2O. ([**7**·8Cl⁻] = 1.0 × 10^{-3}M)

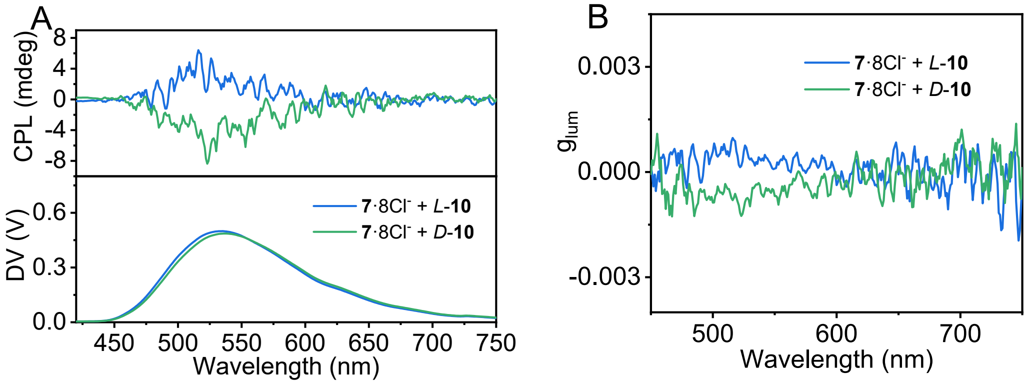

Figure S100. (A) CPL and (B) g_{lum} spectra of **7**·8Cl⁻ with 3.0 eq. *L*/*D*-**10** in H_2O. ([**7**·8Cl⁻] = 1.0 × 10^{-3}M)

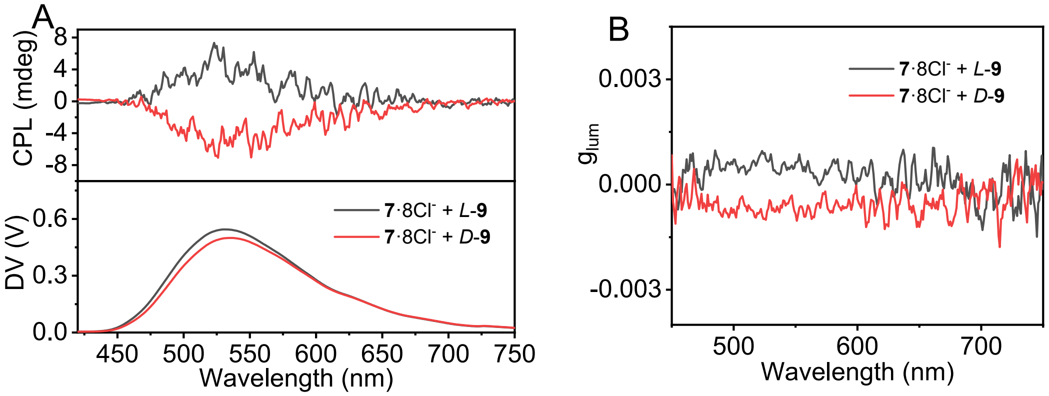

Figure S101. (A) CPL and (B) g_{lum} spectra of **7**·8Cl⁻ with 3.0 eq. *L*/*D*-**9** in H_2O. ([**7**·8Cl⁻] = 1.0 × 10^{-3}M)

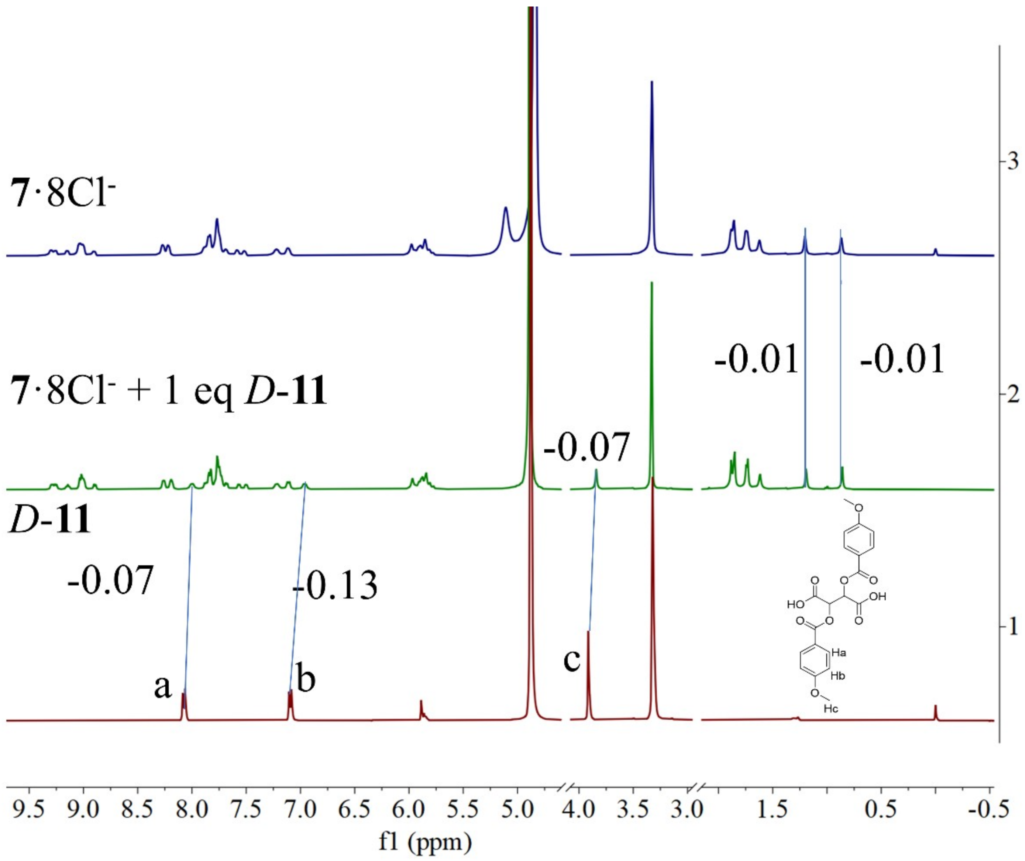

Figure S102. ^{1}H NMR spectra of **7**·8Cl⁻, *D*-**11**, **7**·8Cl⁻ and 1 eq. *D*-**11** in CD_3OD/D_2O (50/50, v/v). [**7**·8Cl⁻] = [*D*-**11**] = 1.0×10^{-3} M.

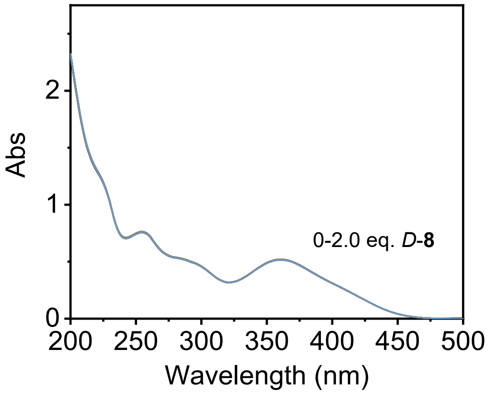

Figure S103. UV-Vis of **7**·8Cl⁻ titraed with *D*-**8** (0-2.0 eq.) in H_2O. ([**7**·8Cl⁻] = 1×10^{-5}M).

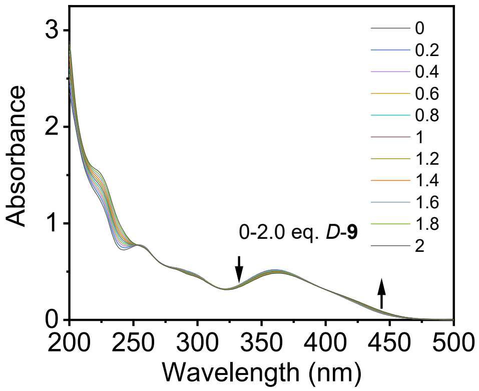

Figure S104. UV-Vis of **7**·8Cl⁻ titraed with *D*-**9** (0-2.0 eq.) in H_2O. ([**7**·8Cl⁻] = 1 × 10^{-5}M)

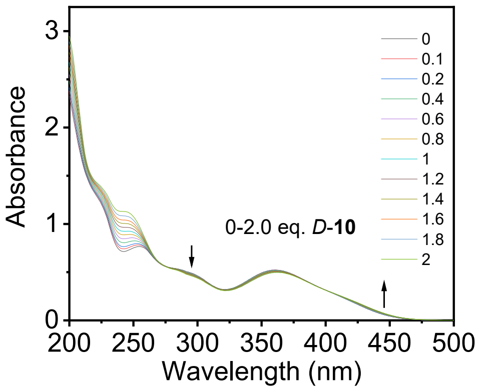

Figure S105. UV-Vis of **7**·8Cl⁻ titraed with *D*-**10** (0-2.0 eq.) in H_2O. ([**7**·8Cl⁻] = 1 × 10^{-5}M)

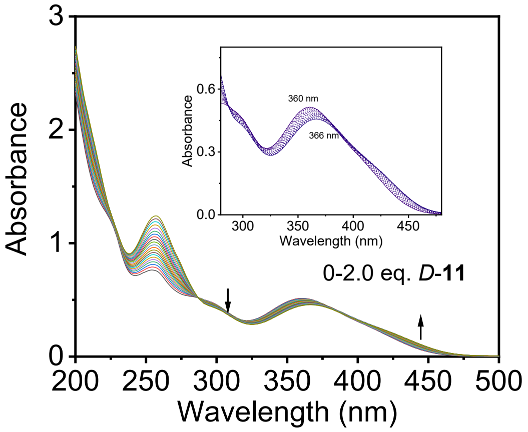

Figure S106. UV-Vis spectra of **7**·8Cl⁻ titraed with *D*-**11** (0-2.0 equiv) in H_2O ([**7**•8Cl⁻] = 1.0 × 10^{-5}M). (Inset) Partial enlarged image showing details of 300 – 450 nm region.

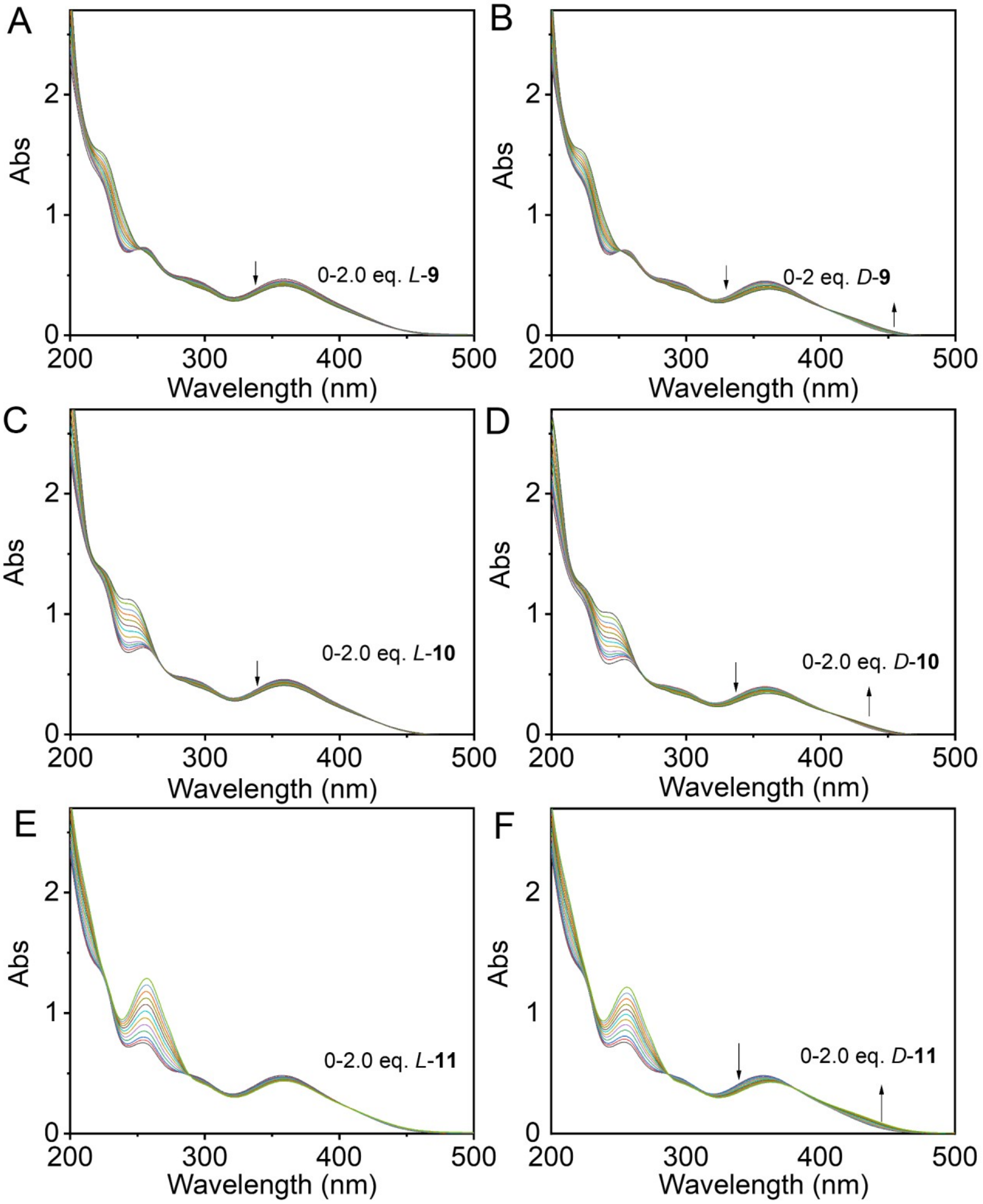

Figure S107. UV-Vis of *M*-**7**·8Cl⁻ titraed with (0-2.0 eq.) (A) *L*-**9**, (B) *D*-**9**, (C) *L*-**10**, (D) *D*-**10**, (E) *L*-**11** and (F) *D*-**11** in H_2O. ([**7**·8Cl⁻] = 1 × 10^{-5}M)

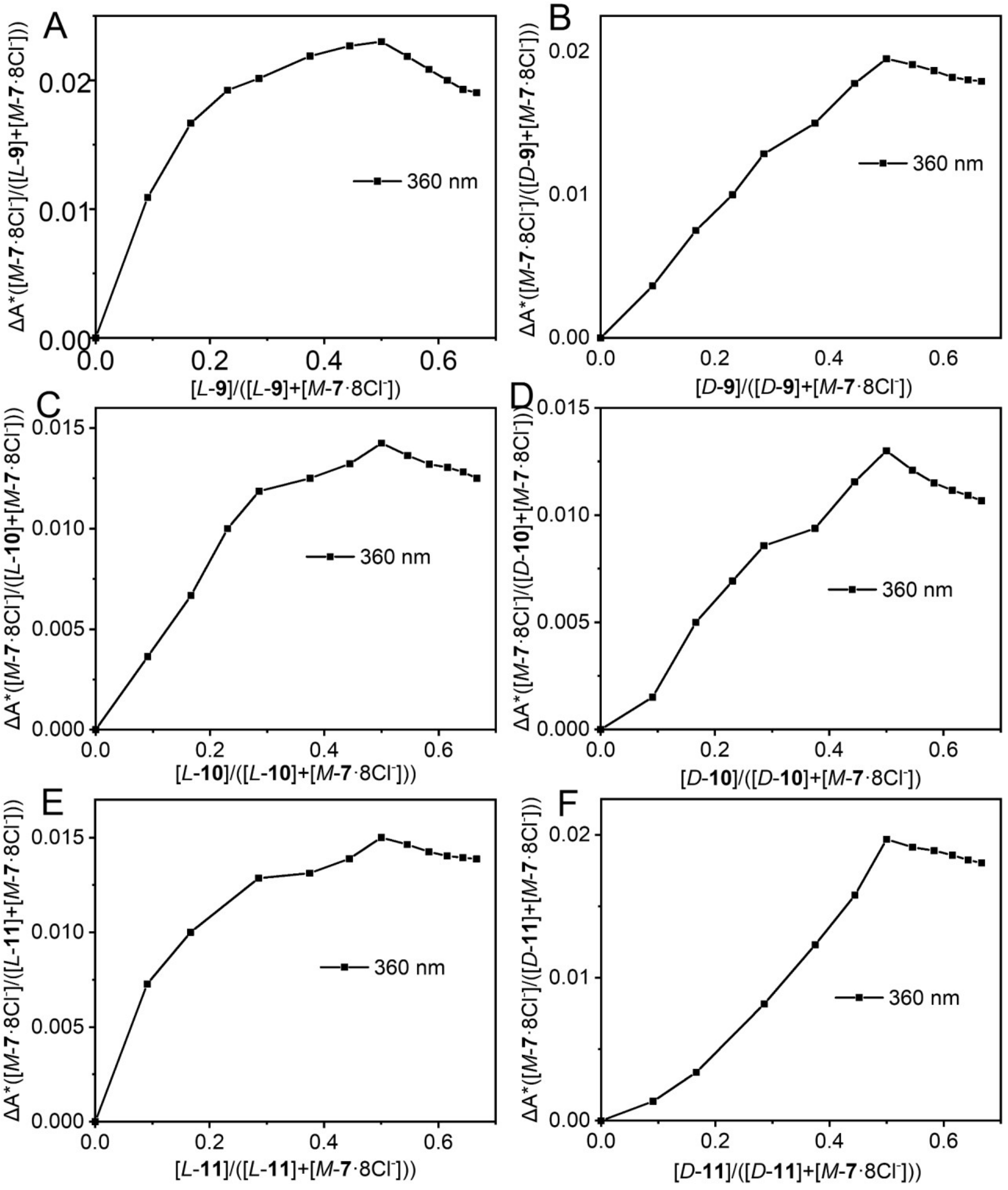

Figure S108. Job plot analysis for the formation of host-guest complexes between *M*-**7**·8Cl⁻ and (A) *L*-**9**, (B) *D*-**9**, (C) *L*-**10**, (D) *D*-**10**, (E) *L*-**11** and (F) *D*-**11** in H_2O, showing a 1:1 binding in solution.

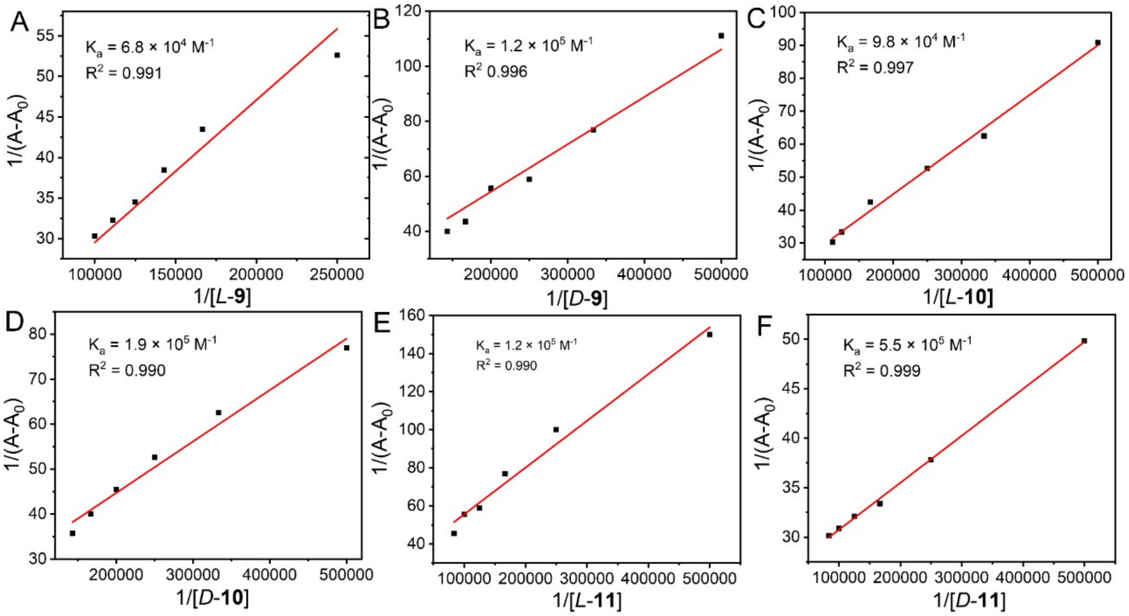

Figure S109. The linear curve-fitting (UV titrations) for the host–guest complexation of *M*-**7**·8Cl$^-$ with different concentration of *L*-**9** (A), *D*-**9** (B), *L*-**10** (C), *D*-**10** (D), *L*-**11** (E), *D*-**11** (F),the binding constant was calculated by Benesi-Hildebrand equation for 1:1 association.

Table S3. Quantum yield (Φ_F, %) of **7**·8Cl$^-$⊃*D*-**9**, **7**·8Cl$^-$⊃*D*-**10** and **7**·8Cl$^-$⊃*D*-**11** in water. (λ_{ex} = 350 nm).

Compound	Φ_F (%)
**7**·8Cl$^-$⊃*D*-**9**	45.5
**7**·8Cl$^-$⊃*D*-**10**	45.7
**7**·8Cl$^-$⊃*D*-**11**	49.8

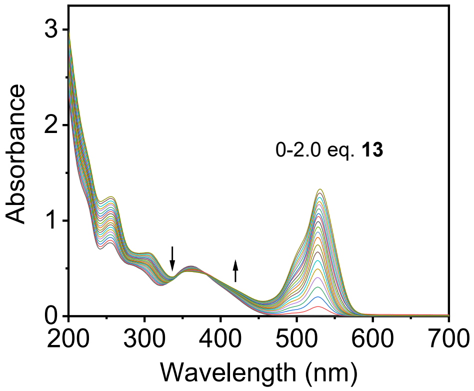

Figure S110. UV-Vis spectra of **7**·8Cl⁻ titraed with **13** (0-2.0 eq.) in H_2O. ([**7**·8Cl⁻] = 1 × 10^{-5}M).

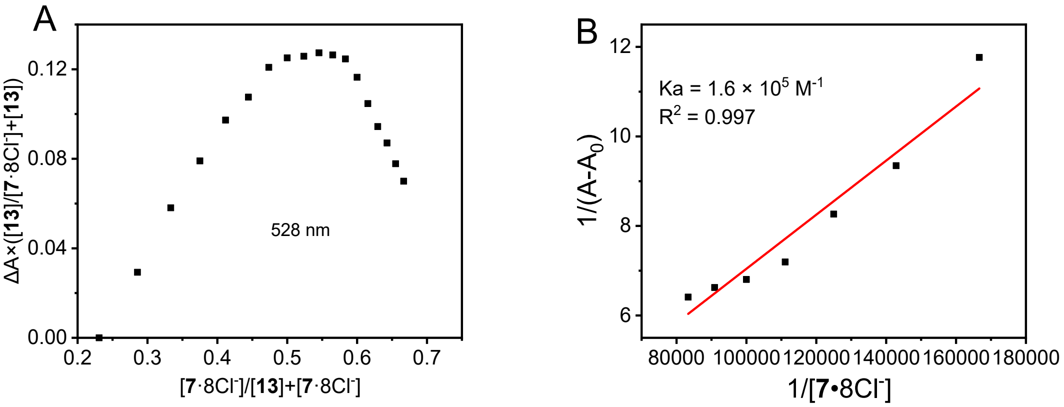

Figure S111. (A) Job plot analysis for the formation of host-guest complexes between **7**·8Cl⁻ and **13** in water, the absorbance recorded at 528 nm showing a 1:1 binding in solution. (B) The linear curve-fitting (UV titrations) for the host–guest complexation of **13** with different concentration of **7**·8Cl⁻, the binding constant was calculated by Benesi-Hildebrand equation for 1:1 association.

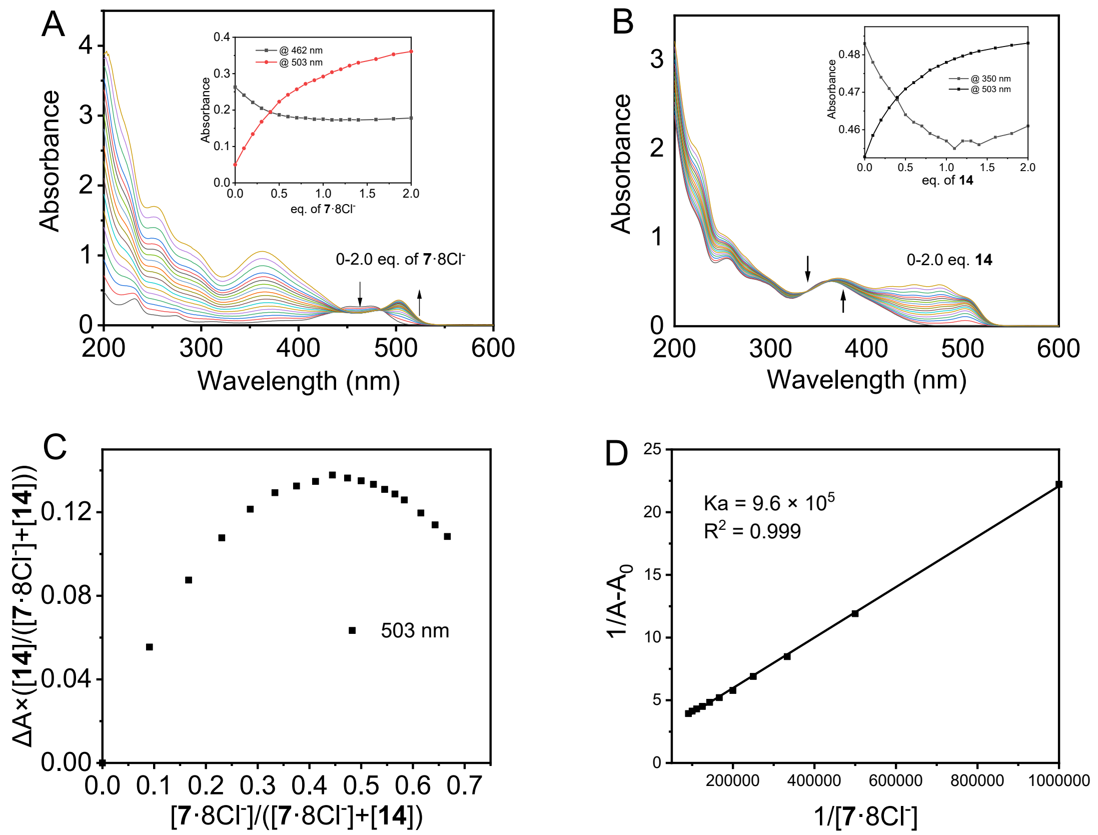

Figure S112. (A) UV-Vis spectra of **14** titraed with **7**·8Cl⁻ (0-2.0 eq.) in H_2O. Inset: plots the absorbance 462 nm and 503 nm *versus* eq. of **7**·8Cl⁻. ([**14**] = 1.0 × 10^{-5}M). (B) UV-Vis spectra of **7**·8Cl⁻ titraed with **14** (0-2.0 eq.) in H_2O. Inset: plots the absorbance 350 nm and 503 nm *versus* equiv. of Fluorescein. ([**7**·8Cl⁻] = 1.0 × 10^{-5}M). (C) Job plot for the UV-Vis titration of **14** with **7**·8Cl⁻ in H_2O. The absorbance recorded at 503 nm, showing a 1:1 binding in solution. (D) The linear curve-fitting (UV titrations) for the host–guest complexation of **14** with different concentration of **7**·8Cl⁻, the binding constant was calculated by Benesi-Hildebrand equation for 1:1 association.

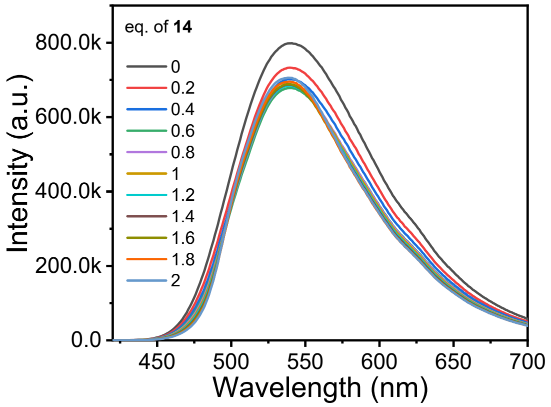

Figure S113. Fluorescence spectra of **7**·8Cl⁻ titraed with **14** (0-2.0 eq.) in H_2O. (λ_{ex} = 350 nm, [**7**·8Cl⁻] = 1.0×10^{-5}M).

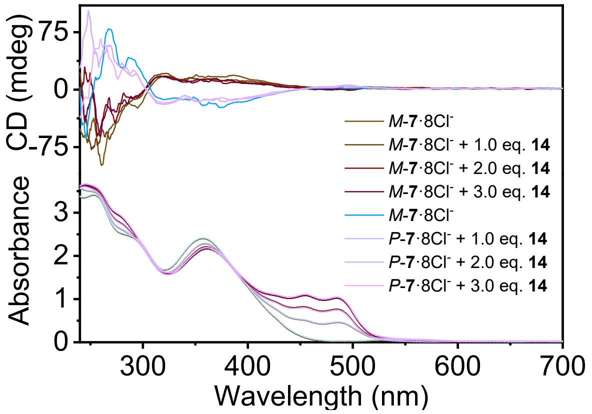

Figure S114. CD and absorbance spectra of *P*-**7**·8Cl⁻ and *M*-**7**·8Cl⁻ with **14** (0-3.0 eq.) in H_2O. ([*P*-**7**·8Cl⁻] = [*M*-**7**·8Cl⁻] = 5 $\times 10^{-4}$ M).

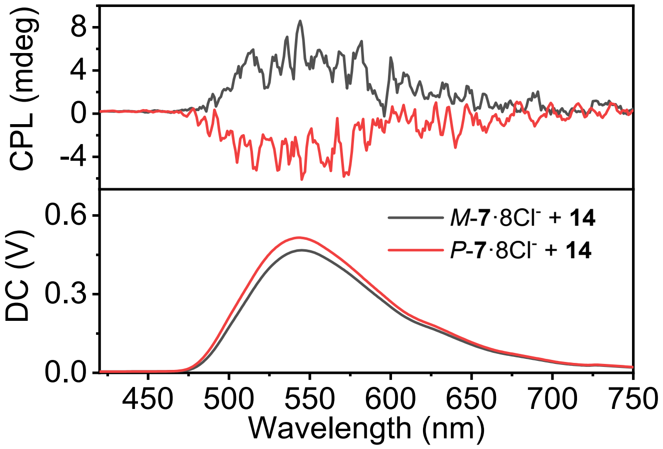

Figure S115. CPL spectra of *M*/*P*-**7**·8Cl⁻ with 1.0 eq. **14** in H_2O. ([*P*-**7**·8Cl⁻] = [*M*-**7**·8Cl⁻] = 1.0×10^{-3}M).

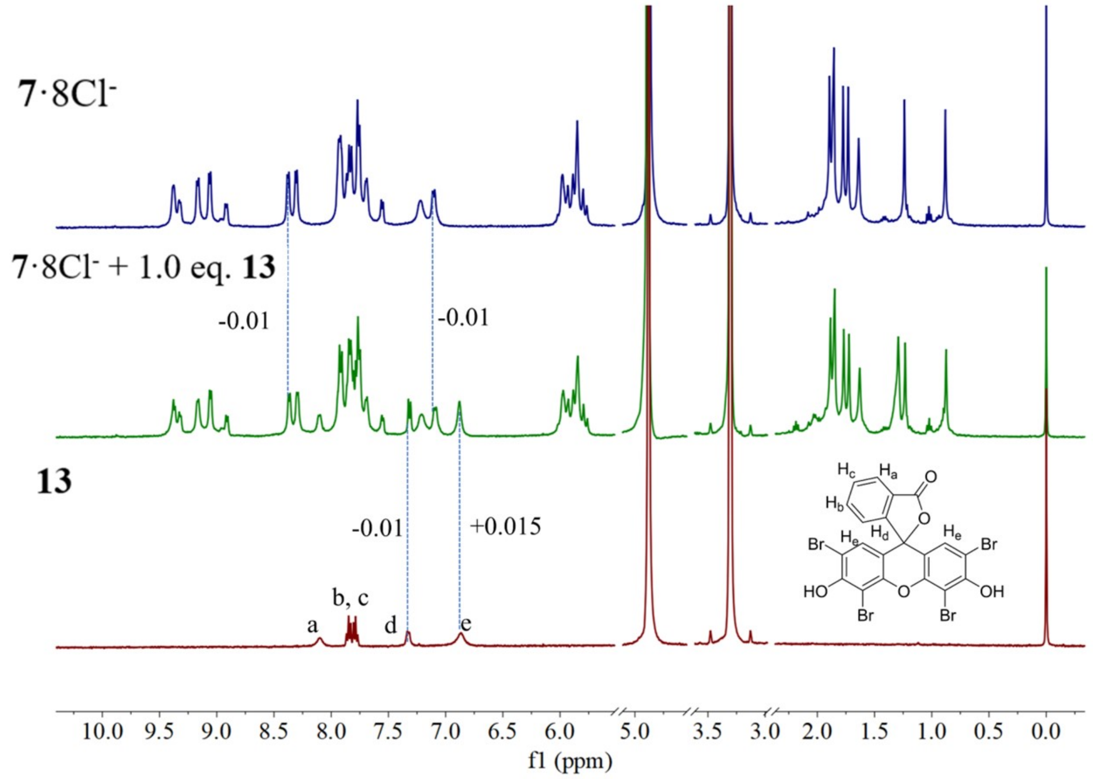

Figure S116. ^{1}H NMR spectra of **7**·8Cl⁻, **13**, **7**·8Cl⁻ and 1.0 eq. **13** in CD_3OD/D_2O (50/50, v/v). [**7**·8Cl⁻] = [**13**] = 2.0 × 10^{-3} M.
